# Common clonal hematopoiesis driver mutations have disparate effects on macrophage cytokines, clonal expansion, and atherogenesis.

**DOI:** 10.1172/jci.insight.200334

**Published:** 2025-12-23

**Authors:** Paul R. Carter, Lauren Kitt, Amanda C. Rodgers, Nichola Figg, Ang Zhou, Chengrui Zhu, Ziyang Wang, Peter Libby, Stephen Burgess, George S. Vassiliou, Murray C.H. Clarke

**Affiliations:** 1 Section of CardioRespiratory Medicine and; 2 Cardiovascular Epidemiology Unit, The Victor Phillip Dahdaleh Heart & Lung Research Institute, Cambridge Biomedical Campus, The University of Cambridge, Cambridge, United Kingdom.; 3 Cardiovascular Medicine Division, Brigham and Women’s Hospital, Boston, Massachusetts, USA.; 4 Cambridge Stem Cell Institute, Jeffrey Cheah Biomedical Centre, Cambridge Biomedical Campus, The University of Cambridge, Cambridge, United Kingdom.

**Keywords:** Immunology, Inflammation, Vascular biology, Atherosclerosis, Cytokines, Macrophages

## Abstract

Clonal hematopoiesis of indeterminate potential (CHIP) is the expansion of blood stem cells and progeny after somatic mutation. CHIP associates with increased cardiovascular disease (CVD), with inflammation from macrophages a proposed common effector. However, mouse CHIP studies are discordant for clonal expansion and inflammation. Similarly, directionality of association between CHIP and CVD remains debated. We investigated effects of 3 CHIP mutations on macrophage cytokines, clonal expansion, and atherosclerosis in parallel. We found that cytokine release and inflammasome activation are increased by *Tet2* mutation but decreased by *Dnmt3a*. However, *Jak2* mutant macrophages produced equivalent cytokine as WT. In mice, *Tet2* mutants clonally expanded, but *Dnmt3a* and *Jak2* mutants did not. Expansion was unaffected by systemic inflammation, while hyperlipidemia expanded *Tet2*^–/–^ cells but not mono-allelic mutants. Similarly, human Mendelian randomization showed no effect of serum cytokines or CVD on CHIP risk. Experimental atherosclerosis was increased in females with *Tet2* and males with *Jak2*, but it was unchanged with *Dnmt3a* mutations. Together, common CHIP mutations have disparate effects on macrophage cytokines and clonal expansion, and they have sex-dependent effects on atherogenesis, suggesting a common mechanism across CHIP is unlikely. Thus, CHIP mutations differ in pathophysiology and clinical sequelae across sexes and should be treated as different entities.

## Abstract 2

CHIP associates with diseases. We find CHIP mutations disparately alter cytokines, clonal expansion, and sex-dependently effects atherosclerosis - suggesting a common mechanism across CHIP is unlikely.

## Introduction

Clonal hematopoiesis of indeterminate potential (CHIP) refers to the expansion of somatically mutated hematopoietic stem cells (HSCs) and their progeny, resulting in leukocytes harboring these driver mutations in the circulation. CHIP prevalence rises steeply with age and is typically detected in ~2% of individuals at 40 years, ~12% at 80 years, and ~18% > 80 years (1–4). Commonly mutated genes (*TET2*, *DNMT3A*, *JAK2*) alter self-renewal and differentiation of HSCs, ultimately conferring a fitness advantage. TET2 is a DNA methylcytosine dioxygenase that broadly acts to reverse methylation-dependent gene silencing, with typical loss-of-function (LOF) mutations increasing methylation. DNMT3A is a DNA methytransferase that broadly acts to induce methylation-dependent gene silencing, and thus, typical LOF mutations decrease methylation. In contrast, JAK2 is a nonreceptor tyrosine kinase integral to cytokine signaling (e.g., IFN, growth factors, and IL-6), with typical gain-of-function (GOF) mutations inducing constitutive receptor activation. Importantly, the consequences of CHIP extend beyond hematopoiesis, with widely reported associations to oncological, respiratory, metabolic, and cardiovascular disease (CVD) risk (1, 4–8) and with increased inflammation from mutant leukocytes proposed as a common mediator.

Atherosclerosis remains the leading cause of CVD and mortality worldwide, with age being one of the strongest risk factors (9, 10) and inflammation (particularly via IL-1) powerfully driving disease (11). CHIP prevalence increases with age and associates with increased risk of multiple CVDs across the common CHIP mutations (1, 5, 8, 12–15). Experimentally, *Ldlr*^–/–^ mice transplanted with *Tet2*^–/–^ (5, 16–18), *Dnmt3a^–/–^*(19), or *Jak2*^VF^ (20, 21) BM develop more atherosclerosis, suggesting a causative effect of CHIP. The larger plaques witnessed in *Tet2*^–/–^ CHIP models were also recapitulated with myeloid-specific *Tet2* deletion, with NLRP3 inflammasome inhibitors abolishing increased plaque (16). Myeloid-specific *Dnmt3a* deletion also increased plaque size and macrophage (Mɸ) content in *Ldlr*^–/–^ recipients (19). Similarly, transplantation of *Jak2*^VF^ cells increased plaque, neutrophil content, and necrotic cores (20), which was dependent on caspase-1 or -11 via the AIM2 inflammasome (21). This suggests that Mɸ-derived IL-1β and/or IL-18 (as both are inflammasome activated) are likely common effectors of atherosclerosis in murine CHIP models. However, while a trend for improved CVD outcome in humans upon IL-1β neutralization occurs among those with *TET2* CHIP in CANTOS, those with *DNMT3A* were not protected (22). Although murine studies support CHIP driving atherogenesis, there are some limitations, including: predominant use of biallelic mutations that do not reflect monoallelic human CHIP; transplant of 100% mutant cells that does not model clonal growth, expansion of clones to levels not typically seen in humans, and/or no tracking of clonal expansion; study of a single sex only; use of Cre-drivers that are not Mɸ-specific (23); and use of Cre-only, floxed-only and/or an undefined mix as controls. Furthermore, humans with germline LOF *TET2* mutations do not show elevated atherosclerosis and CVD (24), and some observational and Mendelian randomization (MR) analyses do not find causal associations of CHIP on atherosclerosis (7, 14, 25–28). Thus, further studies are needed to assess if CHIP models accelerate atherogenesis in mice and if conclusions on causation from these studies can inform on the pathogenesis of CVD in humans with CHIP.

Because inflammation indubitably drives atherosclerosis, research has focused on the effect of CHIP mutations on inflammatory responses, particularly by Mɸs. Activated *Tet2*^–/–^ Mɸs show increased cytokine and *Nlrp3* transcript and release more IL-1β after inflammasome activation (5, 16). However, earlier studies not focused on CHIP did not see increased IL-1β with *Tet2* loss (29), or with *TET2* knockdown in human cells (24). Activated *Dnmt3a*^–/–^ Mɸs also show increased cytokine transcripts congruent with those in *Tet2*^–/–^ Mɸs, but they show only modest increases in pro–IL-1β^+^ cells (19). Also, it is unknown how LOF mutations in *Dnmt3a* and *Tet2* — genes with opposite effects on DNA methylation — both increase cytokines. *Jak2*^VF^ Mɸs also secrete more IL-6 and ~5- to 7-fold more IL-1β after inflammasome activation (20, 21), and *Jak2*^VF^ mice on high-fat diet (HFD) have higher serum IL-18 and caspase-1/11 activation in spleens (20). However, other studies report negligible effects of *Jak2* on inflammation (30, 31). Together, a clear scientific consensus for *Tet2*, *Dnmt3a*, and *Jak2* CHIP mutations promoting increased cytokines and inflammation that subsequently drives CVD does not currently exist; thus, further investigation is warranted.

The epidemiologic association between human CHIP and atherosclerosis is interpreted as CHIP driving atherogenesis (12). However, atherosclerosis and its associated inflammatory milieu may boost clonal growth (32, 33), as inflammation accelerates hematopoiesis, causing monocytosis and neutrophilia (34), while IL-1 augments HSC proliferation and myeloid differentiation (35, 36). Indeed, other inflammatory conditions (e.g., autoimmune and infection; refs. 37, 38) and atherogenic cytokines (e.g., TNF-α, IFN-γ, IL-6, and IL-1) associate with development of myeloid malignancies (39–43), which have mutations overlapping with CHIP (1, 5). Thus, atherosclerosis, which is characterized by chronic inflammation and BM turnover (44–47), could enable clonal expansion of mutant HSCs with a proliferative advantage. However, while experimental atherogenesis accelerates outgrowth of *Tet2*^–/–^ cells (32), human atherosclerosis does not seem to drive CHIP (12, 33). Thus, further studies assessing directionality of the CHIP-CVD relationship in both experimental and human systems are justified.

Here we provide a robust overview of the effects of 3 common CHIP mutations in parallel. We find that LOF *Tet2* and *Dnmt3a*^RH^ mutations have discordant effects, with increased cytokines and inflammasome activation with *Tet2* mutants and decreased with *Dnmt3a*^RH^ Mɸs. In contrast, GOF *Jak2*^VF^ mutant Mɸs show equivalent cytokine expression as WT. In mice, *Tet2* mutant cells clonally expand under the steady state, but *Dnmt3a*^RH^ and *Jak2*^VF^ cells do not. Clonal expansion is unaltered by IL-1–driven systemic inflammation, and although atherogenesis increased *Tet2*^–/–^ expansion, it did not affect models with monoallelic mutations. Similarly, MR analyses showed no effect of genetically predicted serum cytokines or CVD on risk of CHIP. Murine atherosclerosis in CHIP models was increased in females with *Tet2^+/–^* and males with *Jak2*^WT/VF^, but it was not changed with *Dnmt3a*^WT/RH^ mutant cells. These results support the notion that distinct mechanisms drive CHIP-associated diseases and emphasize the need to treat CHIP subtypes as different entities and to study the modifying effects of sex.

## Results

### Validation of genotype and basic phenotyping of Tet2, Jak2^VF^, and Dnmt3a^RH^ mutant mice.

**Figure 1:**
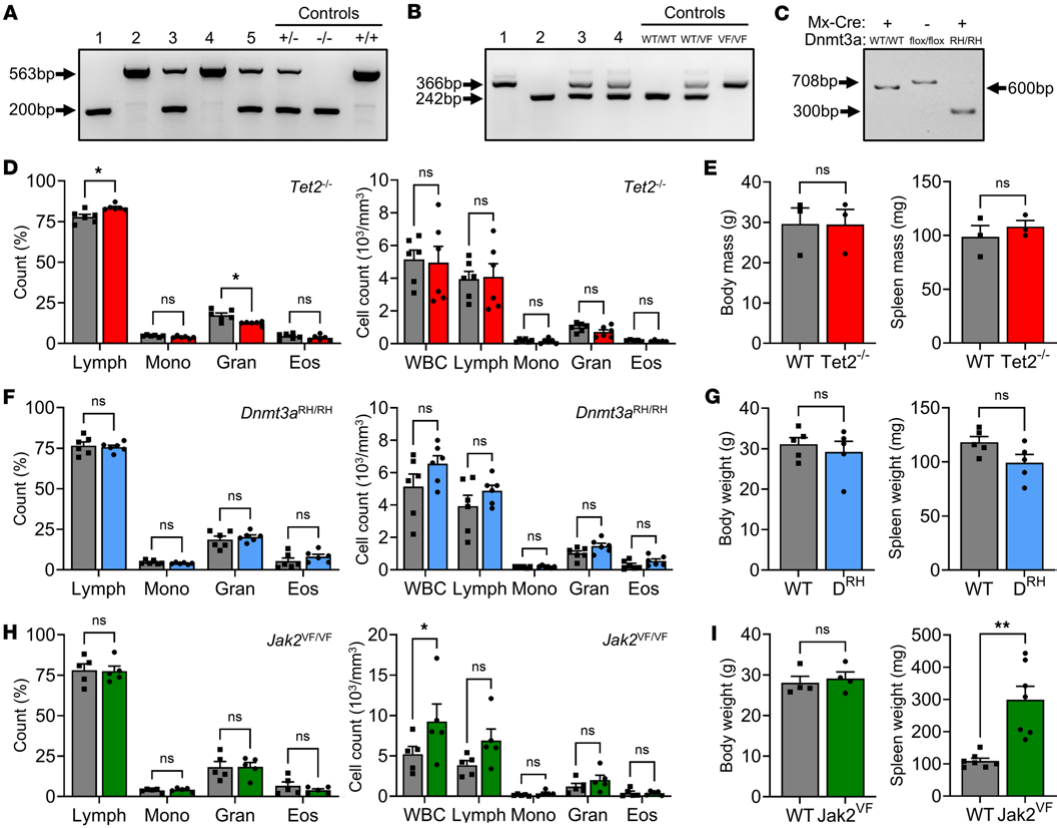
Validation of genotype and basic phenotyping of *Tet2*, *Jak2*^VF^, and *Dnmt3a*^RH^ mutant mice. (**A** and **B**) Separated PCR products identifying WT alleles (+) (563bp) and loss of exons 8–10 in *Tet2* mutant mice (–) (200 bp) (**A**), or WT alleles (WT) (242bp) and those with insertion of the *Jak2*^V617F^ mutation (VF) (366bp) (**B**) in samples 1–4/5. (**C**) Separated PCR products from mice all treated with Poly(I:C) identifying the WT allele (WT) (600 bp), the *Dnmt3a* floxed allele (flox/flox) (708 bp) and Poly(I:C)-induced Mx1-Cre–mediated recombination to the R882H mutant allele (RH) (300 bp). (**D**–**I**) Blood cell counts (**D**, **F**, and **H**) and body and spleen weight (**E**, **G**, and **I**) in WT (gray), *Tet2*^–/–^ (red), *Dnmt3a*^RH/RH^ (blue) and *Jak2*^VF/VF^ (green) mice under the steady state. Data represent mean ± SEM of *n* = 6/6 (**D** and **F**), 3/3 (**E**), 5/5 (**G** and **H**), 4–7 (**I**); *P ≤ 0.05, ** *P* ≤ 0.01 by unpaired *t* test.

DNTM3A, *TET2*, and *JAK2* are among the most frequently mutated genes in human CHIP. *Tet2*^–/–^ mice are KOs commonly used to model *TET2* LOF mutations (48), while *Jak2*^V617F^ (hereinafter known as *Jak2*^VF^) mice employ a knock-in in order to model the GOF point mutation found in myeloproliferative neoplasms (49). *Dnmt3a*^fl–R882H^ is a conditional knock-in model in which Cre-*loxP* recombination via Poly(I:C)-inducible *Mx1*-Cre generates the most common human R882H mutation (50). Genotyping of *Tet2*^–/–^ and *Jak2*^VF^ mice showed the correct band pattern and size (Figure 1, A and B), as expected from previous reports (48, 49), while Poly(I:C) administration to *Dnmt3a*^fl–R882H^/*Mx1*-Cre mice resulted in loss of the WT band and generation of the correct band for the R882H mutation (Figure 1C) (hereinafter known as *Dnmt3a*^RH^). Phenotyping of mutant mice showed no major difference in blood counts, body and spleen weight, Ly6C^Hi^ monocytes, or splenic CD4/8 T cells, Tregs, and germinal center B cells under the steady state in *Tet2*^–/–^ (Figure 1, D and E, and Supplemental Figure 1, A–G; supplemental material available online with this article; https://doi.org/10.1172/jci.insight.200334DS1) or *Dnmt3a*^RH/RH^ (Figure 1, F and G, and Supplemental Figure 2, A–G) mice. However, *Jak2*^VF/VF^ mice had markedly increased WBC and RBC counts, hemoglobin, and spleen weight (Figure 1, H and I, and Supplemental Figure 3, A–G) and, thus, show the expected phenotype demonstrated in previous reports (49).

### Loss of Tet2 increases Mɸ cytokine release and inflammasome activation.

**Figure 2:**
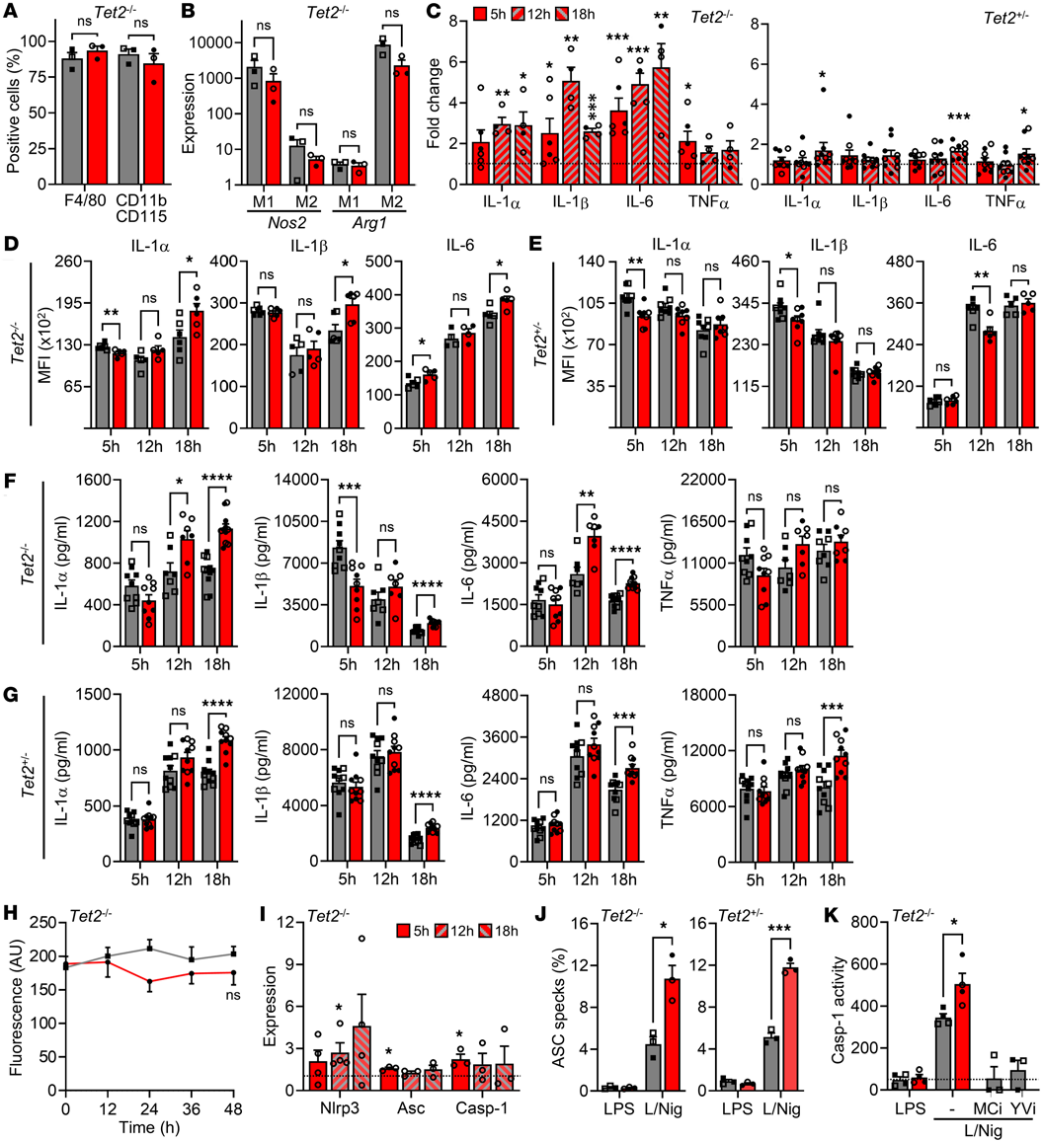
Loss of Tet2 increases macrophage cytokine release and inflammasome activation. (**A**–**K**) Phenotyping of WT (gray) and Tet2 mutant (red) BMDMs (Mɸs). (**A** and **B**) Flow cytometry for Mɸ lineage markers (**A**), or qPCR for polarisation markers after treatment with IFN-γ (M1) or IL-4/13 (M2) (**B**). (**C**) qPCR for cytokine transcript after 5, 12, or 18 hours of LPS in Tet2^–/–^ and Tet2^+/–^ Mɸs, relative to WT (dotted line). (**D** and **E**) Flow cytometry for intracellular cytokine staining after 5, 12, or 18 hours of LPS in WT, Tet2^–/–^ (**D**) and Tet2^+/–^ (**E**) Mɸs. (**F** and **G**) ELISA for cytokine release after 5, 12, or 18 hours of LPS, followed by 1 hour of nigericin, in WT, Tet2^–/–^ (**F**) or Tet2^+/–^ (**G**) Mɸs. (**H**) Alamar blue fluorescence for proliferation over 48 hours in WT and Tet2^–/–^ Mɸs. (**I**) qPCR for inflammasome components after 5, 12, or 18 hours of LPS in Tet2^–/–^ Mɸs, relative to WT (dotted line). (**J** and **K**) ASC specks (**J**) and caspase-1 (Casp-1) activity (**K**) after LPS, or LPS/Nigericin (L/Nig) treatment, with or without the inflammasome inhibitor MCC950 (MCi) or the casp-1 inhibitor YVAD-CHO (YVi). Open symbols = female. Data represent mean ± SEM of *n* = 3–4 (**A**, **B**, and **H**–**K**), 4–9 (**C**–**G**), as indicated; *P ≤ 0.05, **P ≤ 0.01, ***P ≤ 0.001, ****P ≤ 0.0001 by paired *t* test.

TET2 is a methylcytosine dioxygenase, with its deletion previously reported to increase atherosclerosis in mice via increased inflammatory cytokines (5, 16–18). To further investigate this, we differentiated BMDMs from littermate *Tet2*^+/+^ (WT), *Tet2*^+/–^, and *Tet2*^–/–^ mice. Both WT and *Tet2*^–/–^ genotypes generated mature Mɸs expressing F4/80 and CD11b/CD115 (Figure 2A), and both upregulated *Nos2* or *Arg1* when polarized in vitro to M1 (IFN-γ) or M2 (IL-4/13), respectively, but *Arg1* was lower in *Tet*^–/–^ Mɸs (Figure 2B). This suggests that *Tet2* loss does not generally affect Mɸ differentiation or polarization. Mɸs were treated with LPS for 5, 12, or 18 hours and transcripts for *Il1a*, *Il1b*, *Il6*, and *Tnf* were assessed. *Tet2*^–/–^ had substantially higher transcript levels (~2- to 5-fold) than WT Mɸs (Figure 2C), with greater differences at 12 and 18 hours and a smaller increase seen in *Tet2*^+/–^ Mɸs that was only statistically significant at 18 hours (Figure 2C). Despite increased transcript, intracellular cytokine proforms (using an assay validated with *Il1a*^–/–^ and *Il1b*^–/–^ Mɸs) (Supplemental Figure 4A) were only modestly elevated in *Tet2*^–/–^ Mɸs compared with WT (Figure 2D), with the difference again more prominent at 18 hours. In contrast, *Tet2*^+/–^ Mɸs contained the same or less cytokines than WT (Figure 2E). Importantly, secretion of mature cytokines after LPS or NLRP3 inflammasome activation showed a relatively consistent increase in IL-1α, IL-1β, and IL-6, in *Tet2*^–/–^ Mɸs at 12 or 18 hours (Figure 2F), but it showed reproducibly less IL-1β after 5 hours of LPS and no change in TNF-α at any time point. *Tet2*^+/–^ Mɸs again showed smaller increases restricted to 18 hours (Figure 2G). To test if increased cytokine secretion at later time points was due to more cells, we assessed Mɸ proliferation but found no difference between groups (Figure 2H). Finally, we explored inflammasome function and found modestly increased *Nlrp3*, *Pycard* (ASC), and *Casp1* expression (Figure 2I), we found a large increase in ASC specks in both *Tet2*^–/–^ and *Tet*^+/–^ Mɸs relative to WT (Figure 2J and Supplemental Figure 4B) and increased caspase-1 activity (Figure 2K), indicating increased NLRP3 inflammasome activation. Measured caspase-1 activity was induced by inflammasome activation and reduced to basal levels with the inflammasome inhibitor MCC950 or the caspase-1 inhibitor YVAD-CHO (Figure 2K), proving specificity. Finally, equivalent results were seen with both male and female Mɸs across all findings. Together, loss of *Tet2* function has disparate effects across cytokine expression and secretion, but production of most cytokines was elevated, with a larger effect seen with biallelic *Tet2* loss and a pronounced increase in NLRP3 inflammasome activation.

### Dnmt3a^RH^ mutation decreases Mɸ cytokine release and inflammasome activation.

**Figure 3:**
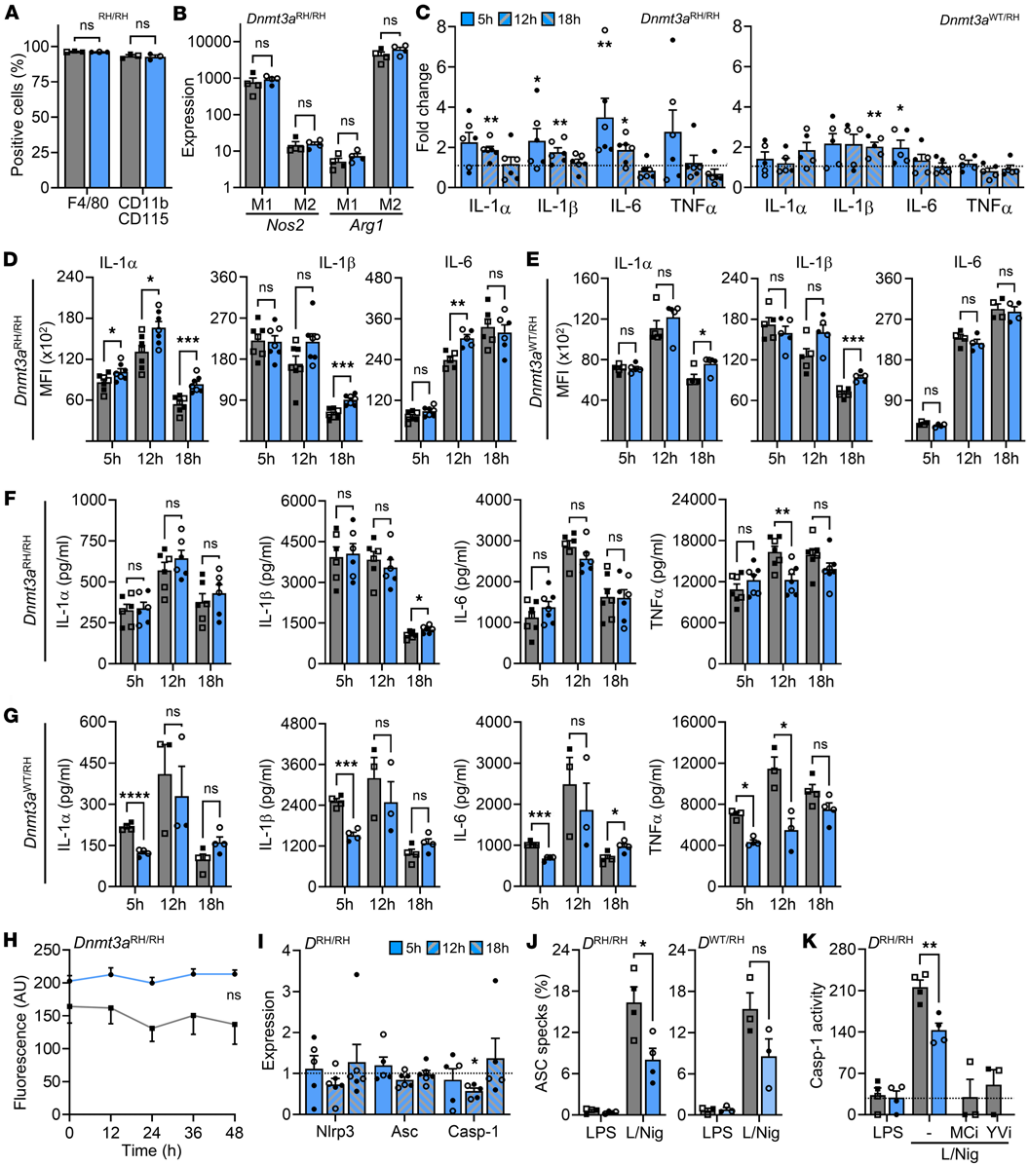
Dnmt3aRH mutation decreases macrophage cytokine release and inflammasome activation. (**A**–**K**) Phenotyping of WT (gray) and Dnmt3aRH mutant (blue) BMDMs (Mɸs). (**A** and **B**) Flow cytometry for Mɸs lineage markers (**A**) or qPCR for polarisation markers after treatment with IFN-γ (M1) or IL-4/13 (M2) (**B**). (**C**) qPCR for cytokine transcript after 5, 12, or 18 hours of LPS in *Dnmt3a^RH/RH^* and Dnmt3aWT/RH Mɸs, relative to WT (dotted line). (**D** and **E**) Flow cytometry for intracellular cytokine staining after 5, 12, or 18 hours of LPS in WT, *Dnmt3a^RH/RH^* (**D**) and Dnmt3aWT/RH (**E**) Mɸs. (**F** and **G**) ELISA for cytokine release after 5, 12, or 18 hours of LPS, followed by 1 hour of nigericin, in WT, *Dnmt3a^RH/RH^* (**F**) and Dnmt3aWT/RH (**G**) Mɸs. (**H**) Alamar blue fluorescence for proliferation of WT and *Dnmt3a^RH/RH^* Mɸs over 48 hours. (**I**) qPCR for inflammasome components after 5, 12 or 18 hours of LPS in *Dnmt3a^RH/RH^* (DRH/RH) Mɸs, relative to WT (dotted line). (**J** and **K**) ASC specks (**J**) and caspase-1 (Casp-1) activity (**K**) after LPS, or LPS/Nigericin (L/Nig) treatment, ±the inflammasome inhibitor MCC950 (MCi) or the casp-1 inhibitor YVAD-CHO (YVi). Open symbols = female. Data represent mean ±SEM of *n* = 3 (**A** and **H**), 3–7 (**B**–**G** and **I**–**K**), as indicated; *P ≤ 0.05, **P ≤ 0.01, ***P ≤ 0.001, ****P ≤ 0.0001 by paired *t* test.

DNMT3A is a methyltransferase whose actions oppose those of TET2, but LOF mutations in *Dnmt3a* have been reported to produce a similar phenotype as those in *Tet2* (19). We differentiated Mɸs from littermate *Dnmt3a*^WT/WT^ (WT), *Dnmt3a*^WT/RH^, and *Dnmt3a*^RH/RH^ mice and investigated responses. Both WT and *Dnmt3a*^RH/RH^ genotypes differentiated to mature Mɸs expressing F4/80 and CD11b/CD115 (Figure 3A), and both upregulated *Nos2* or *Arg1* when polarized to M1 or M2, respectively (Figure 3B), suggesting *Dnmt3a* mutation does not affect Mɸ differentiation or polarization. LPS-treated *Dnmt3a* mutant Mɸs had more *Il1a*, *Il1b*, and *Il6* transcript (~2- to 4-fold) than WT (Figure 3C), but a bigger difference was seen at earlier time points with no difference at 18 hours — the opposite of *Tet2* mutants (Figure 2C). Again, a smaller increase was seen in *Dnmt3a*^WT/RH^ Mɸs compared with *Dnmt3a*^RH/RH^ (Figure 3C). Intracellular cytokine proforms were only modestly elevated in *Dnmt3a*^RH/RH^ Mɸs compared with WT (Figure 3D), with less difference seen in *Dnmt3a*^WT/RH^ (Figure 3E). Secretion of mature cytokines after LPS or NLRP3 inflammasome activation showed no tangible difference between *Dnmt3a*^RH/RH^ and WT Mɸs (Figure 3F) but consistently less cytokine release from *Dnmt3a*^WT/RH^ Mɸs (Figure 3G). Again, WT and *Dnmt3a*^RH/RH^ Mɸs showed no difference in proliferation (Figure 3H), indicating that cell number did not alter cytokine level. Interestingly, modestly reduced expression of *Casp1* (Figure 3I), and a large decrease in ASC specks in both *Dnmt3a*^WT/RH^ and *Dnmt3a*^RH/RH^ cells was seen (Figure 3J and Supplemental Figure 4B), along with less caspase-1 activity (Figure 3K), indicating less NLRP3 inflammasome activation than in WT Mɸs. Again, similar results were observed between male and female Mɸs. Together, *Dnmt3a*^RH^ mutation also has disparate effects across cytokine expression and secretion, but despite increased intracellular level, the release of most mature cytokines was similar or decreased, with a larger effect seen with biallelic *Dnmt3a* mutation, and a marked effect on NLRP3 inflammasome activation in the opposite direction to that seen with *Tet2*.

### Jak2^VF^ mutation does not alter Mɸ cytokine release or inflammasome activation.

**Figure 4:**
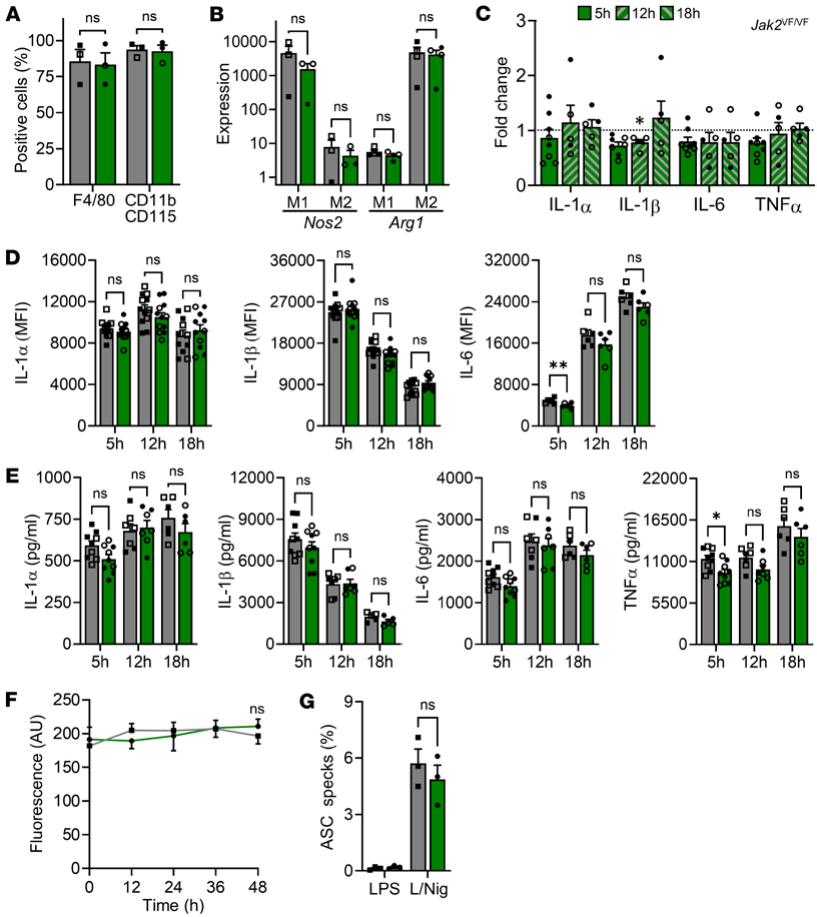
*Jak2*^VF^ mutation does not alter macrophage cytokine release or inflammasome activation. (**A**–**G**) Phenotyping of WT (gray) and *Jak2*^VF/VF^ (green) BMDMs (Mɸs). (**A** and **B**) Flow cytometry for Mɸ lineage markers (**A**) or qPCR for polarization markers after treatment with IFN-γ (M1) or IL-4/13 (M2) (**B**). (**C**) qPCR for cytokine transcript after 5, 12, or 18 hours of LPS in *Jak2*^VF/VF^ Mɸs, relative to WT (dotted line). (**D**) Flow cytometry for intracellular cytokine staining after 5, 12, or 18 hours of LPS in *Jak2*^VF/VF^ and WT Mɸs. (**E**) ELISA for cytokine release after 5, 12, or 18 hours of LPS, followed by 1 hour of nigericin in *Jak2*^VF/VF^ and WT Mɸs. (**F**) Alamar blue fluorescence for proliferation of WT and *Jak2*^VF/VF^ Mɸs over 48 hours. (**G**) ASC specks after LPS or LPS/Nigericin (L/Nig) treatment. Open symbols = female. Data represent mean ± SEM of *n* = 3 (**A**, **B**, **F**, and **G**), 5–12 (**C**–**E**), as indicated; *P ≤ 0.05 by paired *t* test.

JAK2 is a nonreceptor tyrosine kinase commonly mutated in myeloproliferative disorders, with previous reports showing GOF mutation to increase inflammatory cytokines (20). Mɸs differentiated from littermate *Jak2*^WT/WT^ (WT) and *Jak2*^VF/VF^ mice gave equivalent F4/80 and CD11b/CD115 expression (Figure 4A), and *Nos2* or *Arg1* expression after polarization to M1 or M2 (Figure 4B). However, in contrast to *Tet2* and *Dnmt3a* mutants, LPS-treated *Jak2*^VF/VF^ Mɸs had equivalent *Il1a*, *Il1b, Il6*, and *Tnf* transcript level as WT, with no difference across length of stimulation (Figure 4C). Similarly, intracellular cytokine proforms were comparable in WT and *Jak2*^VF/VF^ Mɸs (Figure 4D), while secretion of mature cytokines after LPS or NLRP3 inflammasome activation showed no tangible difference, with only a minor decrease (~0.1-fold) in TNF-α witnessed in *Jak2*^VF/VF^ compared with WT (Figure 4E). Again, no difference in Mɸ proliferation was seen between genotypes (Figure 4F), and in contrast to *Tet2* and *Dnmt3a*^RH^ mutants, no difference in ASC speck formation was seen (Figure 4G). Again, similar results were observed between male and female Mɸs. Together, *Jak2*^VF^ mutation did not alter Mɸ cytokine expression, release, or NLRP3 inflammasome activation, and given this lack of phenotype, *Jak2*^WT/VF^ Mɸs were not studied.

### Tet2 mutant alleles, but not Dnmt3a^RH^ or Jak2^VF^, drive clonal expansion in mouse models of CHIP.

**Figure 5:**
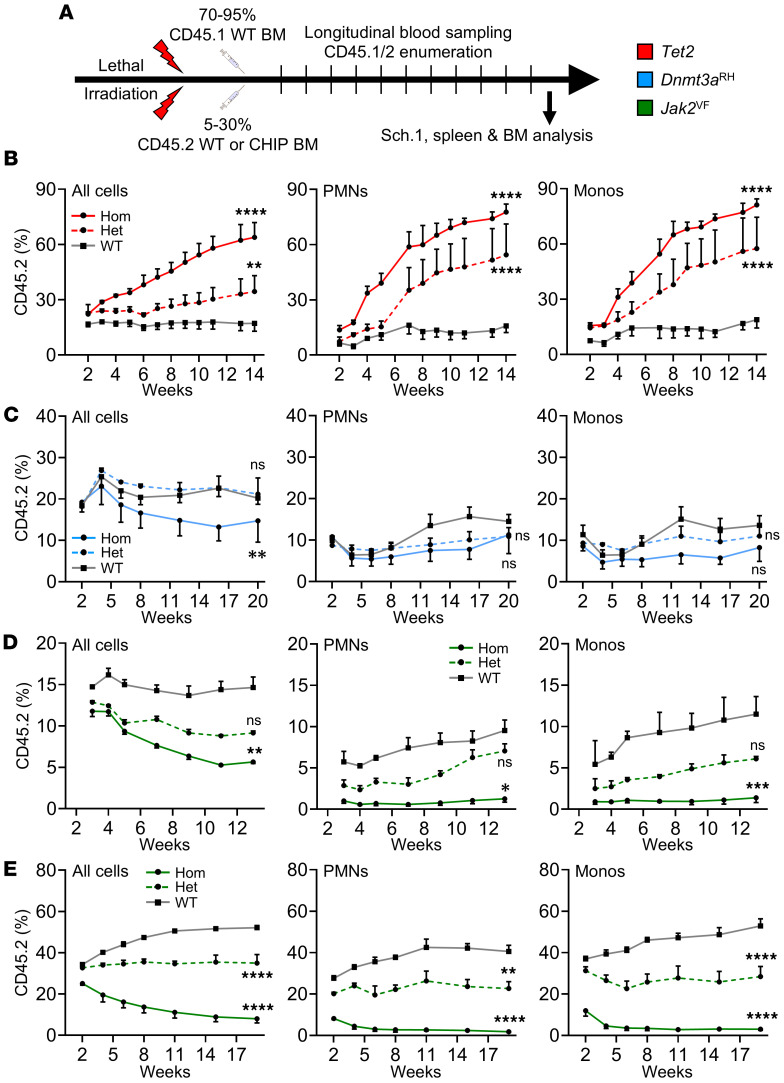
*Tet2* mutant cells clonally expand in mice, but *Dnmt3a*^RH^ and *Jak2*^VF^ cells are lost. (**A**) Experimental schematic showing irradiation, reconstitution with BM from WT and CHIP mutants, blood sampling, and euthanasia (Sch.1). (**B**–**E**) Longitudinal measurement of clonal size via CD45.2 in mice transplanted with 5% *Tet2* (red; female) (**B**), 10% *Dnmt3a*^RH^ (blue; male) (**C**), 5% *Jak2*^VF^ (green; male) (**D**), or 30% *Jak2*^VF^ (**E**) homozygous (Hom) or heterozygous (Het) mutant cells or the equivalent WT cells. CD45.2 level was measured in all circulating cells (All cells), neutrophils (PMNs), and monocytes (Monos). Weeks = time after BM transplant. Data represent mean ± SEM of *n* = 3/3/3 (**B**, **D**, and **E**), 4/4/4 (**C**); **P ≤ 0.01, ***P ≤ 0.001, ****P ≤ 0.0001 by generalized linear mixed models.

The fundamental characteristic of human CHIP is the progressive expansion of mutant clones over time. To model this, we generated mixed BM chimeras containing a minor proportion of CD45.2^+^ CHIP mutant cells and a major proportion of CD45.1^+^ WT cells, allowing highly tractable assessment of cell origin and lineage by flow cytometry (Figure 5A and Supplemental Figure 5). Mice transplanted with a ratio of 5% *Tet2*^–/–^ to 95% WT cells (compared with 5% CD45.2 WT to 95% CD45.1 WT cells) showed progressive expansion of *Tet2*^–/–^ CD45.2^+^ cells in the circulation over 14 weeks to a maximum level of ~60% (Figure 5B), and similar expansion specifically within neutrophils and monocytes was found (Figure 5B). *Tet2*^+/–^ CD45.2^+^ cells also clonally expanded across all linages examined but at a slower rate than *Tet2*^–/–^ (Figure 5B). Given the increased IL-1 from *Tet2*^–/–^ Mɸs (Figure 2, F and G) and known effect of IL-1 on hematopoiesis (35, 36), we tested if IL-1 drove clonal growth, but long-term IL-1 blockade with anakinra (IL-1RA) did not alter expansion in any lineage (Supplemental Figure 6, A–C). Next, we transplanted mice with a ratio of 10% *Dnmt3a*^RH/RH^ to 90% WT cells (versus 10% CD45.2 WT to 90% CD45.1 WT cells) and observed a progressive decrease in the proportion of *Dnmt3a*^RH/RH^ CD45.2^+^ cells in the circulation over 20 weeks (Figure 5C), as well as no expansion within neutrophils, monocytes (Figure 5C), B cells (Supplemental Figure 7A), or in BM or spleen at the end of the experiment (Supplemental Figure 7, B and C). *Dnmt3a*^WT/RH^ CD45.2^+^ cells also did not expand across any lineage examined but behaved more similarly to WT cells (Figure 5C and Supplemental Figure 7, A–C). Finally, we transplanted mice with a ratio of 5% *Jak2*^VF/VF^ to 95% WT cells (compared with 5% CD45.2 WT to 95% CD45.1 WT cells) and witnessed a gradual decrease in the fraction of *Jak2*^VF/VF^ CD45.2^+^ cells over 13 weeks across all lineages and locations examined (Figure 5D and Supplemental Figure 7, D and E). *Jak2*^WT/VF^ CD45.2^+^ cells also did not expand clonally (Figure 5D and Supplemental Figure 7, D and E). As a previous study identified known issues with adoptive transfer of *Jak2*^VF/VF^ cells (49), we repeated the experiment using a ratio of 30% *Jak2*^VF/VF^ to 70% WT, but *Jak2*^VF/VF^ cells were again progressively lost over 24 weeks, while *Jak2*^WT/VF^ cells remained at a relatively stable frequency (Figure 5E and Supplemental Figure 7, F–H). In contrast, WT CD45.2 cells showed a steady expansion over 24 weeks (Figure 5E), as previously reported (51), suggesting transplanted *Jak2*^WT/VF^ cells may still be at an engraftment and/or proliferative disadvantage. Importantly, failure to detect expansion with *Dnmt3a*^RH^ or *Jak2*^VF^ was not due to incorrect powering, as the observed change well exceeded the powered effect size (Supplemental Figure 8). Of note, the proportion of CD45.2^+^ cells in “all cells” after BM transplant is typically ~10% higher than in monocytes or neutrophils in all genotypes studied due to the well-known contribution of radioresistant T cells (52). Together, only *Tet2* mutant cells show clonal expansion akin to human CHIP after BM transplant in mice, while experiments using *Dnmt3a*^RH^ or *Jak2*^VF^ cells do not show this key feature.

### Systemic inflammation or atherogenesis do not accelerate or induce clonal expansion in most mouse models of CHIP.

**Figure 6:**
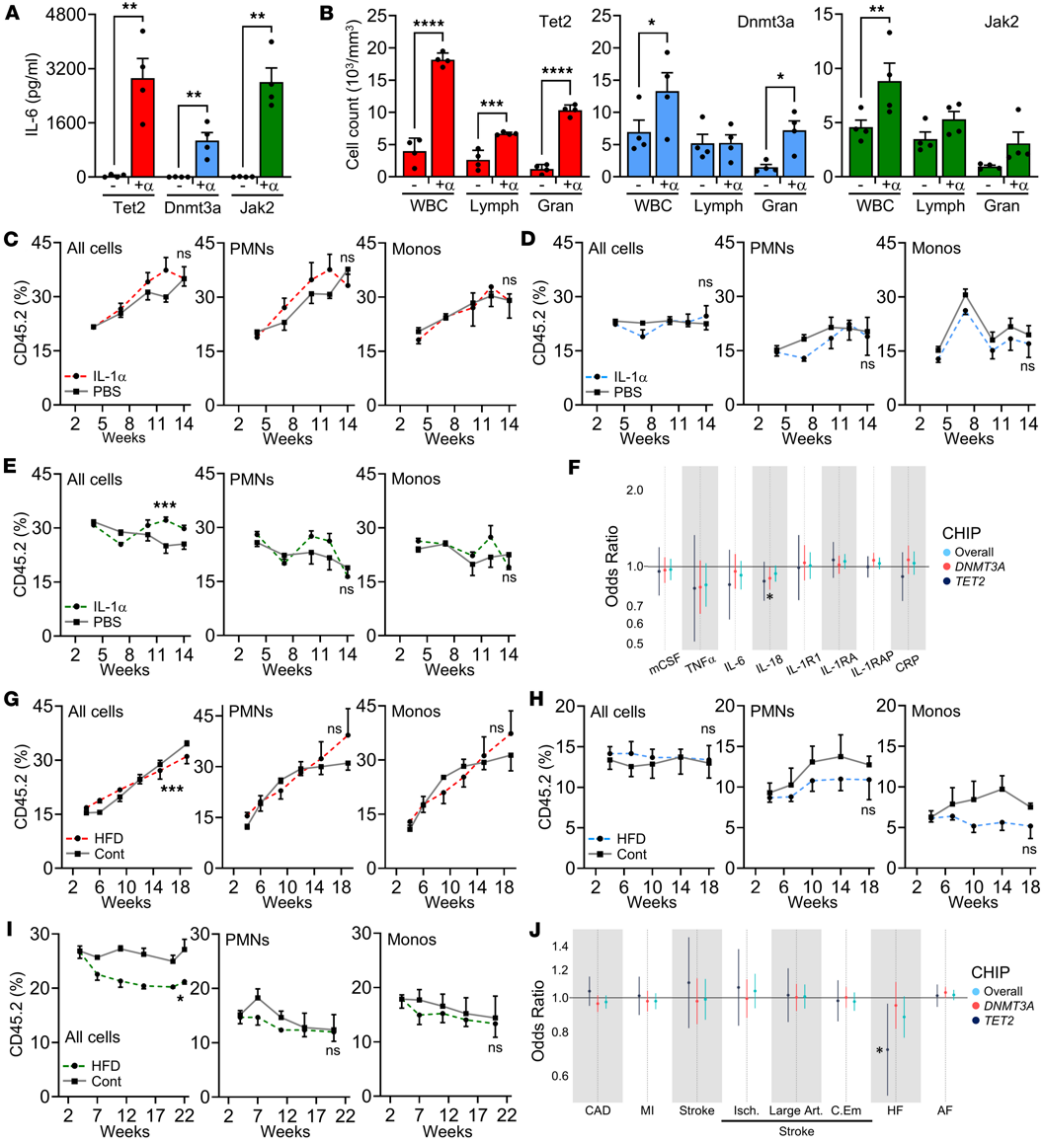
Systemic inflammation and atherogenesis do not accelerate clonal expansion in most mouse models of CHIP. (**A** and **B**) *Tet2^+/–^* (red), *Dnmt3a*^WT/RH^ (blue), and *Jak2*^WT/VF^ (green) mice were injected with IL-1α (+α) and serum IL-6 (**A**) and blood cells (**B**) quantified. WBC, white blood cells; Lymph, lymphocyte; Gran, granulocyte. (**C**–**E**) Longitudinal measurement of clonal expansion via CD45.2 in mice transplanted with 10% *Tet2^+/–^* (male) (**C**), 10% *Dnmt3a*^WT/RH^ (female) (**D**), or 30% *Jak2*^WT/VF^ (male) (**E**) cells, or equivalent WT, followed by treatment with PBS (gray) or IL-1α (color). Weeks = time after BM transplant. (**F**) Mendelian randomization (MR) analysis of genetically predicted cytokine levels on CHIP risk in humans within the UK BioBank. Point estimates represent odds ratio ± 95% CIs. (**G**–**I**) Longitudinal measurement of clonal expansion via CD45.2 in mice transplanted with 10% *Tet2^+/–^* (**G**), 10% *Dnmt3a*^WT/RH^ (**H**), or 30% *Jak2*^WT/VF^ (**I**) cells (all female), or equivalent WT cells, followed by feeding a normal chow (Cont; gray) or HFD (color). (**J**) MR analysis of genetically predicted cardiovascular disease on CHIP risk in humans within the UK BioBank. CAD, coronary artery disease; MI, myocardial infarction; isch, ischemic; art, artery; C.Em, cardioembolic; HF, heart failure; AF, atrial fibrillation. Data represent mean ±SEM of *n* = 4/4 (**A**–**E**, and **H**), 3/3 (**G**), 4/3 (**I**); *P ≤ 0.05, **P ≤ 0.01, ***P ≤ 0.001, ****P ≤ 0.0001 by unpaired *t* test (**A** and **B**) or generalized linear mixed models (**C**–**E** and **G**–**I**).

Inflammatory cytokines (e.g., IL-1) drive accelerated hematopoiesis (35, 36), while atherosclerosis generates systemic inflammation and leukocytosis (44). Thus, we postulated that inflammation and/or atherogenesis might favor clonal expansion. Long-term treatment of *Tet2^+/–^*, *Dnmt3a*^WT/RH^, and *Jak2*^WT/VF^ mice with IL-1α raised circulating IL-6 (Figure 6A) and induced leukocytosis (Figure 6B) and splenomegaly (Supplemental Figure 9A), all supporting induction of systemic inflammation. Mice were transplanted with ratios of 10% *Tet2*^+/–^ to 90% WT cells, 10% *Dnmt3a*^WT/RH^ to 90% WT cells, or 30% *Jak2*^WT/VF^ to 70% WT, treated with or without IL-1α and clonal expansion measured. We specifically used monoallelic mutants, as *Tet2*^–/–^ cells already expanded very rapidly (Figure 5B), while *Dnmt3a*^RH/RH^ and *Jak2*^VF/VF^ cells were progressively lost (Figure 5, C–E). Again, CD45.2^+^ *Tet2*^+/–^ cells expanded, but chronic IL-1α did not alter clone growth in any lineage in the circulation, BM, or spleen examined (Figure 6C and Supplemental Figure 9, B–D). As before, *Dnmt3a*^WT/RH^ and *Jak2*^WT/VF^ cell lineages remained stable, with IL-1α treatment not altering this (Figure 6, D and E, and Supplemental Figure 9, E–J), other than a small stabilization of *Jak2*^WT/VF^ seen with IL-1α in “all cells” and B cells. Supporting these findings, MR analyses of human data in the UK BioBank found no evidence for causality between genetically predicted levels of multiple circulating cytokines and increased risk of overall CHIP, *DNMT3A* CHIP, or *TET2* CHIP (Figure 6F and Supplemental Table 1).

To investigate if atherogenesis affects clonal expansion, we generated BM chimeras with mutant cells but used *Ldlr*^–/–^ mice as recipients to enable hyperlipidemia. Feeding mice a HFD resulted in elevated lipids compared with chow (Supplemental Figure 10A), leukocytosis (Supplemental Figure 10B), and splenomegaly (Supplemental Figure 10C), as expected. However, HFD feeding did not increase expansion of *Tet2^+/–^* cells (Figure 6G and Supplemental Figure 10, D–F) or induce expansion of *Dnmt3a*^WT/RH^ (Figure 6H and Supplemental Figure 10, G–I) or *Jak2*^WT/VF^ cells (Figure 6I and Supplemental Figure 10, J and K), other than a small (but statistically significant) decrease in *Tet2^+/–^* “all cells” and B cells, and *Jak2*^WT/VF^ “all cells.” Since previous work showed increased expansion of *Tet2^–/–^* cells with HFD (32), we tested biallelic *Tet2^–/–^* or *Dnmt3a*^RH/RH^ donor cells. This showed a small (but statistically significant) increase in clonal expansion on HFD with *Tet2^–/–^* (Supplemental Figure 11, A–C) but not *Dnmt3a*^RH/RH^ (Supplemental Figure 11, D–F) cells. Finally, MR analyses of genetically predicted risk of CVD (including atherosclerosis) were not causally associated with increased risk of CHIP overall, *DNMT3A* CHIP, or *TET2* CHIP (Figure 6J and Supplemental Tables 2 and 3). These data indicate that, in monoallelic mouse models of CHIP with *Tet2*^+/–^, *Dnmt3a*^WT/RH^, or *Jak2*^WT/VF^ cells, neither IL-1α–driven systemic inflammation nor HFD-induced atherogenesis alter clonal dynamics, and MR studies with human data support these conclusions. Together, these data suggest that the relationship between CHIP and CVD in humans and mouse models is unlikely to be bidirectional or due to reverse causation, in keeping with recent human studies (12).

### CHIP models have mutation and sex-dependent effects on atherosclerosis in mice.

**Figure 7:**
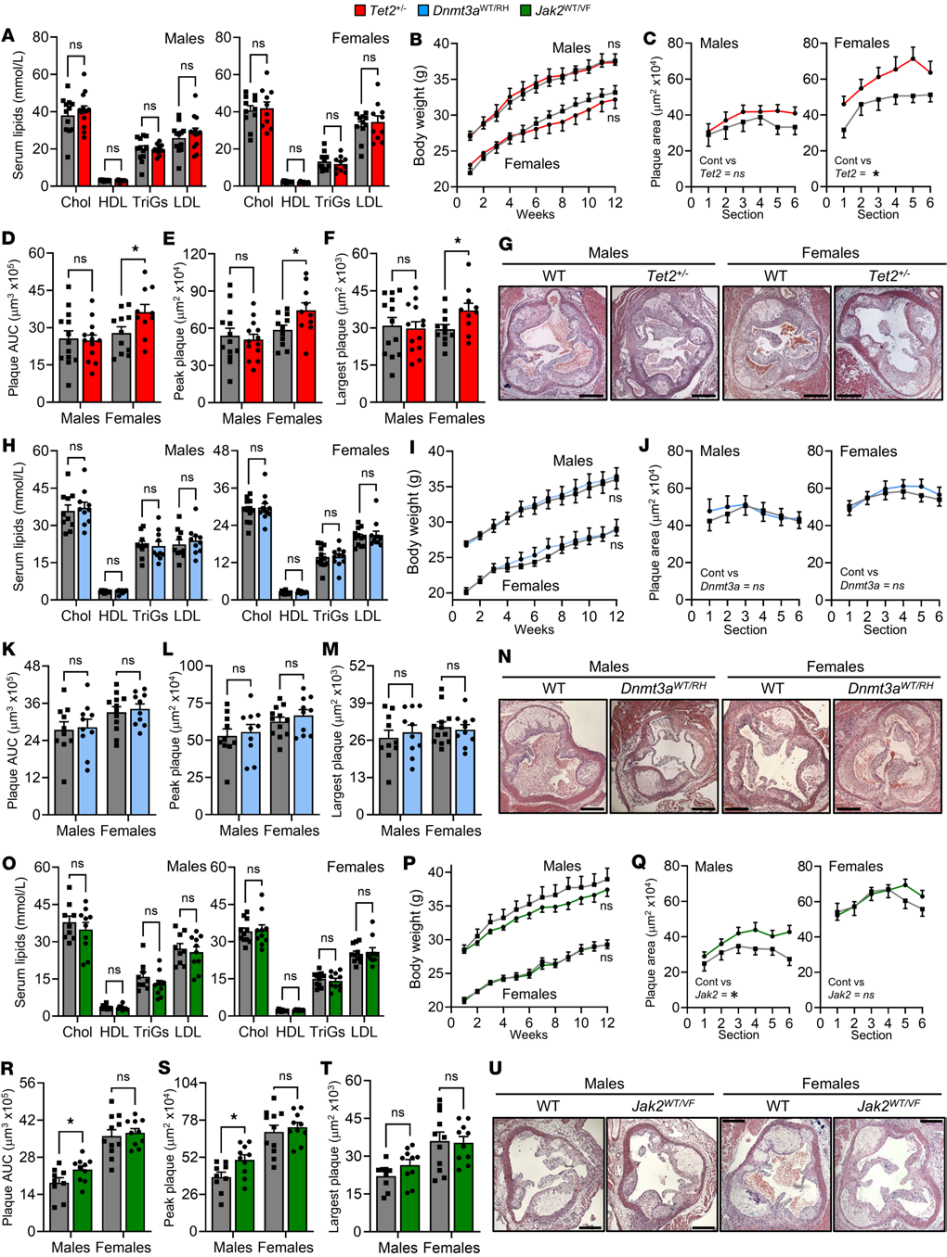
CHIP models have mutation and sex-dependent effects on atherosclerosis in mice. (**A**–**U**) Assessment of atherosclerosis in male and female mice transplanted with 10% *Tet2*^+/–^ (red) (**A**–**G**), 10% *Dnmt3a*^WT/RH^ (blue) (**H**–**N**), or 30% *Jak2*^WT/VF^ (green) (**O**–**U**) mutant cells, or equivalent WT cells (gray), followed by HFD feeding for 12 weeks. Mice were assessed for serum lipids at 6 weeks (**A**, **H**, and **O**) and longitudinal body weight (**B**, **I**, and **P**). Aortic root plaques were serial sectioned, quantified (**C**, **J**, and **Q**) and analyzed for AUC (**D**, **K**, and **R**), peak plaque area (**E**, **L**, and **S**), and single largest plaque (**F**, **M**, and **T**), along with representative images (**G**, **N**, and **U**). Data represent mean ± SEM of *n* = 13/13 (male) and 11/10 (female) (**A**–**G**), 10/10 (male) and 12/10 (female) (**H**–**N**), *n* = 9/10 (male) and 11/10 (female) (**O**–**U**); *P ≤ 0.05; by unpaired *t* test (**A**, **D**–**F**, **H**, **K**–**M**, **O**, and **R**–**T**) or generalized linear mixed models (**B**, **C**, **I**, **J**, **P**, and **Q**). Scale bars: 500 μm.

Human somatic mutations in CHIP are monoallelic; thus, it is important to understand if mouse models of CHIP using *Tet2^+/–^*, *Dnmt3a*^WT/RH^, and *Jak2*^WT/VF^ effects atherosclerosis. We generated BM chimeras with CHIP mutant or WT donor cells in *Ldlr*^–/–^ recipients, utilizing both male and female cohorts. Feeding a HFD elevated serum lipids and body weight, but with no difference between WT or *Tet2*^+/–^ mutants (Figure 7, A and B). After 12 weeks of HFD, there was a marked increase in female *Tet2*^+/–^ CHIP aortic root plaque size analyzed by serial sectioning (Figure 7C), AUC (Figure 7D), peak aortic root plaque (Figure 7E), or single largest plaque (Figure 7, F and G), along with smaller fibrous caps (Supplemental Figure 12A), compared with WT non-CHIP. However, male *Tet2*^+/–^ mice showed no differences in plaque between groups (Figure 7, C–G). Although *Dnmt3a*^RH^ and *Jak2*^VF^ mutant cells did not clonally expand, heterozygous cell populations remained more stable (Figure 5, C–E), and thus, we also investigated atherosclerosis in these systems. There was no difference in *Dnmt3a*^WT/RH^ or WT lipids or body weight, nor difference in aortic root plaque AUC, peak aortic root plaque, or single largest plaque between groups in males or females (Figure 7, H–N). However, a small increase in necrotic core size was seen in male *Dnmt3a*^WT/RH^ CHIP (Supplemental Figure 12B). Interestingly, and despite no change in Mɸ cytokines (Figure 4), body weights, or lipids (Figure 7, O and P), *Jak2*^WT/VF^ plaques were larger than WT in males but not females (Figure 7, Q and U) (with no change in other plaque parameters) (Supplemental Figure 12C), perhaps suggesting Mɸ cytokine and/or clonal expansion–independent effects of transplanted *Jak2*^WT/VF^ cells. Together, this shows that monoallelic CHIP-driver mutations increase atherosclerosis in a mutation- and sex-dependent manner.

## Discussion

As we age, somatic mutations accumulate in known leukemia driver genes, and although single mutations usually remain asymptomatic, they may increase risk of multiple diseases. CVDs are the single largest death by cause and occur with increasing prevalence on aging. Indeed, recent studies report a strong association between CHIP and CVD in humans, with preclinical data proposing increased inflammation from CHIP mutant immune cells to accelerate and worsen atherosclerosis. Given the well-known, trial-proven role of inflammation in vascular disease (53), the suggestion that CHIP encompassing a range of mutations could converge on inflammation as a common effector of increased CV risk offers an attractive proposition. However, how LOF mutations in genes with opposing functions could both increase IL-1β was unexplained, not all studies support the link between CHIP and CVD, and directionality and causality between some CHIP mutations and CVD remains unresolved.

We show *Tet2* and *Dnmt3a*^RH^ LOF mutations to have opposite effects on Mɸ cytokine release, with increased cytokines and NLRP3 inflammasome activation with *Tet2* and decreased or the same with *Dnmt3a*^RH^. However, *Jak2*^VF^ GOF mutant Mɸ cytokine expression was equivalent to WT. Clonal growth in mice occurred with *Tet2* mutant cells, but not *Dnmt3a*^RH^ or *Jak2*^VF^. IL-1–driven systemic inflammation did not affect clonal expansion, while HFD-induced atherosclerosis only increased expansion of *Tet2*^–/–^ cells but not monoallelic mutants. Moreover, human MR studies showed no effect of genetically predicted serum cytokines or CVD on CHIP. Atherosclerosis increased in female mice with *Tet2*^+/–^ and male with *Jak2*^WT/VF^ cells, but not mice with *Dnmt3a*^WT/RH^. Together, CHIP mutations have disparate effects on Mɸ cytokines, *Dnmt3a*^RH^ and *Jak2*^VF^ cells do not clonally expand in murine CHIP models, and the effect of CHIP on atherogenesis is mutation and sex dependent.

Several studies report increased IL-1β release from *Tet2*, *Dnmt3a*, and *Jak2* mutant Mɸs (16, 20, 21), while others show no difference (5, 19, 24, 29–31). As CHIP mutations occur in genes with disparate function, convergence on IL-1β has remained unexplained. We used littermate WT, heterozygous and homozygous mice, multiple independent Mɸ differentiations, and we analyzed cytokine transcript, intracellular proform and secreted mature forms at multiple time points to robustly investigate the effect of CHIP mutations on Mɸs. *Tet2* mutant Mɸs tended to express more cytokine than WT after longer stimulations, and with a larger effect upon biallelic loss. Ultimately, more IL-1α/β, IL-6, and TNF-α was secreted by *Tet2* mutant cells after 18 hours of LPS, but less difference was seen in intracellular proforms of these cytokines. Indeed, the largest effect of *Tet2* loss was an approximate doubling in ASC speck formation and caspase-1 activity (markers of active inflammasomes), suggesting this may be the cause of increased IL-1. In contrast, *Dnmt3a*^RH^ Mɸs tended to release less or the same amount of cytokine as WT and showed about half the ASC specks and caspase-1 activity as WT. This is particularly interesting, as DNMT3A and TET2 have opposite functions on methylation; thus, an opposite effect on inflammasome activation is perhaps biochemically more rational. Thus, although *Dnmt3a* LOF mutations are reported to increase IL-1β transcript and intracellular proform (19) (which we also see), this is likely counterbalanced by reduced NLRP3 inflammasome function. In contrast, *Jak2*^VF^ Mɸs showed only a small decrease in intracellular IL-6 and secreted TNF-α at 5 hours in *Jak2*^VF/VF^ cells, which is in keeping with some studies (30, 31) but discordant with a reported 5-fold more IL-1β after AIM2 or NLRP3 activation (20). Our extensive analysis reveals that, although differences in cytokine level between CHIP and WT Mɸs can be identified at individual time points, stages of expression, or genotypes, a broad consensus exists for more cytokine release from *Tet2* and less or no difference with *Dnmt3a*^RH^ and *Jak2*^VF^ Mɸs.

The fundamental characteristic of human CHIP is clonal expansion over time. *Tet2*^–/–^ mutant cells expanded robustly, but at a slower rate with monoallelic mutations, as occurs in human CHIP. In contrast, both *Dnmt3a*^RH/RH^ and *Jak2*^VF/VF^ cells were lost from the population over time, even with initial transplant of 30% *Jak2*^VF/VF^ cells. *Dntm3a*^WT/RH^ and *Jak2*^WT/VF^ cells persisted for longer but still did not expand in any lineage or tissue examined across multiple experiments. Indeed, the original generation and description of *Jak2*^VF^ mice reported this failure to repopulate (49), while studies using *Dnmt3a*^–/–^ cells did not see expansion without infection (54) or aging (55), or they only measured chimerism at a single time point (19), which cannot infer clonal expansion. Pragmatically, *Dnmt3a* and *Jak2* mutants clearly expand in human CHIP, perhaps suggesting a need for a “second hit” (e.g., aging, infection), a nonhematopoietic cell intrinsic role of *Dnmt3a* and *Jak2* mutants that does not occur in BM chimeric mice (e.g., in niche or stromal cells), and/or slow expansion in the HSC compartment that is not seen in the circulation over the times we examined. Indeed, *Dnmt3a*^–/–^ BM cells show expansion at 8 months after mycobacterial infection (54), which could be construed as a second hit. Overall, our data show that, of the CHIP drivers tested, only *Tet2* mutants expand in mice, and thus, adoptive transfer of *Dnmt3a*^RH^ or *Jak2*^VF^ BM does not model this key characteristic of human CHIP.

Although CVD risk associates with presence of CHIP — with the premise CHIP drives CVD — it is also possible that CVD drives CHIP or that the relationship is bidirectional. Indeed, several MR analyses found no evidence of causality for CHIP driving atherosclerosis (7, 14), raising the possibility of reverse causation. This is the first study to our knowledge to robustly examine if experimental atherosclerosis could drive CHIP across a range of driver mutations concomitantly. In addition, IL-1–driven inflammation did not cause expansion of *Tet2*^+/–^, *Dnmt3a*^WT/RH^, *Jak2*^WT/VF^, or WT cells, and IL-1 blockade did not alter *Tet2*^–/–^ expansion, despite increased IL-1 from *Tet2* mutant cells and prior evidence IL-1 drives *Tet2*^+/–^ expansion upon aging (56). Our MR analysis of genetically predicted cytokine level also showed no evidence of association with CHIP risk. Similarly, atherogenesis/hyperlipidemia did not promote clonal growth in any heterozygous mutant genotype, but caused a small expansion of *Tet2*^–/–^ cells (as previously reported) (32), and our MR analysis showed no evidence for increased CHIP risk in humans genetically predisposed to CVD, which aligns with recent longitudinal human studies (12, 33). Together, these observations suggest that an inflammatory and/or atherogenic environment do not accelerate CHIP in mice or humans, and that other factors may be needed to enable any promoting effect of the local BM milieu. As previous work shows exogenous IL-1 or NLRP3 inhibition to not alter expansion of *Tet2* or *Dnmt3a* mutant clones (16, 54), our findings suggest that targeting the inflammasome/IL-1β axis is unlikely to stop CHIP expansion but could mitigate inflammatory sequelae associated with *Tet2* LOF mutations. This consideration has important implications for ongoing trials currently assessing NLRP3 inflammasome inhibition in CHIP-associated diseases.

CHIP associates with increased risk of multiple CVDs, with most research focusing on atherosclerosis. To replicate human CHIP as closely as possible, we investigated atherogenesis with monoallelic mutations transplanted at pathophysiologically relevant frequencies in both male and female cohorts, and we studied 3 CHIP mutations concomitantly. We found no effect of *Dnmt3a*^WT/RH^ cell transplant on atherosclerosis in either sex, which supports previous findings with *Dnmt3a*^+/–^ mutants (19), as well as several large epidemiological studies that did not find an association of *DNMT3A* CHIP and CVD (7, 14, 57). However, we find *Jak2*^WT/VF^ cells to increase atherogenesis in males only, which conflicts with previous findings in female *Jak2*^VF^ mice (20, 21). These differences could be due to alternative genetics (e.g., Cre/Lox), local environment factors, and/or study of 1 sex only. Lastly, we found increased plaque size in females (but not males) with *Tet2*^+/–^ cells, supporting prior work conducted similarly studying females only (16). In addition, male (18) and female (17) *Tet2*^–/–^ CHIP mice show more atherosclerosis. However, despite strong epidemiologic data for *TET2* CHIP driving CVD (4–7), several conflicting studies exist (14, 25–28, 57). We do not currently know what causes sex-dependent effects of *Tet2*^+/–^ or *Jak2*^VF^ cells on atherosclerosis, but it does not appear to be sexual dimorphic cytokine production between mutations. Regardless, our work emphasizes the importance of considering sex as a biological variable in both experimental and epidemiologic research, with sex-specific human analyses warranted but yet to be published. Interestingly, women’s CVD risk increases sharply with age to match that of men, with a similar trend to CHIP prevalence. Given our findings, it is possible that a female-specific effect of *TET2* CHIP contributes to this. We found murine atherosclerosis is increased in a mutation- and sex-dependent way after transplant of heterozygous CHIP mutant cells, but the relevance to humans and basis for sex differences is currently unclear.

With all experimental and model systems come limitations. A major limitation of the most common approach to model CHIP in mice is the need for irradiation and adoptive transfer of mutant cells. In human CHIP, somatic mutations occur in situ within a given niche, with subsequent selection based on the fitness conferred. In contrast, myeloablative lethal irradiation generates a calamitous milieu of cell death, necrosis, and inflammation, into which the new hematopoietic system must engraft and repopulate the BM, leading to replicative stress and potential exhaustion. Thus, mutations driving CHIP in humans may not necessarily engraft and drive clonal expansion in chimeric mice. Similarly, the effect of inflammation or atherogenesis on transplanted CHIP mutant cells may differ from the effect on human CHIP emerging in situ or the effect of aging. Also, the effect of human CHIP mutations on inflammatory responses, and at what variant allele frequency any effect would be biologically meaningful over inflammation generated from WT cells, is unknown. For example, if a given CHIP mutation doubled cytokine release and clones were present at 10%, this would only increase overall cytokine level by 10%, which is unlikely to make a biological difference. Although we assessed clonal expansion in blood to give longitudinal data, along with BM and spleen chimerism at euthanasia, we cannot exclude specific expansion in the HSC/progenitor population. Finally, different effectors can alter atherogenesis at different time points, and thus, CHIP mutants could have temporal effects that are not modeled in our studies.

In conclusion, these results demonstrate divergent findings with common CHIP models, supporting the consideration that CHIP-driven disease pathogenesis differs for each mutation and that translating findings from mice to humans requires considerable caution. Ultimately, the relationship between different types of CHIP and nonhematological diseases, including CVD, and the influence of sex on this warrants careful evaluation.

## Methods

### Sex as a biological variable.

Sex as a biological variable was considered throughout, with data points stratified by sex, separate cohorts of both male and female mice compared, and both sexes used in MR analyses.

### Animal protocols.

All mice were on a C57BL/6J background and originated from: *Tet2*^–/–^ (Jax #023359), *Dnmt3a*^flR882H^/*Mx1*-Cre (58), *Jak2*^V617F^ (Li & Green, Cambridge) (49), CD45.1 (Jax #002014), *Ldlr*^–/–^ (Jax #002207), *Il1a*, *Il1b* (originally Horai) (59), and C57BL/6 (Charles River). Sex-matched littermates were used for all comparisons within CHIP mutant mice, which were born at the expected frequencies with no gross phenotype. Specifically, *Tet2*^+/–^ and *Tet2*^–/–^ were compared with *Tet2*^+/+^; *Dnmt3a*^WT/fl–R882H^/*Mx1*-Cre^+^ and *Dnmt3a*^fl–R882H/fl–R882H^/*Mx1*-Cre^+^ were compared with *Dnmt3a*^fl–R882H^; and *Jak2*^WT/V617F^ and *Jak2*^V617F/V617F^ were compared with *Jak2*^WT/WT^. Mice were genotyped by PCR with the standard Jax primers and protocol, except *Jak2*^VF/VF^ that followed established protocols (49). *Dnmt3a*^RH/RH^ genotyping used: (5′–3′) GAGAC GTCTGTGTAGTGGACG and CCTGCACTTTTCTGTAGCTGG to detect WT (600 bp) and floxed (708 bp); and CCTGCACTTTTCTGTAGCTGG and GGGCAAGAACATAAAGTGACC to detect the recombined mutant allele (300 bp) with 57°C annealing. Mice were maintained on a 12-hour light/dark cycle and water, normal chow (#105, SAFE) or Western HFD (#829100; SDS) were available ad libitum. To induce *Mx1*-Cre, 5 IP injections of Poly(I:C) (2 mg; PBS) was given over 10 days, with all groups receiving Poly(I:C). BM chimeras were generated by standard methods. Briefly, CD45.2 mice were split dose irradiated (5.5 gray, 4 hours apart) and 1 × 10^7^ total BM cells from age and sex-matched *Tet2*, *Jak2*^VF^ (6–8 weeks old), *Dnmt3a*^RH^ (12–14 weeks old), or WT CD45.2 or WT CD45.1 mice injected via the tail vein, within 2 hours of final irradiation and left to reconstitute. Microbleeds were made via the pedal vein into EDTA, and full blood counts used a scil Vet ABC+ (Horiba). I.p. injections of IL-1α (20 ng/g; PBS; Peprotech) occurred thrice weekly for 9 weeks. Anakinra (Sobi) was delivered by minipump (Alzet) (37.5 μg/h; Sobi) for ~32 days. For experimental atherosclerosis *Ldlr*^–/–^ recipient mice were fed a HFD from 6 weeks old for 12 weeks, and serum lipids profiled at 6 weeks (Siemens Dimension RXL).

### Cell culture.

BM was flushed from femurs and tibias of mice (6–8 weeks old), washed, and cultured in RPMI 1640 supplemented with 2 mM L-glutamine, 100 U/mL penicillin, 10 μg/mL streptomycin, 50 μM β-mercaptoethanol and 10% FCS, with 15% L929 (ECACC) conditioned media during mBMDM differentiation. Where indicated, cells were treated with: LPS (1 μg/mL; time as indicated) and then with nigericin (15 μM; 1 hour; Invivogen) for inflammasome activation, with MCC950 (20 μM; Invivogen) for inhibition; with IFN-γ, IL-4, IL-13 (all 20 ng/mL; 18 hours; Peprotech) for Mɸ polarization; with brefeldin A (1x; Invitrogen) for intracellular IL-6 staining; or with EtOH (for nigericin) or DMSO (for MCC950) vehicle controls. Cell proliferation was assessed with Alamar blue (1x; 4 hours; Invitrogen), taking a baseline reading 12 hours after plating and then measurement every 12 hours. Caspase-1 activity was measured with Caspase-Glo (Promega) and specificity validated with MCC950 and YVAD-CHO (1 μM; Promega) as shown (Figure 2K and Figure 3K).

### Flow cytometry.

Differentiated Mɸs were incubated (30 minutes; room temperature [RT]) with anti-F4/80 (BM8; 1:10; BioLegend), anti-CD11b (M1/70; 1:800; BioLegend), and anti-CD115 (AFS98; 1:100; Invitrogen). For circulating neutrophils and monocytes, whole blood (EDTA) was Fc blocked (1:100; 10 minutes; RT; BioLegend) and incubated (30 minutes; RT) with anti-Ly6G (1AA8; 1:80; BioLegend), anti-CD115, and anti-Ly6C (7/4; 1:400; Bio-Rad), before RBC lysis. For clonal growth, whole blood (EDTA), flushed BM or dissociated spleen was Fc blocked, incubated (30 minutes; RT) with anti-CD45.1 (A20; 1:50), anti-CD45.2 (104; 1:80), anti-CD115, anti-Ly6G, anti B220 (RA3-6B2), or anti-CD3 (17A2) (all 1:100; all BioLegend), before RBC lysis (eBioscience). Intracellular cytokines were assessed with Fixation and Intracellular Staining Permeabilization Wash Buffer (BioLegend), using Fc block, anti-IL-1α (ALF-161; 1:100; BioLegend), anti-IL-1β (NJTEN3; 1:200; eBioscience) (validated as shown) (Supplemental Figure 4A), and anti-IL-6 (MP5-20F3; 1:200; BioLegend). Spleens were sieved (70 μm), before washing (PBS; 350 g, 5 minutes), re-sieving (40 μm), RBC lysis, washing, and resuspension in FACS buffer (1% BSA, 0.05% NaN3, in PBS). Cells in FACS buffer were Fc blocked before staining for T cell activation with anti-CD4 (RM4-5; 1:800; eBioscience), anti-CD8 (53-6.7; 1:100), anti-CD62L (MEL-14; 1:80), and anti-CD44 (IM7; 1:400; all BioLegend; 20 minutes, RT); for Tregs with anti-CD4 and anti-CD25 (PC61; 1:80; BioLegend; 20 minutes, RT), before washing, fixation, permeabilization (FOXP3 Fix/Perm; BioLegend), and then with anti-Foxp3 (FJK-16s; 1:20; BioLegend; 30 minutes, RT); and for B cells with anti-B220. All samples were washed, resuspended in FACS buffer, and analyzed by flow cytometry (Accuri C6). Examples of key flow cytometry plots and gating strategies are presented (Supplemental Figure 5).

### qPCR and ELISA.

mRNA was extracted using the RNeasy Mini kit (Qiagen), as per instructions, and quantity and purity accessed by spectroscopy (NanoDrop 1000). RNA was reverse transcribed to cDNA using Reverse Transcription System (Promega), as per instructions. *Il1a* (Mm99999060_m1), *Il1b* (Mm00434228_m1), *Il6* (Mm00446190_m1), *Tnf* (Mm00443258_m1), *Arg1* (Mm00475988_m1), *Nos2* (Mm00440502_m1), and *Gusb* (reference gene) (Mm01197698_m1) were quantified using TaqMan primers, with reactions of cDNA (1 μL), TaqMan probes (1 μL), reaction buffer (2 μL; 10x), MgCl_2_ (1.6 μL; 25 mM), dNTPs (0.4 μL; 10 mM), AmpliTaq Gold (0.1 μL), and RNase-free water (13.9 μL), all from Thermo. Conditions were: 95°C for 10 minutes, followed by 40 cycles of 95°C for 10 seconds and 60°C for 60 seconds. *Nlrp3* (5′–3′) (TCACAACTCGCCCAAGGAGGAA; AAGAGACCACGGCAGAAGCTA G), *Asc* (CTGCTCAGAG TACAGCCAGAAC; CTGTCCTTCAGTCAGCACACTG), *Casp1* (GGCACATTTCCAGGACTGACT G; GCAAGACGTGTACGAGTGGTTG), and *Gusb* (GGAGGTACTTCAGCTCTGTGAC; TGCCGAA GTGACTCGTTGCCAA) primers (1 μm; 1 μM) were mixed with SsoAdvanced Universal SYBR Green Supermix (5μl; Bio-Rad), cDNA (0.5 μL) and RNase-free water (2.5 μL). Conditions were 95°C for 30 seconds, followed by 40 cycles of 95°C for 5 seconds, 64°C for 15 seconds, and 72°C for 10 seconds, ending with a melt curve. The 2^–ΔΔCt^ method was used to analyze data generated by a CFX Connect Real-Time PCR Detection System (Bio-Rad). IL-1α/β, IL-6 and TNF-α in conditioned media and serum was measured by Cytometric Bead Array flex sets (BD), as per instructions, and quantified by flow cytometry (Accuri C6).

### Histology, morphometry, and immunofluorescence.

Aortic roots were fixed in 10% formalin (4°C; 16 hours), before processing, paraffin embedding, 5 μm serial sectioning and H&E staining. Plaque area was identified by H&E and quantified at every 100 μm from the start of the valve leaflets. Plaque indices included the largest single plaque, the section with highest plaque burden (peak plaque), and total plaque quantity across all 6 sections (i.e., AUC). For ASC specks, BMDMs on coverslips were washed (PBS), formalin fixed (2%; 15 minutes; RT), washed, permeabilized with NP-40 (0.5%; 2 minutes; RT), blocked with goat serum/BSA (5%/1% in PBS; 1 hour; RT), and incubated (16 hours; 4°C) with anti-Asc (MP5-20F3; 1:500; CST). After washing (Tween 0.05% in PBS) cells were incubated (1 hour; RT) with goat anti-rabbit AF488 (A-11008; 1:500), washed, and mounted (Prolong Gold; both Molecular Probes). Imaging was performed on a BX51 (Olympus) using Image-Pro Insight 9.1 software (Media Cybernetics).

### Mendelian randomization.

Genetic instruments for cytokines were based on known biological understanding of the function of the gene region in which they are located. Instruments were constructed using previously published GWAS, and comprised cis-acting variants (*P*_SNP-cytokine_ < 1 × 10^–4^, in or ± 500 kb from the gene encoding the relevant cytokine) in weak linkage disequilibrium (LD) (*r*^2^ < 0.10) (Supplemental Table 4) (60–63). Genetic instruments for CVDs were accessed from the MRCIEU OpenGWAS and comprised independent variants at genome-wide significance (*r*^2^ < 0.001, *P* < 5 × 10^–8^) (Supplemental Table 5). CHIP outcome summary statistics were from GWAS of UK BioBank individuals downloaded locally from the NHGRI-EBI GWAS Catalog (7, 64). Methodology for CHIP calling and GWAS are detailed in the respective publications (7). MR analyses were performed in R (version 4.3.1) using the Mendelian randomization package (version 0.10.0). Primary analyses of genetically predicted CVD used the inverse-variance weighted method, with MR-Egger, weighted median, simple mode, and weighted mode used in supplemental analyses. For cytokines instrumented by a single variant, the Wald ratio was used to compute effect estimates. For cytokines instrumented by 2 or more variants, MR estimates were computed using inverse-variance weighted fixed-effects (≤3 variants) or random-effects (>3 variants) models, accounting for weak LD between variants with reference to the UK BioBank European ancestry. CIs for estimates indicate power of the analysis by providing the range of plausible values MR estimates could take. Wide CIs signify insufficient power to rule out the possibility of a moderate causal effect.

### Statistics.

Data are presented as mean ± SEM, unless otherwise stated. All statistical analyses were carried out using Prism 10 (GraphPad). All assays that produced continuous data were performed in duplicate, with the exception of flow cytometry and animal experiments. *n* = an individual experimental replicate performed with a different Mɸ differentiation or an individual mouse — never a technical replicate. Before statistical testing for significance, data were analyzed for normality with a Shapiro-Wilks test, with normal distribution analyzed by parametric. Analysis of continuous data between 2 groups used paired or unpaired *t* test (2-tailed), with 3 or more groups using ANOVA (1-way), with Dunnett’s or Tukey’s post hoc tests. Serial trends of clonal growth or plaque was compared between groups using generalized linear mixed models. Relative transcript presented is from log transformed ΔΔCT values relative to *Gusb*, but statistics were performed on the ΔCT values.

### Data availability.

In addition to the Supporting Data Values file, data and supporting information are available from the corresponding author upon reasonable request. All details of genetic instruments and variants are in Supplemental Tables 1–5. All materials are from Sigma-Aldrich unless stated otherwise.

### Study approval.

Animal protocols were performed under UK Home Office licensing with local AWERB approval (Cambridge). MR analyses used anonymized human data previously deposited or via the UKBB.

## Author contributions

PRC, LK, ACR, and CZ designed and performed experiments and analyzed data. NF processed histological specimens. AZ and ZW analyzed data. PL, SB, and GSV provided helpful discussion. MCHC conceived the project, obtained funding, designed and performed experiments, analyzed data, and wrote the manuscript.

## Funding support

- British Heart Foundation Grants FS/13/3/30038, FS/18/19/33371, RG/16/8/32388, FS/20/19/34976, and SP/F/22/150038 to MC
- BHF Cambridge Centre for Research Excellence RE/18/1/34212 and RE/24/130011
- Cambridge NIHR Biomedical Research Centre

## Supplementary Material

Supplemental data

Supporting data values

## Version 1 12/23/2025

In-Press Preview

## Version 2 02/09/2026

Electronic publication

## References

[1] Jaiswal S (2014). Age-related clonal hematopoiesis associated with adverse outcomes. N Engl J Med.

[2] Genovese G (2014). Clonal hematopoiesis and blood-cancer risk inferred from blood DNA sequence. N Engl J Med.

[3] Xie M (2014). Age-related mutations associated with clonal hematopoietic expansion and malignancies. Nat Med.

[4] Vlasschaert C (2023). A practical approach to curate clonal hematopoiesis of indeterminate potential in human genetic data sets. Blood.

[5] Jaiswal S (2017). Clonal hematopoiesis and risk of atherosclerotic cardiovascular disease. N Engl J Med.

[6] Bick AG (2020). Genetic interleukin 6 signaling deficiency attenuates cardiovascular risk in clonal hematopoiesis. Circulation.

[7] Kessler MD (2022). Common and rare variant associations with clonal hematopoiesis phenotypes. Nature.

[8] Vlasschaert C (2023). Interleukin-6 receptor polymorphism attenuates clonal hematopoiesis-mediated coronary artery disease risk among 451 180 individuals in the UK Biobank. Circulation.

[9] Laclaustra M (2016). Femoral and carotid subclinical atherosclerosis association with risk factors and coronary calcium: the AWHS study. J Am Coll Cardiol.

[10] Khot UN (2003). Prevalence of conventional risk factors in patients with coronary heart disease. JAMA.

[11] Libby P (2013). Immune effector mechanisms implicated in atherosclerosis: from mice to humans. Immunity.

[12] Diez-Diez M (2024). Unidirectional association of clonal hematopoiesis with atherosclerosis development. Nat Med.

[13] Zhao K (2024). Somatic and germline variants and coronary heart disease in a Chinese population. JAMA Cardiol.

[14] Kar SP (2022). Genome-wide analyses of 200,453 individuals yield new insights into the causes and consequences of clonal hematopoiesis. Nat Genet.

[15] Zekavat SM (2023). *TP53-*mediated clonal hematopoiesis confers increased risk for incident atherosclerotic disease. Nat Cardiovasc Res.

[16] Fuster JJ (2017). Clonal hematopoiesis associated with TET2 deficiency accelerates atherosclerosis development in mice. Science.

[17] Zuriaga MA (2024). Colchicine prevents accelerated atherosclerosis in TET2-mutant clonal hematopoiesis. Eur Heart J.

[18] Liu W (2023). Blockade of IL-6 signaling alleviates atherosclerosis in *Tet2*-deficient clonal hematopoiesis. Nat Cardiovasc Res.

[19] Rauch PJ (2023). Loss-of-function mutations in Dnmt3a and Tet2 lead to accelerated atherosclerosis and concordant macrophage phenotypes. Nat Cardiovasc Res.

[20] Wang W (2018). Macrophage inflammation, erythrophagocytosis, and accelerated atherosclerosis in Jak2 ^V617F^ Mice. Circ Res.

[21] Fidler TP (2021). The AIM2 inflammasome exacerbates atherosclerosis in clonal hematopoiesis. Nature.

[22] Svensson EC (2022). TET2-driven clonal hematopoiesis and response to canakinumab: an exploratory analysis of the CANTOS randomized clinical trial. JAMA Cardiol.

[23] Abram CL (2014). Comparative analysis of the efficiency and specificity of myeloid-Cre deleting strains using ROSA-EYFP reporter mice. J Immunol Methods.

[24] Kaasinen E (2019). Impact of constitutional TET2 haploinsufficiency on molecular and clinical phenotype in humans. Nat Commun.

[25] Buscarlet M (2017). *DNMT3A* and *TET2* dominate clonal hematopoiesis and demonstrate benign phenotypes and different genetic predispositions. Blood.

[26] Arends CM (2018). Hematopoietic lineage distribution and evolutionary dynamics of clonal hematopoiesis. Leukemia.

[27] Cook EK (2019). Comorbid and inflammatory characteristics of genetic subtypes of clonal hematopoiesis. Blood Adv.

[28] Dawoud AAZ (2020). Clonal myelopoiesis in the UK Biobank cohort: ASXL1 mutations are strongly associated with smoking. Leukemia.

[29] Zhang Q (2015). Tet2 is required to resolve inflammation by recruiting Hdac2 to specifically repress IL-6. Nature.

[30] Li R (2020). *JAK2^V617F^* Mutation promoted IL-6 production and glycolysis via mediating PKM1 stabilization in macrophages. Front Immunol.

[31] Dotan I (2022). Macrophage Jak2 deficiency accelerates atherosclerosis through defects in cholesterol efflux. Commun Biol.

[32] Heyde A (2021). Increased stem cell proliferation in atherosclerosis accelerates clonal hematopoiesis. Cell.

[33] Uddin MM (2024). Long-term longitudinal analysis of 4,187 participants reveals insights into determinants of clonal hematopoiesis. Nat Commun.

[34] Boettcher S, Manz MG (2017). Regulation of inflammation- and infection-driven hematopoiesis. Trends Immunol.

[35] Weisser M (2016). Hyperinflammation in patients with chronic granulomatous disease leads to impairment of hematopoietic stem cell functions. J Allergy Clin Immunol.

[36] Pietras EM (2016). Chronic interleukin-1 exposure drives hematopoietic stem cells towards precocious myeloid differentiation at the expense of self-renewal. Nat Cell Biol.

[37] Al Ustwani O (2013). Myelodysplastic syndromes and autoimmune diseases--case series and review of literature. Leuk Res.

[38] Kristinsson SY (2011). Chronic immune stimulation might act as a trigger for the development of acute myeloid leukemia or myelodysplastic syndromes. J Clin Oncol.

[39] Reynaud D (2011). IL-6 controls leukemic multipotent progenitor cell fate and contributes to chronic myelogenous leukemia development. Cancer Cell.

[40] Ganan-Gomez I (2015). Deregulation of innate immune and inflammatory signaling in myelodysplastic syndromes. Leukemia.

[41] Welner RS (2015). Treatment of chronic myelogenous leukemia by blocking cytokine alterations found in normal stem and progenitor cells. Cancer Cell.

[42] Rambaldi A (1993). Modulation of cell proliferation and cytokine production in AML by recombinant interleukin-1 receptor antagonist. Leukemia.

[43] Stifter G (2005). Over-expression of tumor necrosis factor-alpha in bone marrow biopsies from patients with myelodysplastic syndromes: relationship to anemia and prognosis. Eur J Hematol.

[44] Erren M (1999). Systemic inflammatory parameters in patients with atherosclerosis of the coronary and peripheral arteries. Arterioscler Thromb Vasc Biol.

[45] Clarke MC (2010). Vascular smooth muscle cell apoptosis induces interleukin-1-directed inflammation: effects of hyperlipidemia-mediated inhibition of phagocytosis. Circ Res.

[46] Gardner SE (2015). Senescent vascular smooth muscle cells drive inflammation through an interleukin-1α-dependent senescence-associated secretory phenotype. Arterioscler Thromb Vasc Biol.

[47] Duewell P (2010). NLRP3 inflammasomes are required for atherogenesis and activated by cholesterol crystals. Nature.

[48] Ko M (2011). Ten-Eleven-Translocation 2 (TET2) negatively regulates homeostasis and differentiation of hematopoietic stem cells in mice. Proc Natl Acad Sci U S A.

[49] Li J (2014). JAK2V617F homozygosity drives a phenotypic switch in myeloproliferative neoplasms, but is insufficient to sustain disease. Blood.

[50] Gozdecka M (2025). Mitochondrial metabolism sustains DNMT3A-R882-mutant clonal hematopoiesis. Nature.

[51] Jafri S (2017). A sex-specific reconstitution bias in the competitive CD45.1/CD45.2 congenic bone marrow transplant model. Sci Rep.

[52] Bosco N (2010). Auto-reconstitution of the T-cell compartment by radioresistant hematopoietic cells following lethal irradiation and bone marrow transplantation. Exp Hematol.

[53] Ridker PM (2017). Antiinflammatory therapy with canakinumab for atherosclerotic disease. N Engl J Med.

[54] Hormaechea-Agulla D (2021). Chronic infection drives Dnmt3a-loss-of-function clonal hematopoiesis via IFNγ signaling. Cell Stem Cell.

[55] Liao M (2022). Aging-elevated inflammation promotes DNMT3A R878H-driven clonal hematopoiesis. Acta Pharm Sin B.

[56] Caiado F (2023). Aging drives Tet2^+/–^ clonal hematopoiesis via IL-1 signaling. Blood.

[57] Stacey SN (2023). Genetics and epidemiology of mutational barcode-defined clonal hematopoiesis. Nat Genet.

[58] Gozdecka M (2019). Genetic vulnerabilities of DNMT3AR882H in myeloid malignancies. Blood.

[59] Horai R (1998). Production of mice deficient in genes for interleukin (IL)-1alpha, IL-1beta, IL-1alpha/beta, and IL-1 receptor antagonist shows that IL-1beta is crucial in turpentine-induced fever development and glucocorticoid secretion. J Exp Med.

[60] Karhunen V (2023). The interplay between inflammatory cytokines and cardiometabolic disease: bi-directional mendelian randomisation study. BMJ Med.

[61] Yang Z (2021). Mendelian randomization study of interleukin (IL)-1 family and lung cancer. Sci Rep.

[62] Yarmolinsky J (2024). Association between circulating inflammatory markers and adult cancer risk: a Mendelian randomization analysis. EBioMedicine.

[63] Collaboration IRGCERF (2012). Interleukin-6 receptor pathways in coronary heart disease: a collaborative meta-analysis of 82 studies. Lancet.

[64] Sollis E (2023). The NHGRI-EBI GWAS Catalog: knowledgebase and deposition resource. Nucleic Acids Res.

