# Supplementary material for: Common clonal hematopoiesis driver mutations have disparate effects on macrophage cytokines, clonal expansion, and atherogenesis
Source: JCI Insight. 2025 Dec 23;11(3):e200334. doi: 10.1172/jci.insight.200334 (PMC12892922; doi:10.1172/jci.insight.200334)
Supplement: Supplemental data [file jciinsight-11-200334-s082.pdf]

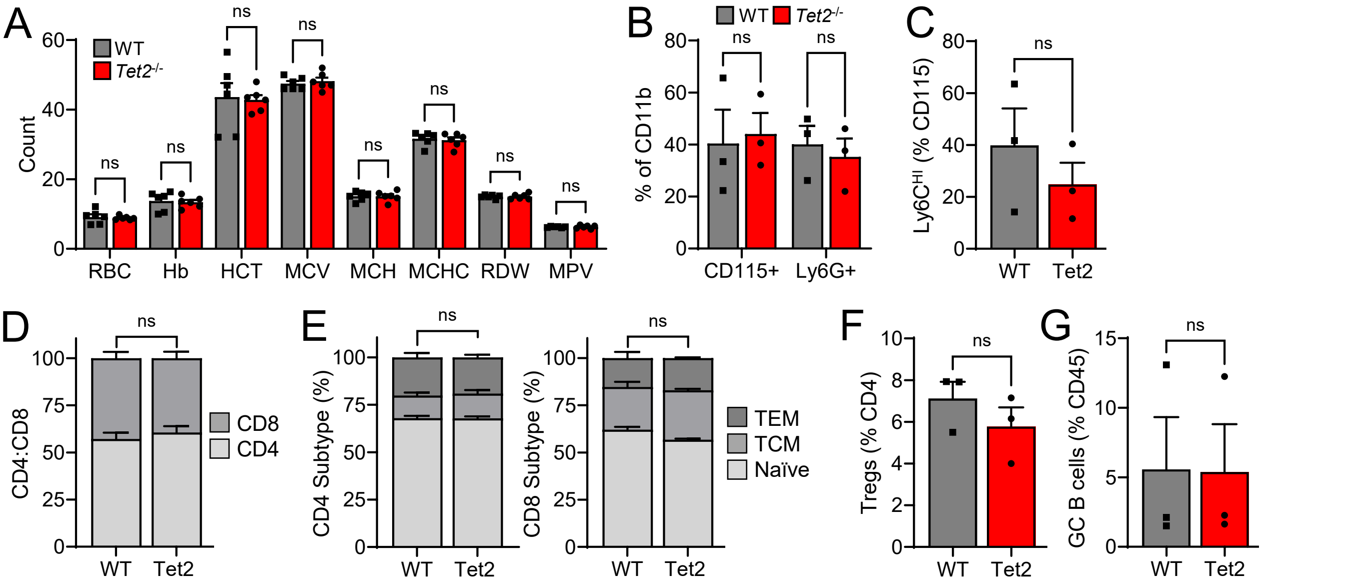

Figure S1: *Tet2*$^{-/-}$ mutant mice show normal blood counts and immune parameters under the steady state. (A-C) Red blood cell indices by automated counting **(A)**, and CD115^{+}, Ly6G^{+} **(B)** or Ly6C^{HI} cells in the circulation by flow cytometry **(C)** in WT (grey) and *Tet2*$^{-/-}$ (red) mice. **(D-G)** Flow cytometry of spleens for CD4/8 T cell ratio **(D)**, CD4/8 T cell subtype **(E)**, Tregs **(F)** and germinal centre (GC) B cells **(G)** in WT and *Tet2*$^{-/-}$ mice under the steady state. RBC = red blood cell; Hb = haemoglobin; HCT = haematocrit; MCV = mean corpuscular volume; MCH = mean cell haemoglobin; MCHC = MCH concentration; RDW = red cell distribution width; MPV = mean platelet volume; TCM = central memory; TEM = effector memory. Data represent mean ±SEM of n = 6/6 **(A)**, 3/3 **(B-G)**; ns = not significant; by unpaired t-test **(A-D,F,G)** or ANOVA **(E)**.

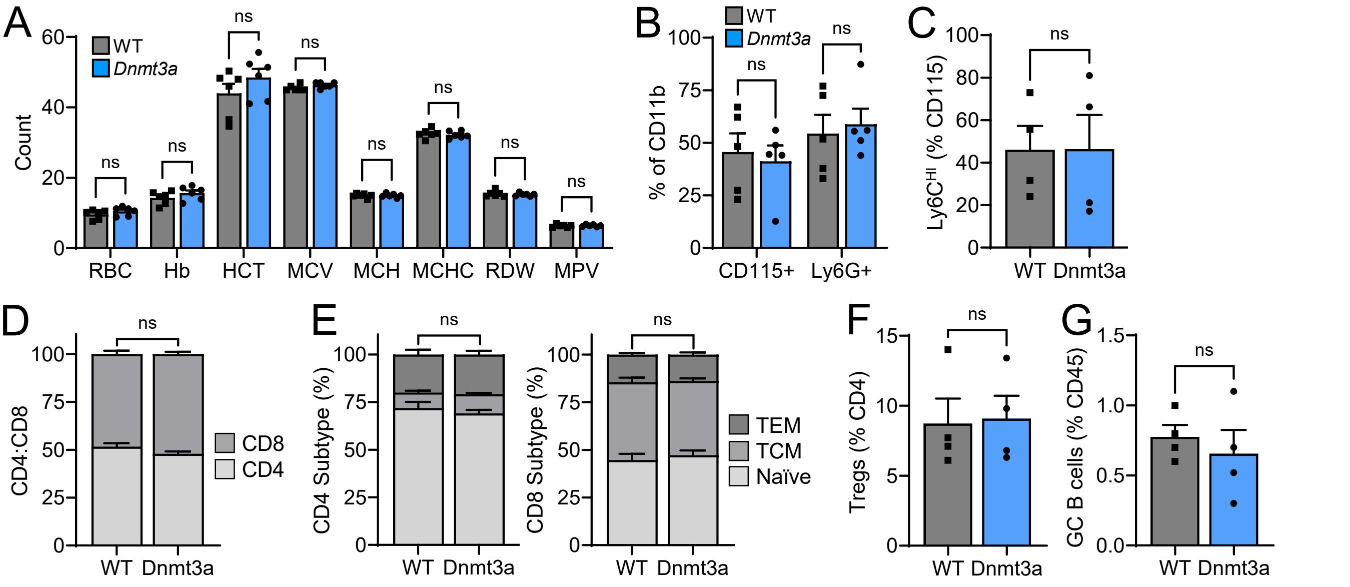

Figure S2: *Dnmt3a*$^{RH/RH}$ mutant mice show normal blood counts and immune parameters under the steady state. (A-C) Red blood cell indices by automated counting **(A)**, and CD115^{+}, Ly6G^{+} **(B)** or Ly6C^{HI} cells in the circulation by flow cytometry **(C)** in wild-type (WT; grey) and *Dnmt3a*$^{RH/RH}$ (blue) mice. **(D-G)** Flow cytometry of spleens for CD4/8 T cell ratio **(D)**, CD4/8 T cell subtype **(E)**, Tregs **(F)** and germinal centre (GC) B cells **(G)** in WT and *Dnmt3a*$^{RH/RH}$ mice under the steady state. RBC = red blood cell; Hb = haemoglobin; HCT = haematocrit; MCV = mean corpuscular volume; MCH = mean cell haemoglobin; MCHC = MCH concentration; RDW = red cell distribution width; MPV = mean platelet volume; TCM = central memory; TEM = effector memory. Data represent mean ±SEM of n = 6/6 **(A)**, 5/5 **(B),** 4/4 **(C-G)**; ns = not significant; by unpaired t-test **(A-D,F,G)** or ANOVA **(E)**..

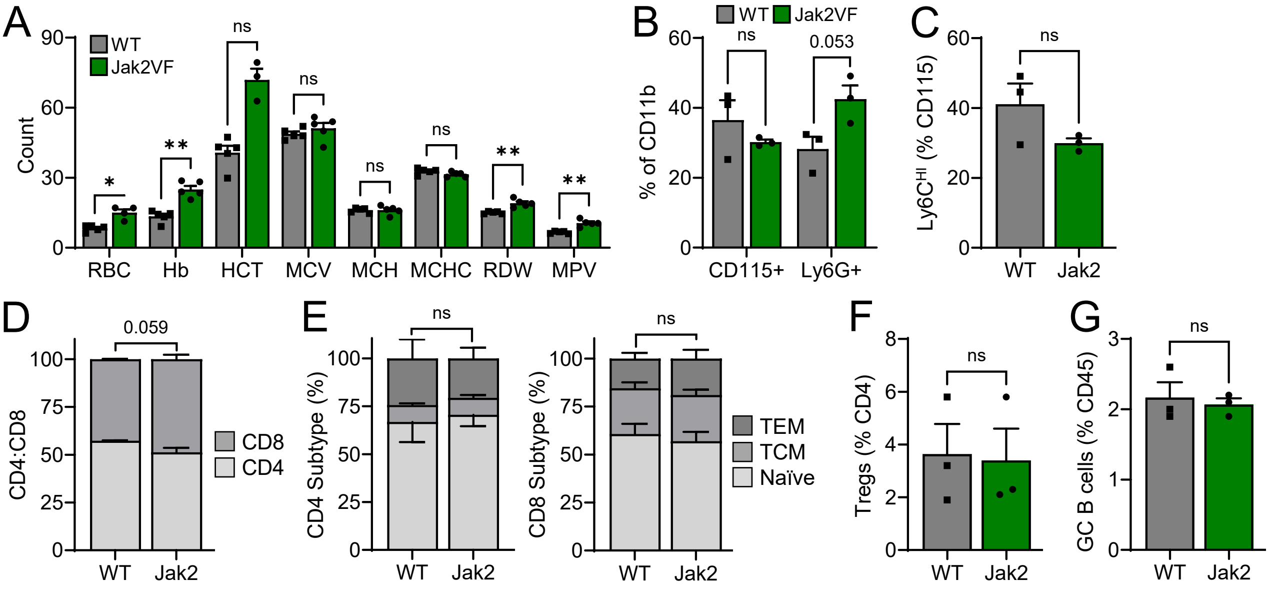

Figure S3: *Jak2*$^{VF/VF}$ mutant mice show altered blood counts and adaptive immune parameters under the steady state. (A-C) Red blood cell indices by automated counting **(A)**, and CD115^{+}, Ly6G^{+} **(B)** or Ly6C^{HI} cells in the circulation by flow cytometry **(C)** in WT (grey) and *Jak2*$^{VF/VF}$ (green) mice. **(D-G)** Flow cytometry of spleens for CD4/8 T cell ratio **(D)**, CD4/8 T cell subtype **(E)**, Tregs **(F)** and germinal centre (GC) B cells **(G)** in WT and *Jak2*$^{VF/VF}$ mice under the steady state. RBC = red blood cell; Hb = haemoglobin; HCT = haematocrit; MCV = mean corpuscular volume; MCH = mean cell haemoglobin; MCHC = MCH concentration; RDW = red cell distribution width; MPV = mean platelet volume; TCM = central memory; TEM = effector memory. Data represent mean ±SEM of n = 5/3-5 **(A)**, 3/3 **(B-G)**; p = *≤0.05, **≤0.01, or as stated; ns = not significant; by unpaired t-test **(A-D,F,G)** or ANOVA **(E)**.

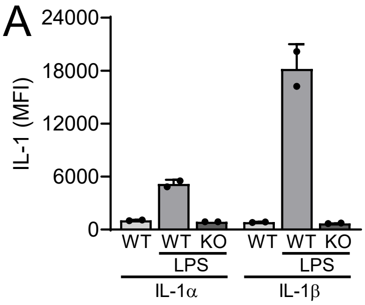

Figure S4: Specificity of intracellular cytokine staining and example images for 'ASC specks'. (A) Flow cytometry for intracellular IL-1α/β in ±LPS treated Mϕ from WT, or *Il1a* and *Il1b* KO mice. **(B)** Representative images of bone marrow-derived macrophages from WT or *Tet2*$^{-/-}$ and *Dnmt3a*$^{RH/RH}$ mutant mice treated with LPS or LPS/nigericin, followed by immunofluorescence staining for ASC specks (arrows). Data represent mean ±SD of n = 2 **(A)**.

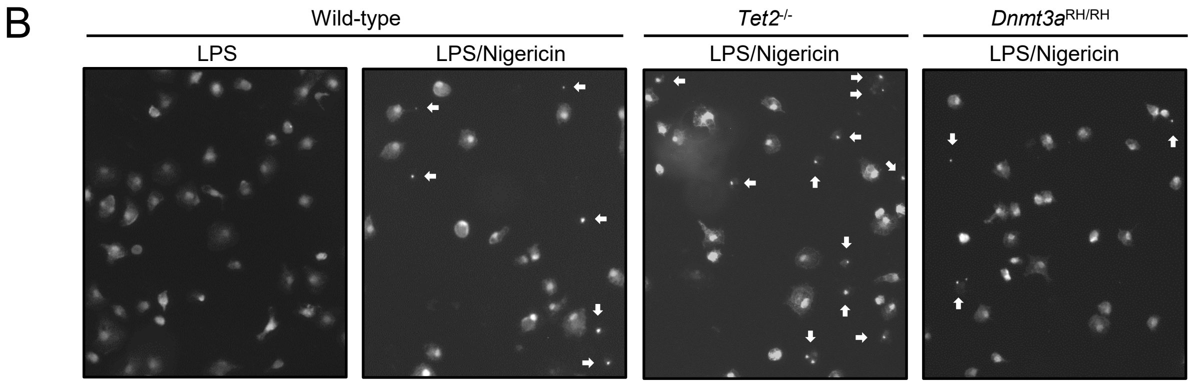

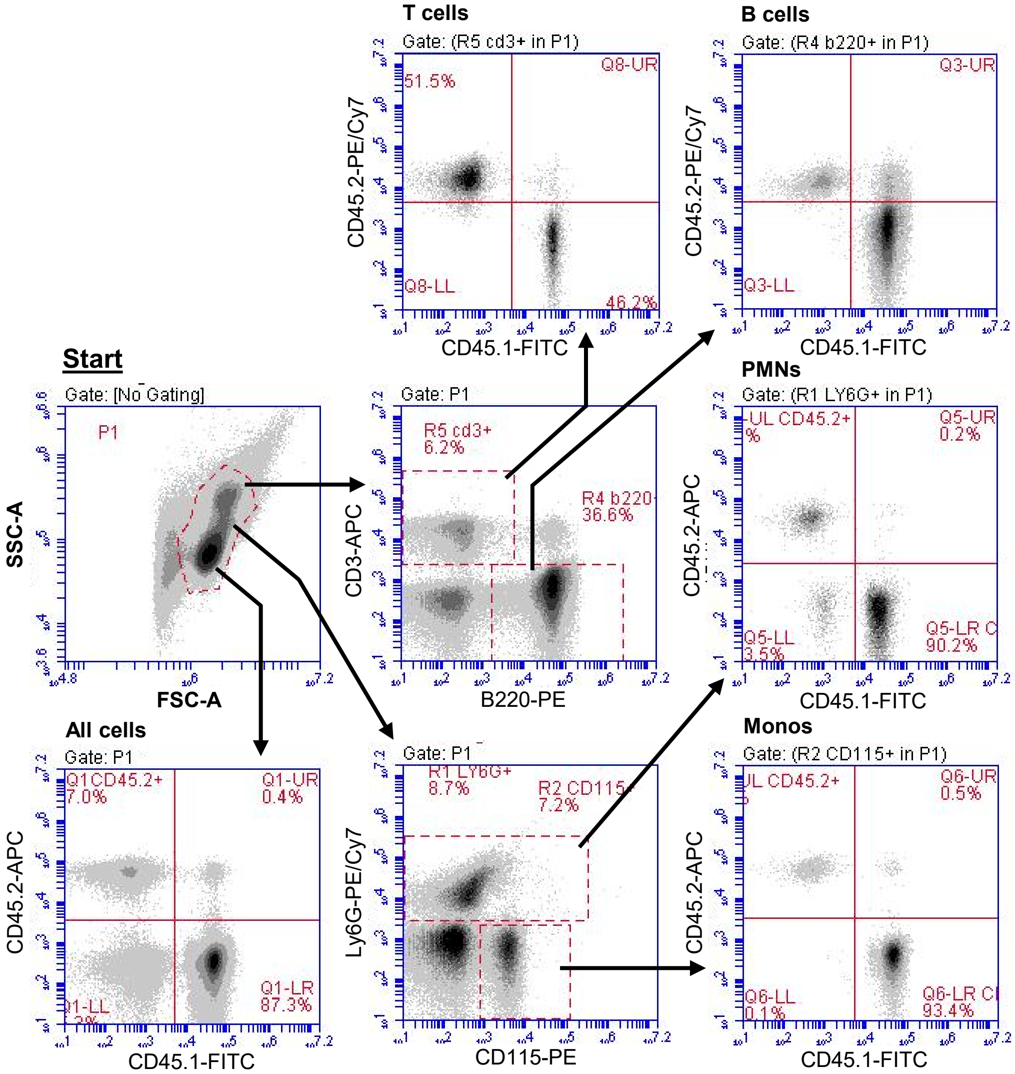

Figure S5: Example flow plots showing gating for CD45.1+ and CD45.2+ cells in various lineages. Whole blood from CD45.1 mice transplanted with CD45.2 mutant bone marrow was stained for the lineage markers CD115 (monocytes (monos)), Ly6G (neutrophils(PMNs)), B220 (B cells) and CD3 (T cells), and the level of CD45.1 and CD45.2 enumerated in the gated populations.

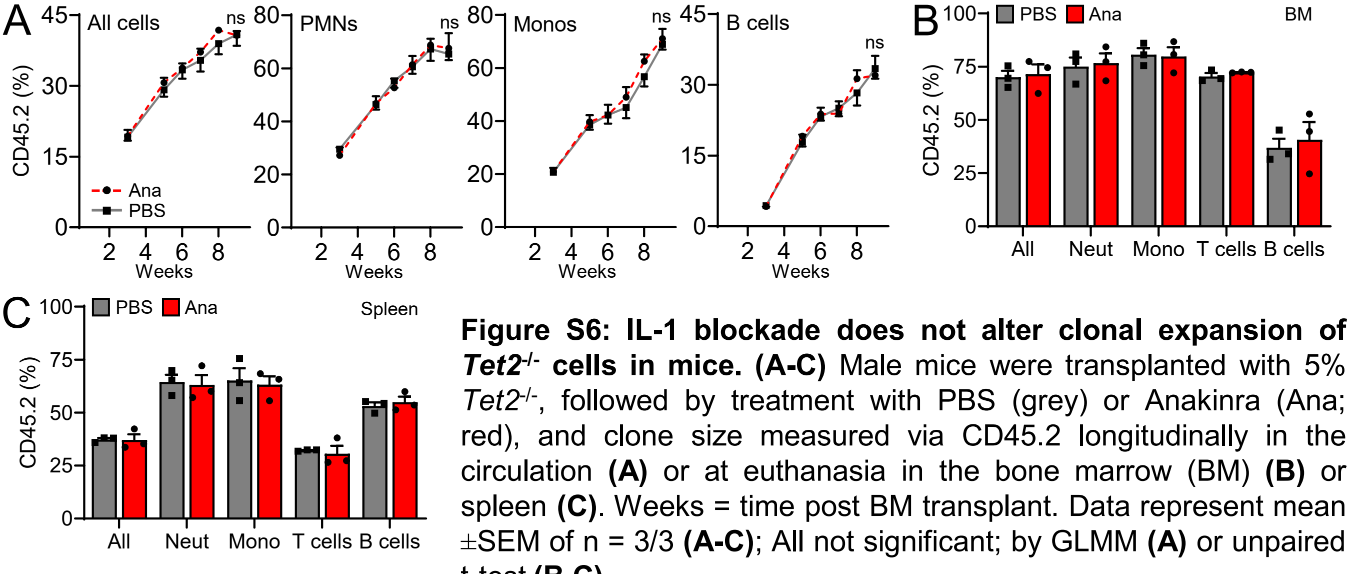

Figure S6: IL-1 blockade does not alter clonal expansion of *Tet2*$^{-/-}$ cells in mice. (A-C) Male mice were transplanted with 5% *Tet2*$^{-/-}$, followed by treatment with PBS (grey) or Anakinra (Ana; red), and clone size measured via CD45.2 longitudinally in the circulation **(A)** or at euthanasia in the bone marrow (BM) **(B)** or spleen **(C)**. Weeks = time post BM transplant. Data represent mean ±SEM of n = 3/3 **(A-C)**; All not significant; by GLMM **(A)** or unpaired t-test **(B,C)**.

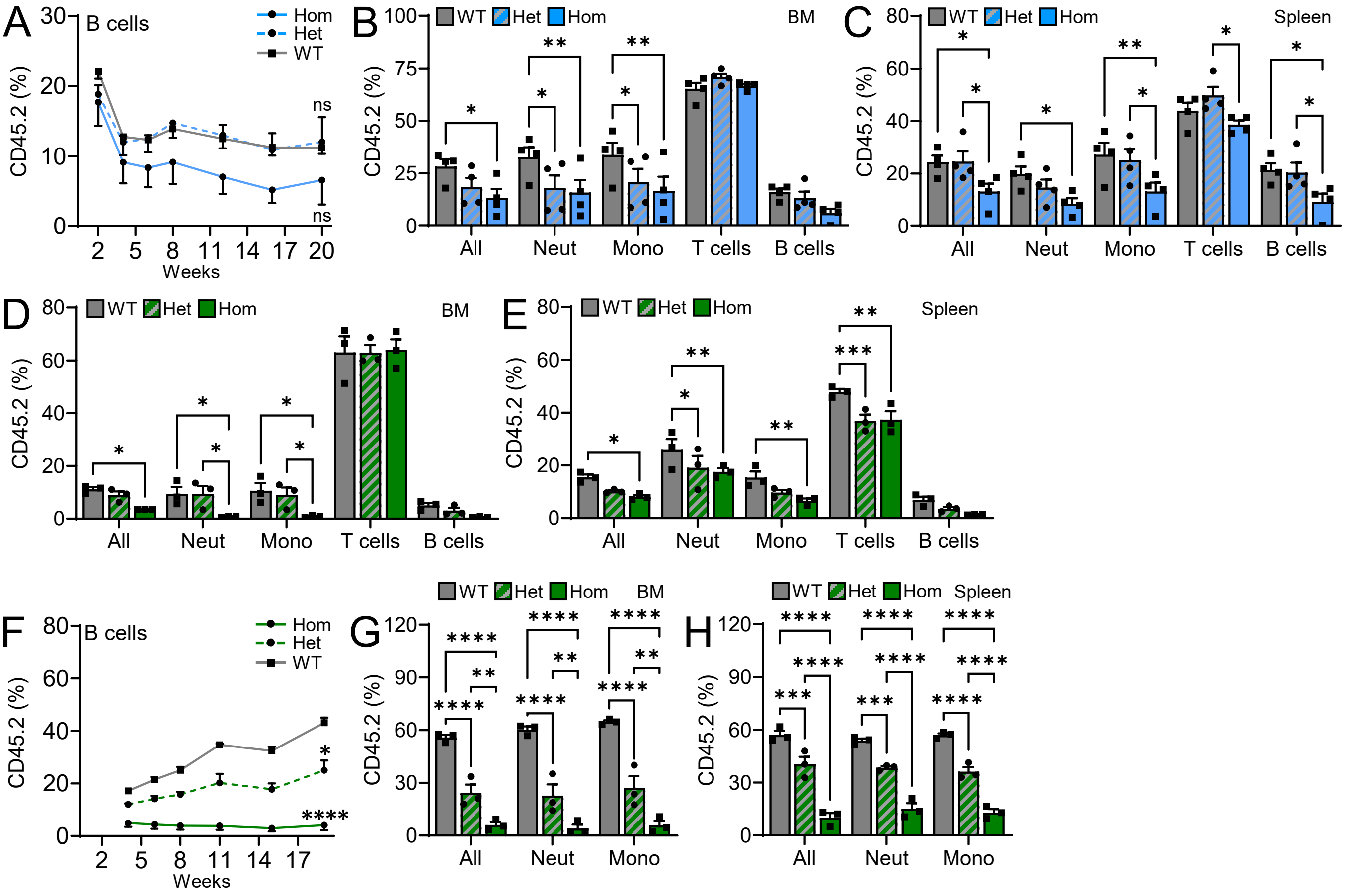

Figure S7: *Dnmt3a*^{RH} or *Jak2*^{VF} mutations do not cause clonal expansion of B cells or cells in other tissues. (A-C) Longitudinal measurement of clone size via CD45.2 in circulation **(A)** or at euthanasia in the bone marrow (BM) **(B)** or spleen **(C)** in male mice transplanted with WT (grey) 10% *Dnmt3a*^{RH} heterozygous (Het) or homozygous (Hom) mutant cells (blue). **(D,E)** Measurement of clone size via CD45.2 at euthanasia in the BM **(D)** or spleen **(E)** in male mice transplanted with 5% *Jak2*^{VF} Het or Hom mutant cells (green). **(F-H)** Longitudinal measurement of clone size via CD45.2 in the circulation **(F)** or at euthanasia in the BM **(G)** or spleen **(H)** in male mice transplanted with 30% *Jak2*^{VF} Het or Hom mutant cells. Weeks = time post BM transplant. Data represent mean ±SEM of n = 4/4 **(A-C)**, 3/3 **(D-H)**; p = *≤0.05, **≤0.01, ***≤0.001, ****≤0.0001; ns = not significant; by GLMM **(A,F)** or ANOVA **(B-E,G,H)**.

CHIP mutant	% Transplanted	Mean	SD	n=	Powered effect size	Observed Δ Het	Observed Δ Hom
Tet2 $^{-/-}$	10	17.12	3.58	3	±45%	+100%	+272%
Dntm3a ^{RH}	10	21.41	3.36	4	±30%	+0.5%	-42%
Jak2 ^{VF}	5	13.69	1.38	4	±20%	-37%	-61%
Jak2 ^{VF}	30	46.55	1.62	4	±7.5%	-35%	-87%

Figure S8: Clonal expansion experiments are powered to detect smaller effect sizes than the actual differences observed. Data as indicated for clone size (% of all cells in the circulation) in control transplanted groups, along with the effect size we are correctly powered (α=0.05; power=0.80) to detect and the observed difference (Δ) in heterozygous (Het) or homozygous (Hom) CHIP mutant clone size.

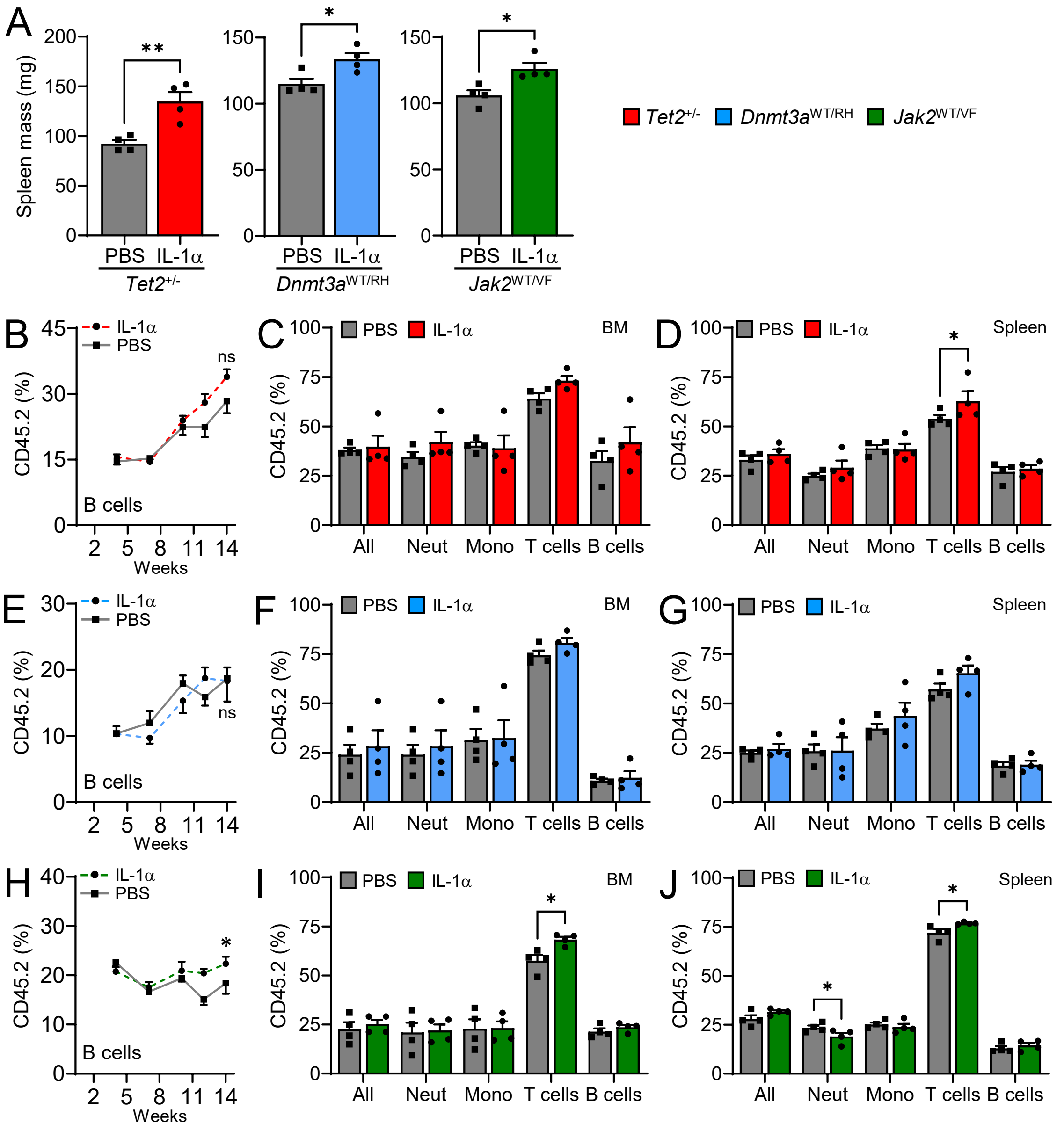

Figure S9: IL-1α does not increase clonal expansion of *Tet2*$^{+/-}$, *Dnmt3a*$^{WT/RH}$ or *Jak2*$^{WT/VF}$ cells. **(A)** Spleen weight in mice transplanted with 10% *Tet2*$^{+/-}$ (grey/red; male), 10% *Dnmt3a*$^{WT/RH}$ (grey/blue; female) or 30% *Jak2*$^{WT/VF}$ (grey/green; male) cells, then treated with PBS (grey) or IL-1α (colour). **(B-J)** Longitudinal measurement of clone size via CD45.2 in the circulation **(B,E,H)** or at euthanasia in the bone marrow (BM) **(C,F,I)** or spleen **(D,G,J)** in mice transplanted with 10% *Tet2*$^{+/-}$ (grey/red; male), 10% *Dnmt3a*$^{WT/RH}$ (grey/blue; female) or 30% *Jak2*$^{WT/VF}$ (grey/green; male) cells, and treated with PBS or IL-1α. Weeks = time post BM transplant. Data represent mean ±SEM of n = 4/4 **(A-J)**; p = *≤0.05, **≤0.01; ns = not significant; by GLMM **(B,E,H)** or unpaired t-test **(A,C,D,F,G,I,J)**.

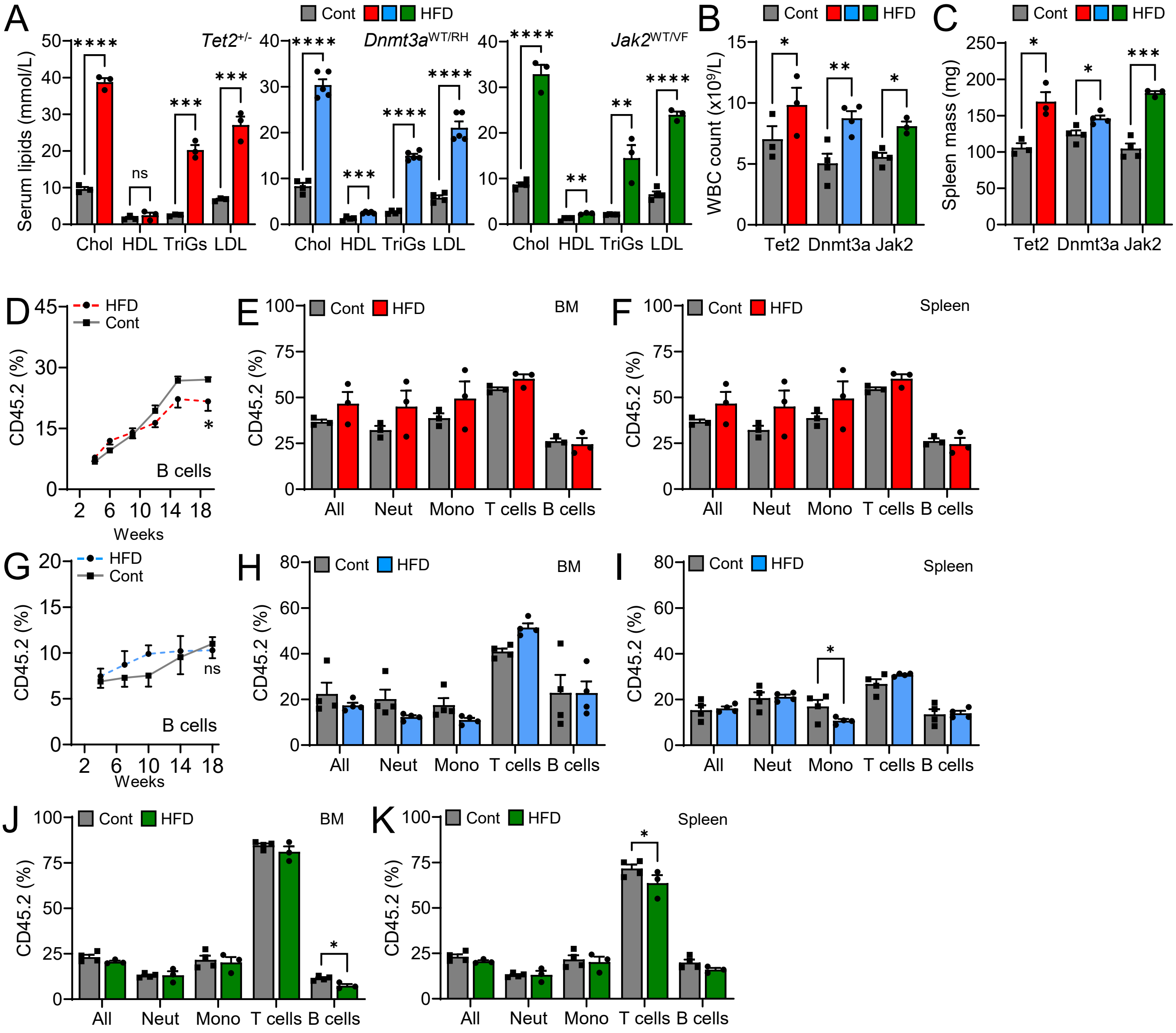

Figure S10: High fat diet does not increase clonal expansion of *Tet2*$^{+/-}$, *Dnmt3a*$^{WT/RH}$ or *Jak2*$^{WT/VF}$ B cells or cells in other tissues. (A-C) Serum lipids **(A)**, white blood cell (WBC)**(B)** count and spleen weight **(C)** in female mice transplanted with 10% *Tet2*$^{+/-}$ (grey/red), 10% *Dnmt3a*$^{WT/RH}$ (grey/blue) or 30% *Jak2*$^{WT/VF}$ (grey/green) cells, then fed a normal chow (Cont; grey) or high fat diet (HFD; colour). **(D-K)** Longitudinal measurement of clone size via CD45.2 in the circulation **(D,G)** or at euthanasia in the bone marrow (BM) **(E,H,J)** or spleen **(F,I,K)** in female mice transplanted with 10% *Tet2*$^{+/-}$ (grey/red), 10% *Dnmt3a*$^{WT/RH}$ (grey/blue) or 30% *Jak2*$^{WT/VF}$ (grey/green) cells, then fed a normal chow or HFD. Weeks = time post BM transplant. Data represent mean ±SEM of n = 3/3 (*Tet2*), 4/4 (*Dnmt3a*), 4/3 (*Jak2*); p = *≤0.05, **≤0.01, ***≤0.001, ****≤0.0001; ns = not significant; by t-test **(A-C,E,F,H-K)** or GLMM **(D,G)**.

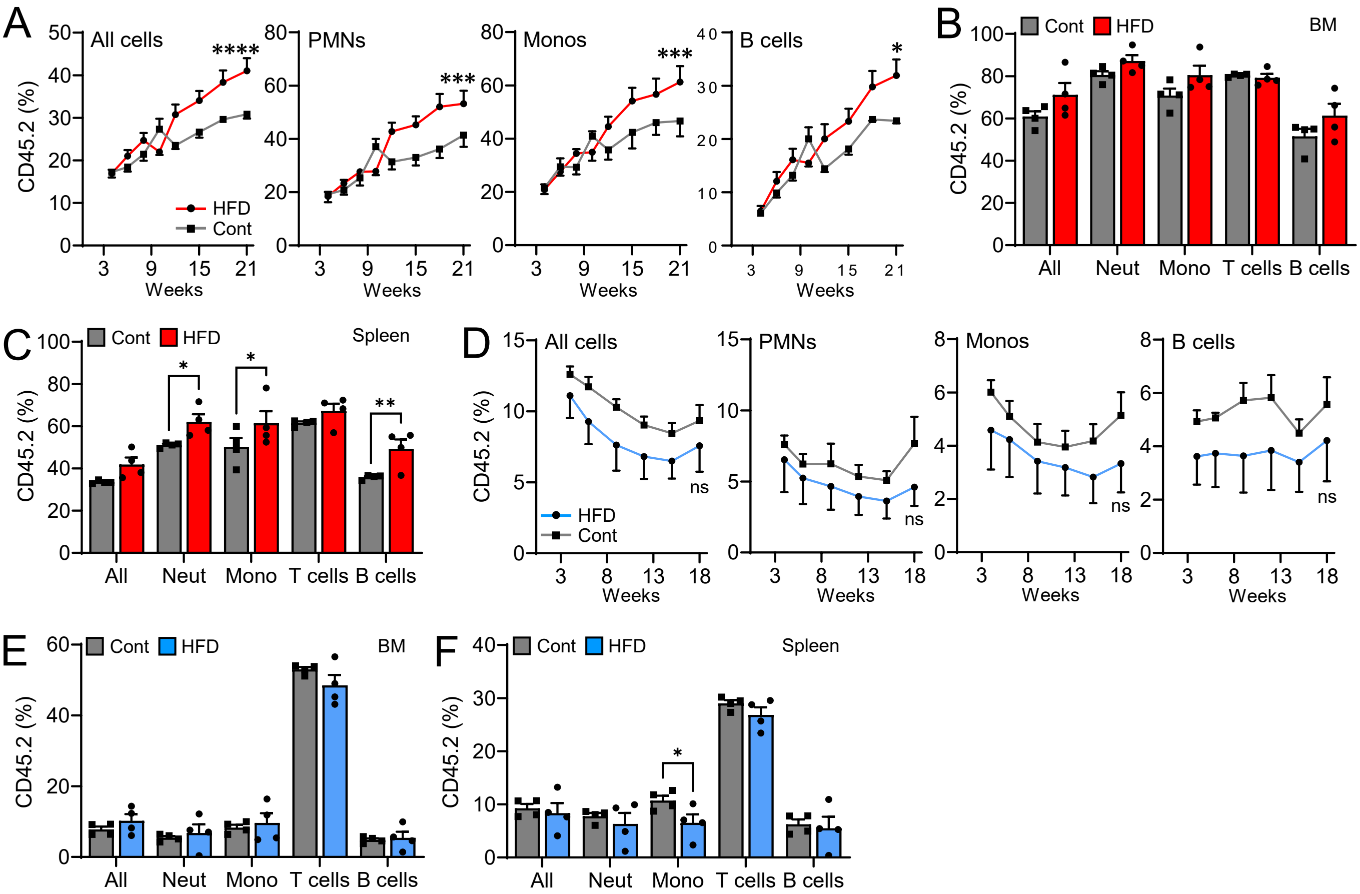

Figure S11: High fat diet increases clonal expansion of *Tet2*$^{-/-}$, but not *Dnmt3a*$^{RH/RH}$ mutant cells. (A,F) Longitudinal measurement of clone size via CD45.2 in the circulation **(A,D)** or at euthanasia in the bone marrow (BM) **(B,E)** or spleen **(C,F)** in male mice transplanted with 10% *Tet2*$^{+/-}$ (grey/red) or 10% *Dnmt3a*$^{WT/RH}$ (grey/blue) cells, then fed a normal chow (Cont; grey) or high fat diet (HFD; colour). Weeks = time post BM transplant. Data represent mean ±SEM of n = 4/4 **(A-F)**; p = *≤0.05, **≤0.01, ***≤0.001, ****≤0.0001; ns = not significant; by GLMM **(A,D)** or unpaired t-test **(B,C,E,F)**.

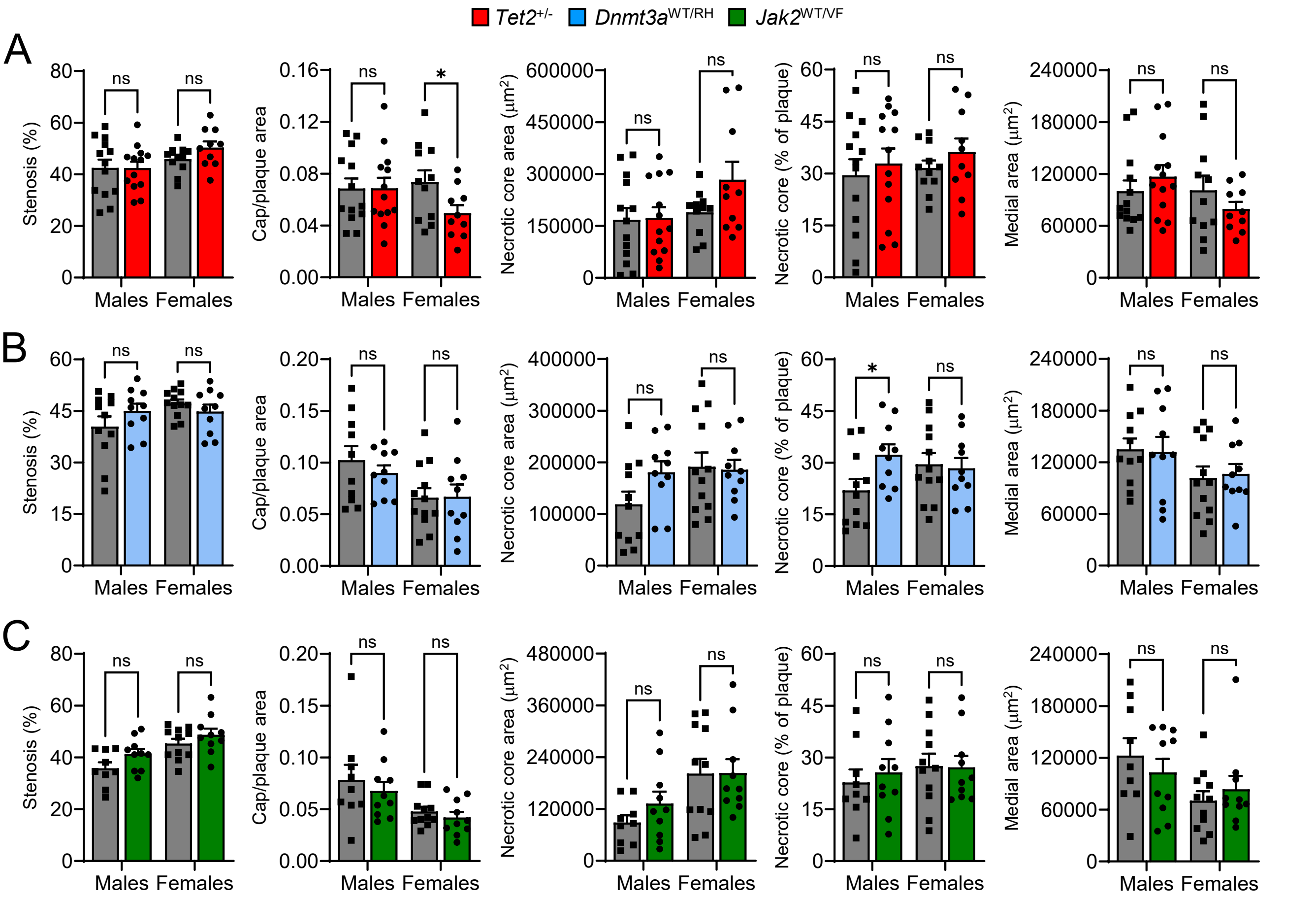

Figure S12: CHIP models have minimal effects on atherosclerotic plaque composition in mice. (A-C) Assessment of atherosclerotic plaque composition in male and female mice transplanted with 10% *Tet2*$^{+/-}$ (red) **(A)**, 10% *Dnmt3a*$^{WT/RH}$ (blue) **(B)**, or 30% *Jak2*$^{WT/VF}$ (green) **(C)** mutant cells, or equivalent WT cells (grey), followed by high fat feeding for 12 w. Mice were assessed for vessel stenosis, relative fibrous cap size, necrotic core area and percentage, and medial area. Data represent mean ±SEM of n = 13/13 (male) 11/10 (female)**(A)**, 10/10 (male) 12/10 (female)**(B)**, n = 9/10 (male) 11/10 (female)**(C)**; p = *≤0.05; ns = not significant; by unpaired t-test **(A-C)**.

Supplemental table 1: Mendelian randomisation estimates of genetically predicted serum cytokine levels and risk of CHIP in IVW analyses

Exposure							CHIP Overall								DNMT3A CHIP								TET2 CHIP							
Cytokine	GWAS Pubmed ID	IV Type	Cytokine Unit	R2 (%)	IVW model	nsnp	Beta	SE	p	OR	LCI	UCI	Coch Q	Coch Q_p	Beta	SE	p	OR	LCI	UCI	Coch Q	Coch Q_p	Beta	SE	p	OR	LCI	UCI	Coch Q	Coch Q_p
CRP	37205426	cis-pQTL	SD	2.2	Random	5	0.032	0.053	0.545	1.033	0.931	1.146	5.490	0.241	0.062	0.065	0.338	1.064	0.937	1.209	5.419	0.247	-0.089	0.111	0.425	0.915	0.736	1.138	3.643	0.457
IL-1RAP	29875488	cis-pQTL	SD of log	11.3	Random	6	0.030	0.026	0.255	1.030	0.979	1.085	8.921	0.112	0.058	0.033	0.080	1.060	0.993	1.132	9.613	0.087	0.000	0.048	0.992	1.000	0.909	1.099	0.261	0.998
IL-1RA	36936266	cis-pQTL	SD	5.5	Random	23	0.048	0.034	0.150	1.049	0.983	1.121	14.239	0.893	0.017	0.041	0.675	1.017	0.938	1.103	18.205	0.694	0.062	0.082	0.455	1.063	0.905	1.250	20.744	0.537
IL-1R1	37205426	cis-pQTL	SD	1.0	Random	5	0.010	0.061	0.871	1.010	0.896	1.138	2.124	0.713	0.035	0.081	0.660	1.036	0.885	1.213	4.640	0.326	-0.007	0.149	0.960	0.993	0.741	1.330	0.591	0.964
IL-18	36936266	cis-pQTL	SD	4.5	Random	7	-0.062	0.036	0.089	0.940	0.876	1.009	4.749	0.576	-0.104	0.051	0.040	0.901	0.816	0.995	7.775	0.255	-0.130	0.089	0.144	0.878	0.737	1.046	1.869	0.931
IL-6	22421339	cis-pQTL	% increase	1.0	Fixed	1	-0.076	0.065	0.240	0.926	0.816	1.052	NA	NA	-0.042	0.080	0.600	0.959	0.820	1.122	NA	NA	-0.161	0.159	0.312	0.851	0.623	1.163	NA	NA
MCSF	36936266	cis-pQTL	SD	3.1	Random	12	-0.025	0.048	0.607	0.975	0.887	1.072	12.801	0.307	-0.031	0.058	0.597	0.970	0.865	1.087	12.207	0.348	-0.042	0.110	0.703	0.959	0.773	1.190	7.825	0.729
TNFa	36936266	cis-pQTL	SD	0.5	Fixed	2	-0.164	0.099	0.100	0.849	0.699	1.032	0.159	0.690	-0.184	0.122	0.132	0.832	0.655	1.057	0.006	0.938	-0.194	0.244	0.425	0.823	0.510	1.328	0.017	0.895

Abbreviations: nsnp, number of Single-nucleotide polymorphism; GWAS, Genome-wide association study; IV, Instrumental variant; IVW, Inverse-variance weighted; SD, Standard deviation; SE, standard error; p, p-value; OR, Odds ratio; LCI, lower 95% confidence interval of odds ratio; UCI, upper 95% confidence interval of odds ratio; CHIP, clonal hematopoiesis of indeterminate potential; Coch Q, Cochran's Q value.

Supplemental table 2: Mendelian randomisation estimates of genetically predicted cardiovascular disease and risk of CHIP in IVW analyses

Exposure			CHIP Overall						DNMT3A CHIP						TET2 CHIP					
CVD	IEU GWAS ID	nsnp	Beta	SE	P	OR	LCI	UCI	Beta	SE	P	OR	LCI	UCI	Beta	SE	P	OR	LCI	UCI
Coronary artery disease	ebi-a-GCST003116	38	-0.027	0.022	0.206	0.973	0.932	1.015	-0.038	0.028	0.169	0.963	0.911	1.016	0.043	0.049	0.376	1.044	0.949	1.148
Atrial fibrillation	ebi-a-GCST006414	110	0.018	0.017	0.275	1.019	0.985	1.053	0.034	0.018	0.054	1.035	0.999	1.072	0.014	0.037	0.711	1.014	0.943	1.090
Stroke	ebi-a-GCST006906	7	-0.011	0.068	0.876	0.989	0.867	1.130	-0.023	0.076	0.766	0.978	0.843	1.134	0.099	0.151	0.514	1.104	0.821	1.484
Ischaemic stroke (large artery atherosclerosis)	ebi-a-GCST006907	3	0.008	0.040	0.838	1.008	0.932	1.090	0.002	0.045	0.958	1.002	0.918	1.094	0.017	0.089	0.850	1.017	0.854	1.212
Ischaemic stroke	ebi-a-GCST006908	8	0.044	0.057	0.441	1.045	0.934	1.169	-0.005	0.064	0.936	0.995	0.878	1.128	0.067	0.128	0.597	1.070	0.833	1.374
Cardioembolic Stroke	ebi-a-GCST006910	4	-0.025	0.031	0.416	0.975	0.919	1.036	0.002	0.034	0.956	1.002	0.937	1.071	-0.020	0.068	0.774	0.981	0.858	1.121
Heart failure	ebi-a-GCST009541	9	-0.125	0.069	0.068	0.882	0.771	1.009	-0.050	0.077	0.519	0.951	0.817	1.107	-0.339	0.153	0.027	0.713	0.528	0.962
Myocardial infarction	ieu-a-798	24	-0.022	0.026	0.402	0.978	0.929	1.030	-0.022	0.034	0.513	0.978	0.915	1.045	0.013	0.064	0.840	1.013	0.894	1.148

Abbreviations: nsnp, number of Single-nucleotide polymorphism; GWAS, Genome-wide association study; SE, standard error; P, P-value; OR, Odds ratio; LCI, lower 95% confidence interval of odds ratio; UCI, upper 95% confidence interval of odds ratio and CHIP, clonal hematopoiesis of indeterminate potential

Supplemental table 1: Mendelian randomisation estimates for genetically predicted cardiovascular diseases and risk of CHIP in supplemental analyses

ID-Exposure	ID-Outcome	Outcome	Exposure	Method	NSNP	Beta	SE	p	LCI	UCI	OR	OR-LCI	OR-UCI
ebi-a-GCST003116	RXUOs6	CHIP Overall	Coronary artery disease | | id:ebi-a-GCST003116	MR Egger	38	0.000344741	0.050341725	0.994573921	-0.098325041	0.099014523	1.000344801	0.906354254	1.104082334
ebi-a-GCST003116	RXUOs6	CHIP Overall	Coronary artery disease | | id:ebi-a-GCST003116	Weighted median	38	-0.046658455	0.033222804	0.160196653	-0.111775151	0.018458242	0.954413317	0.894245305	1.018629648
ebi-a-GCST003116	RXUOs6	CHIP Overall	Coronary artery disease | | id:ebi-a-GCST003116	Inverse variance weighted	38	-0.027468321	0.021713446	0.205857942	-0.070026676	0.015090034	0.972905503	0.932368948	1.015204464
ebi-a-GCST003116	RXUOs6	CHIP Overall	Coronary artery disease | | id:ebi-a-GCST003116	Simple mode	38	-0.080536025	0.063910095	0.215506901	-0.205799812	0.044727761	0.922621665	0.813996012	1.045743129
ebi-a-GCST003116	RXUOs6	CHIP Overall	Coronary artery disease | | id:ebi-a-GCST003116	Weighted mode	38	-0.052809888	0.042101372	0.217581118	-0.135328577	0.029708801	0.948560328	0.873428876	1.03015451
ebi-a-GCST006414	RXUOs6	CHIP Overall	Atrial fibrillation | | id:ebi-a-GCST006414	MR Egger	110	0.009477985	0.033064361	0.774927529	-0.055328162	0.074284132	1.009523043	0.946174598	1.077112804
ebi-a-GCST006414	RXUOs6	CHIP Overall	Atrial fibrillation | | id:ebi-a-GCST006414	Weighted median	110	-0.021070423	0.025084387	0.400918713	-0.070235823	0.028094976	0.979150007	0.932173966	1.028493362
ebi-a-GCST006414	RXUOs6	CHIP Overall	Atrial fibrillation | | id:ebi-a-GCST006414	Inverse variance weighted	110	0.018400717	0.016841538	0.274578515	-0.014608697	0.051410132	1.018571054	0.985497492	1.052754573
ebi-a-GCST006414	RXUOs6	CHIP Overall	Atrial fibrillation | | id:ebi-a-GCST006414	Simple mode	110	0.020724902	0.048399319	0.669345724	-0.074137762	0.115587567	1.020941155	0.928543767	1.122532807
ebi-a-GCST006414	RXUOs6	CHIP Overall	Atrial fibrillation | | id:ebi-a-GCST006414	Weighted mode	110	-0.011067502	0.025756104	0.668259208	-0.061549465	0.039414462	0.988993518	0.940306432	1.040201518
ebi-a-GCST006906	RXUOs6	CHIP Overall	Stroke | | id:ebi-a-GCST006906	MR Egger	7	-0.174457998	0.441821591	0.709227323	-1.040428316	0.69151232	0.839912131	0.353303324	1.99673295
ebi-a-GCST006906	RXUOs6	CHIP Overall	Stroke | | id:ebi-a-GCST006906	Weighted median	7	-0.031673463	0.088211777	0.719549001	-0.204568546	0.141221621	0.968822887	0.814998875	1.151679856
ebi-a-GCST006906	RXUOs6	CHIP Overall	Stroke | | id:ebi-a-GCST006906	Inverse variance weighted	7	-0.01059699	0.067691559	0.875600949	-0.143272445	0.122078465	0.98945896	0.866517958	1.129842752
ebi-a-GCST006906	RXUOs6	CHIP Overall	Stroke | | id:ebi-a-GCST006906	Simple mode	7	-0.114992091	0.135084063	0.427285125	-0.379756855	0.149772673	0.891373194	0.684027707	1.161570157
ebi-a-GCST006906	RXUOs6	CHIP Overall	Stroke | | id:ebi-a-GCST006906	Weighted mode	7	-0.113259905	0.135569091	0.435468377	-0.378975324	0.152455514	0.892918556	0.684562505	1.164690649
ebi-a-GCST006907	RXUOs6	CHIP Overall	Ischemic stroke (large artery atherosclerosis) | | id:ebi-a-GCST006907	MR Egger	3	-0.300196128	0.355372585	0.553455617	-0.996726394	0.396334138	0.74067294	0.369085707	1.486365886
ebi-a-GCST006907	RXUOs6	CHIP Overall	Ischemic stroke (large artery atherosclerosis) | | id:ebi-a-GCST006907	Weighted median	3	-0.012716182	0.047617427	0.789431497	-0.10604634	0.080613975	0.987364327	0.89938297	1.083952383
ebi-a-GCST006907	RXUOs6	CHIP Overall	Ischemic stroke (large artery atherosclerosis) | | id:ebi-a-GCST006907	Inverse variance weighted	3	0.00818117	0.040007991	0.837971858	-0.070234493	0.086596833	1.008214727	0.932175206	1.090456955
ebi-a-GCST006907	RXUOs6	CHIP Overall	Ischemic stroke (large artery atherosclerosis) | | id:ebi-a-GCST006907	Simple mode	3	-0.02389794	0.06007714	0.729229187	-0.141649134	0.093853255	0.976385355	0.867925729	1.098398549
ebi-a-GCST006907	RXUOs6	CHIP Overall	Ischemic stroke (large artery atherosclerosis) | | id:ebi-a-GCST006907	Weighted mode	3	-0.021380662	0.056319617	0.740739134	-0.131767111	0.089005786	0.978846284	0.876545109	1.093086981
ebi-a-GCST006908	RXUOs6	CHIP Overall	Ischemic stroke | | id:ebi-a-GCST006908	MR Egger	8	-0.579997273	0.417227724	0.213870102	-1.397763611	0.237769065	0.559899893	0.247149068	1.268416237
ebi-a-GCST006908	RXUOs6	CHIP Overall	Ischemic stroke | | id:ebi-a-GCST006908	Weighted median	8	0.027584655	0.074652976	0.711751279	-0.118735179	0.173904488	1.027968634	0.888042942	1.189941907
ebi-a-GCST006908	RXUOs6	CHIP Overall	Ischemic stroke | | id:ebi-a-GCST006908	Inverse variance weighted	8	0.044048749	0.057156868	0.44090602	-0.067978711	0.15607621	1.045033298	0.934280363	1.168915282
ebi-a-GCST006908	RXUOs6	CHIP Overall	Ischemic stroke | | id:ebi-a-GCST006908	Simple mode	8	-0.052675762	0.121029867	0.676499664	-0.289894301	0.184542777	0.948687563	0.748342663	1.202668426
ebi-a-GCST006908	RXUOs6	CHIP Overall	Ischemic stroke | | id:ebi-a-GCST006908	Weighted mode	8	-0.059785965	0.123949777	0.644281224	-0.302727528	0.183155598	0.941966126	0.738800371	1.201001267
ebi-a-GCST006910	RXUOs6	CHIP Overall	Ischemic stroke (cardioembolic) | | id:ebi-a-GCST006910	MR Egger	4	0.024378498	0.060438168	0.725717965	-0.094080312	0.142837307	1.024678083	0.910209659	1.153542113
ebi-a-GCST006910	RXUOs6	CHIP Overall	Ischemic stroke (cardioembolic) | | id:ebi-a-GCST006910	Weighted median	4	-0.014335425	0.03656044	0.694982785	-0.085993886	0.057323037	0.985766838	0.917599841	1.058997851
ebi-a-GCST006910	RXUOs6	CHIP Overall	Ischemic stroke (cardioembolic) | | id:ebi-a-GCST006910	Inverse variance weighted	4	-0.024850011	0.030546578	0.415924518	-0.084721305	0.035021283	0.975456209	0.918768305	1.03564175
ebi-a-GCST006910	RXUOs6	CHIP Overall	Ischemic stroke (cardioembolic) | | id:ebi-a-GCST006910	Simple mode	4	0.010517807	0.048318065	0.84164469	-0.084185599	0.105221214	1.010573314	0.919260626	1.110956343
ebi-a-GCST006910	RXUOs6	CHIP Overall	Ischemic stroke (cardioembolic) | | id:ebi-a-GCST006910	Weighted mode	4	-0.02627849	0.038921719	0.547952227	-0.10256506	0.05000808	0.974063785	0.90251943	1.05127959
ebi-a-GCST009541	RXUOs6	CHIP Overall	Heart failure | | id:ebi-a-GCST009541	MR Egger	9	-0.039595374	0.198592123	0.847633126	-0.428835934	0.349645187	0.961178278	0.651266771	1.418564134
ebi-a-GCST009541	RXUOs6	CHIP Overall	Heart failure | | id:ebi-a-GCST009541	Weighted median	9	-0.119163991	0.092670458	0.198481543	-0.300798088	0.062470106	0.88766222	0.740227218	1.064462637
ebi-a-GCST009541	RXUOs6	CHIP Overall	Heart failure | | id:ebi-a-GCST009541	Inverse variance weighted	9	-0.125247008	0.06866854	0.068161781	-0.259837347	0.009343331	0.882278946	0.77117701	1.009387116
ebi-a-GCST009541	RXUOs6	CHIP Overall	Heart failure | | id:ebi-a-GCST009541	Simple mode	9	-0.237854849	0.149845703	0.151099367	-0.531552428	0.055842729	0.788317108	0.587691912	1.057431368
ebi-a-GCST009541	RXUOs6	CHIP Overall	Heart failure | | id:ebi-a-GCST009541	Weighted mode	9	-0.146007926	0.125067662	0.276651288	-0.391140543	0.09912469	0.864150854	0.676285102	1.104203975
ieu-a-798	RXUOs6	CHIP Overall	Myocardial infarction | | id:ieu-a-798	MR Egger	24	-0.045596112	0.063845038	0.482632214	-0.170732387	0.079540164	0.95542777	0.843047154	1.082789048
ieu-a-798	RXUOs6	CHIP Overall	Myocardial infarction | | id:ieu-a-798	Weighted median	24	-0.044908722	0.036914618	0.223772961	-0.117261374	0.027443929	0.956084747	0.889352709	1.027823982
ieu-a-798	RXUOs6	CHIP Overall	Myocardial infarction | | id:ieu-a-798	Inverse variance weighted	24	-0.022114724	0.026363178	0.401554079	-0.073786554	0.029557105	0.978128013	0.928869936	1.029998252
ieu-a-798	RXUOs6	CHIP Overall	Myocardial infarction | | id:ieu-a-798	Simple mode	24	-0.049855656	0.065209405	0.452314714	-0.17766609	0.077954778	0.951366739	0.837221933	1.08107377
ieu-a-798	RXUOs6	CHIP Overall	Myocardial infarction | | id:ieu-a-798	Weighted mode	24	-0.049855656	0.044548821	0.274639222	-0.137171346	0.037460034	0.951366739	0.871820831	1.038170505
ebi-a-GCST003116	fmbgiS	DNMT3A CHIP	Coronary artery disease | | id:ebi-a-GCST003116	MR Egger	38	0.007012997	0.064765768	0.914372908	-0.119927908	0.133953903	1.007037646	0.886984379	1.143340114
ebi-a-GCST003116	fmbgiS	DNMT3A CHIP	Coronary artery disease | | id:ebi-a-GCST003116	Weighted median	38	-0.050502374	0.038537192	0.190032148	-0.126035271	0.025030522	0.950751671	0.881583752	1.025346416
ebi-a-GCST003116	fmbgiS	DNMT3A CHIP	Coronary artery disease | | id:ebi-a-GCST003116	Inverse variance weighted	38	-0.038210315	0.027784884	0.169063404	-0.092668688	0.016248059	0.962510489	0.91149544	1.016380776
ebi-a-GCST003116	fmbgiS	DNMT3A CHIP	Coronary artery disease | | id:ebi-a-GCST003116	Simple mode	38	-0.036199369	0.068402459	0.59981966	-0.170268189	0.09786945	0.964447993	0.843438585	1.102818803
ebi-a-GCST003116	fmbgiS	DNMT3A CHIP	Coronary artery disease | | id:ebi-a-GCST003116	Weighted mode	38	-0.044583817	0.045657469	0.335165925	-0.134072456	0.044904822	0.956395435	0.874526698	1.045928306
ebi-a-GCST006414	fmbgiS	DNMT3A CHIP	Atrial fibrillation | | id:ebi-a-GCST006414	MR Egger	110	0.043851538	0.034956616	0.212384424	-0.024663429	0.112366506	1.044827227	0.975638228	1.118922878
ebi-a-GCST006414	fmbgiS	DNMT3A CHIP	Atrial fibrillation | | id:ebi-a-GCST006414	Weighted median	110	0.007737871	0.026626459	0.771350876	-0.044449989	0.059925732	1.007767886	0.956523435	1.061757689
ebi-a-GCST006414	fmbgiS	DNMT3A CHIP	Atrial fibrillation | | id:ebi-a-GCST006414	Inverse variance weighted	110	0.034367661	0.017806093	0.05359361	-0.000532282	0.069267605	1.034965054	0.99946786	1.071722969
ebi-a-GCST006414	fmbgiS	DNMT3A CHIP	Atrial fibrillation | | id:ebi-a-GCST006414	Simple mode	110	0.05341308	0.055046724	0.334033963	-0.054478498	0.161304659	1.054865299	0.94697887	1.175042902
ebi-a-GCST006414	fmbgiS	DNMT3A CHIP	Atrial fibrillation | | id:ebi-a-GCST006414	Weighted mode	110	0.012342331	0.029485157	0.676336003	-0.045448578	0.070133239	1.012418811	0.955568739	1.072651091
ebi-a-GCST006906	fmbgiS	DNMT3A CHIP	Stroke | | id:ebi-a-GCST006906	MR Egger	7	-0.243082803	0.494428687	0.64379221	-1.212163029	0.725997423	0.784206577	0.297552967	2.066791538
ebi-a-GCST006906	fmbgiS	DNMT3A CHIP	Stroke | | id:ebi-a-GCST006906	Weighted median	7	-0.073932138	0.094782365	0.43537912	-0.259705573	0.111841298	0.928734718	0.771278638	1.118335364
ebi-a-GCST006906	fmbgiS	DNMT3A CHIP	Stroke | | id:ebi-a-GCST006906	Inverse variance weighted	7	-0.022562145	0.075758637	0.765843489	-0.171049074	0.125924783	0.977690476	0.842780214	1.134196854
ebi-a-GCST006906	fmbgiS	DNMT3A CHIP	Stroke | | id:ebi-a-GCST006906	Simple mode	7	-0.085900493	0.137121979	0.554088282	-0.354659572	0.182858586	0.917685543	0.701412183	1.200644608
ebi-a-GCST006906	fmbgiS	DNMT3A CHIP	Stroke | | id:ebi-a-GCST006906	Weighted mode	7	-0.089619064	0.137362106	0.538301779	-0.358848792	0.179610664	0.914279401	0.698479959	1.196751334
ebi-a-GCST006907	fmbgiS	DNMT3A CHIP	Ischemic stroke (large artery atherosclerosis) | | id:ebi-a-GCST006907	MR Egger	3	-0.263851731	0.397742567	0.627119707	-1.043427163	0.515723701	0.768087415	0.352245408	1.674850154
ebi-a-GCST006907	fmbgiS	DNMT3A CHIP	Ischemic stroke (large artery atherosclerosis) | | id:ebi-a-GCST006907	Weighted median	3	-0.017925428	0.049324505	0.716292436	-0.114601458	0.078750602	0.982234277	0.891721462	1.081934457
ebi-a-GCST006907	fmbgiS	DNMT3A CHIP	Ischemic stroke (large artery atherosclerosis) | | id:ebi-a-GCST006907	Inverse variance weighted	3	0.002350025	0.044771755	0.958139048	-0.085402615	0.090102666	1.002352789	0.918142552	1.094286624
ebi-a-GCST006907	fmbgiS	DNMT3A CHIP	Ischemic stroke (large artery atherosclerosis) | | id:ebi-a-GCST006907	Simple mode	3	-0.021532394	0.063042088	0.765233497	-0.145094886	0.102030098	0.978697773	0.864940219	1.107416803
ebi-a-GCST006907	fmbgiS	DNMT3A CHIP	Ischemic stroke (large artery atherosclerosis) | | id:ebi-a-GCST006907	Weighted mode	3	-0.021361122	0.065529794	0.775389913	-0.149799518	0.107077274	0.978865411	0.86088055	1.113020258
ebi-a-GCST006908	fmbgiS	DNMT3A CHIP	Ischemic stroke | | id:ebi-a-GCST006908	MR Egger	8	-0.385244375	0.467087083	0.441045658	-1.300735057	0.530246308	0.680284372	0.27233154	1.69935082
ebi-a-GCST006908	fmbgiS	DNMT3A CHIP	Ischemic stroke | | id:ebi-a-GCST006908	Weighted median	8	-0.04224242	0.081504216	0.604259377	-0.201990683	0.117505844	0.95863736	0.817102541	1.124688203
ebi-a-GCST006908	fmbgiS	DNMT3A CHIP	Ischemic stroke | | id:ebi-a-GCST006908	Inverse variance weighted	8	-0.005147981	0.063984903	0.935874442	-0.130558391	0.120262428	0.994865247	0.877605248	1.127792777
ebi-a-GCST006908	fmbgiS	DNMT3A CHIP	Ischemic stroke | | id:ebi-a-GCST006908	Simple mode	8	-0.056971646	0.122566378	0.656170669	-0.297201747	0.183258455	0.944620853	0.742894121	1.201124805
ebi-a-GCST006908	fmbgiS	DNMT3A CHIP	Ischemic stroke | | id:ebi-a-GCST006908	Weighted mode	8	-0.053254347	0.117601933	0.664368891	-0.283754137	0.177245442	0.948138825	0.752951745	1.193924096
ebi-a-GCST006910	fmbgiS	DNMT3A CHIP	Ischemic stroke (cardioembolic) | | id:ebi-a-GCST006910	MR Egger	4	0.001470561	0.067703818	0.984643095	-0.131228922	0.134170045	1.001471643	0.877016983	1.143587264
ebi-a-GCST006910	fmbgiS	DNMT3A CHIP	Ischemic stroke (cardioembolic) | | id:ebi-a-GCST006910	Weighted median	4	-0.006490721	0.040601002	0.872986598	-0.086068685	0.073087242	0.993530298	0.917531209	1.07582439
ebi-a-GCST006910	fmbgiS	DNMT3A CHIP	Ischemic stroke (cardioembolic) | | id:ebi-a-GCST006910	Inverse variance weighted	4	0.001871201	0.034198494	0.956364817	-0.065157847	0.068900249	1.001872952	0.936919561	1.071329337
ebi-a-GCST006910	fmbgiS	DNMT3A CHIP	Ischemic stroke (cardioembolic) | | id:ebi-a-GCST006910	Simple mode	4	0.031387422	0.058241348	0.627365868	-0.08276562	0.145540464	1.031885202	0.920566885	1.156664537
ebi-a-GCST006910	fmbgiS	DNMT3A CHIP	Ischemic stroke (cardioembolic) | | id:ebi-a-GCST006910	Weighted mode	4	-0.023651494	0.044113432	0.629058913	-0.110113822	0.062810833	0.97662601	0.895732176	1.06482539
ebi-a-GCST009541	fmbgiS	DNMT3A CHIP	Heart failure | | id:ebi-a-GCST009541	MR Egger	9	0.066959952	0.234648765	0.783625376	-0.392951626	0.526871531	1.069252656	0.675061402	1.693625557
ebi-a-GCST009541	fmbgiS	DNMT3A CHIP	Heart failure | | id:ebi-a-GCST009541	Weighted median	9	-0.051247212	0.097601144	0.599536101	-0.242545455	0.14005103	0.950043779	0.784628082	1.150332499
ebi-a-GCST009541	fmbgiS	DNMT3A CHIP	Heart failure | | id:ebi-a-GCST009541	Inverse variance weighted	9	-0.049922327	0.077410695	0.518990574	-0.201647289	0.101802636	0.951303312	0.817383177	1.107164936
ebi-a-GCST009541	fmbgiS	DNMT3A CHIP	Heart failure | | id:ebi-a-GCST009541	Simple mode	9	-0.164117399	0.168307428	0.35806744	-0.493999959	0.16576516	0.848642386	0.610180808	1.180295889
ebi-a-GCST009541	fmbgiS	DNMT3A CHIP	Heart failure | | id:ebi-a-GCST009541	Weighted mode	9	-0.126608221	0.147801275	0.416579541	-0.41629872	0.163082278	0.881078794	0.659483241	1.177133538
ieu-a-798	fmbgiS	DNMT3A CHIP	Myocardial infarction | | id:ieu-a-798	MR Egger	24	-0.050431326	0.082288476	0.54625267	-0.21171674	0.110854087	0.950819223	0.809193878	1.117231876
ieu-a-798	fmbgiS	DNMT3A CHIP	Myocardial infarction | | id:ieu-a-798	Weighted median	24	-0.047920563	0.041267296	0.245550934	-0.128804464	0.032963338	0.953209504	0.879145853	1.033512648
ieu-a-798	fmbgiS	DNMT3A CHIP	Myocardial infarction | | id:ieu-a-798	Inverse variance weighted	24	-0.022233251	0.033966503	0.5127485	-0.088807597	0.044341096	0.978012086	0.915021609	1.045338855
ieu-a-798	fmbgiS	DNMT3A CHIP	Myocardial infarction | | id:ieu-a-798	Simple mode	24	-0.000473949	0.069677391	0.994631446	-0.137041635	0.136093737	0.999526163	0.871933922	1.145789291
ieu-a-798	fmbgiS	DNMT3A CHIP	Myocardial infarction | | id:ieu-a-798	Weighted mode	24	-0.032927069	0.043851225	0.460338052	-0.11887547	0.053021333	0.967609126	0.887918366	1.054452139
ebi-a-GCST003116	ZK7HIF	TET2 CHIP	Coronary artery disease | | id:ebi-a-GCST003116	MR Egger	38	0.005734465	0.113945451	0.960140484	-0.21759862	0.229067549	1.005750938	0.804448266	1.257426975
ebi-a-GCST003116	ZK7HIF	TET2 CHIP	Coronary artery disease | | id:ebi-a-GCST003116	Weighted median	38	0.022765036	0.077282216	0.768322253	-0.128708108	0.174238179	1.023026136	0.879230568	1.190339046
ebi-a-GCST003116	ZK7HIF	TET2 CHIP	Coronary artery disease | | id:ebi-a-GCST003116	Inverse variance weighted	38	0.043020994	0.048592385	0.375970933	-0.052220081	0.138262068	1.043959811	0.949119961	1.148276438
ebi-a-GCST003116	ZK7HIF	TET2 CHIP	Coronary artery disease | | id:ebi-a-GCST003116	Simple mode	38	0.000832729	0.159419876	0.995860336	-0.311630229	0.313295686	1.000833076	0.732252244	1.367925948
ebi-a-GCST003116	ZK7HIF	TET2 CHIP	Coronary artery disease | | id:ebi-a-GCST003116	Weighted mode	38	-0.010248189	0.135392387	0.940071534	-0.275617267	0.255120888	0.989804145	0.759103409	1.290617632
ebi-a-GCST006414	ZK7HIF	TET2 CHIP	Atrial fibrillation | | id:ebi-a-GCST006414	MR Egger	110	0.017084837	0.072609072	0.814422308	-0.125228944	0.159398617	1.017231617	0.882294884	1.172805354
ebi-a-GCST006414	ZK7HIF	TET2 CHIP	Atrial fibrillation | | id:ebi-a-GCST006414	Weighted median	110	0.02762418	0.062065182	0.656259512	-0.094023577	0.149271937	1.028009265	0.9102613	1.160988663
ebi-a-GCST006414	ZK7HIF	TET2 CHIP	Atrial fibrillation | | id:ebi-a-GCST006414	Inverse variance weighted	110	0.013718792	0.036977931	0.710637937	-0.058757952	0.086195537	1.013813327	0.942934977	1.090019446
ebi-a-GCST006414	ZK7HIF	TET2 CHIP	Atrial fibrillation | | id:ebi-a-GCST006414	Simple mode	110	-0.103850787	0.124474943	0.405930632	-0.347821675	0.140120101	0.901359782	0.706224802	1.150411956
ebi-a-GCST006414	ZK7HIF	TET2 CHIP	Atrial fibrillation | | id:ebi-a-GCST006414	Weighted mode	110	-0.045181219	0.064255407	0.483461153	-0.171121816	0.080759378	0.955824252	0.842718911	1.084110004
ebi-a-GCST006906	ZK7HIF	TET2 CHIP	Stroke | | id:ebi-a-GCST006906	MR Egger	7	-1.35435397	0.986342644	0.228097534	-3.287585553	0.578877612	0.25811399	0.037343906	1.784034927
ebi-a-GCST006906	ZK7HIF	TET2 CHIP	Stroke | | id:ebi-a-GCST006906	Weighted median	7	0.006785781	0.199875046	0.972916928	-0.384969309	0.398540872	1.006808857	0.68047152	1.489649521
ebi-a-GCST006906	ZK7HIF	TET2 CHIP	Stroke | | id:ebi-a-GCST006906	Inverse variance weighted	7	0.098578865	0.151203954	0.514427253	-0.197780884	0.394938614	1.103601436	0.820549629	1.484293073
ebi-a-GCST006906	ZK7HIF	TET2 CHIP	Stroke | | id:ebi-a-GCST006906	Simple mode	7	-0.024627582	0.269185589	0.930081915	-0.552231337	0.502976174	0.975673203	0.575663876	1.653635461
ebi-a-GCST006906	ZK7HIF	TET2 CHIP	Stroke | | id:ebi-a-GCST006906	Weighted mode	7	-0.015193816	0.254473211	0.954328052	-0.513961309	0.483573677	0.984921028	0.598121536	1.621860061
ebi-a-GCST006907	ZK7HIF	TET2 CHIP	Ischemic stroke (large artery atherosclerosis) | | id:ebi-a-GCST006907	MR Egger	3	-0.536040076	0.91952429	0.663997723	-2.338307685	1.266227533	0.585060466	0.096490793	3.54744467
ebi-a-GCST006907	ZK7HIF	TET2 CHIP	Ischemic stroke (large artery atherosclerosis) | | id:ebi-a-GCST006907	Weighted median	3	0.001799315	0.112007074	0.987183095	-0.217734549	0.221333179	1.001800935	0.804338926	1.247739082
ebi-a-GCST006907	ZK7HIF	TET2 CHIP	Ischemic stroke (large artery atherosclerosis) | | id:ebi-a-GCST006907	Inverse variance weighted	3	0.016938751	0.089393066	0.84971178	-0.158271658	0.19214916	1.017083025	0.853617858	1.211851264
ebi-a-GCST006907	ZK7HIF	TET2 CHIP	Ischemic stroke (large artery atherosclerosis) | | id:ebi-a-GCST006907	Simple mode	3	-0.040001836	0.145608897	0.809307502	-0.325395274	0.245391602	0.960787675	0.722241814	1.27812173
ebi-a-GCST006907	ZK7HIF	TET2 CHIP	Ischemic stroke (large artery atherosclerosis) | | id:ebi-a-GCST006907	Weighted mode	3	-0.018249213	0.132277828	0.902907574	-0.277513755	0.241015329	0.981916295	0.757665143	1.272540542
ebi-a-GCST006908	ZK7HIF	TET2 CHIP	Ischemic stroke | | id:ebi-a-GCST006908	MR Egger	8	-0.64009585	0.958102786	0.528925427	-2.51797731	1.23778561	0.527241886	0.080622516	3.447969856
ebi-a-GCST006908	ZK7HIF	TET2 CHIP	Ischemic stroke | | id:ebi-a-GCST006908	Weighted median	8	-0.011654521	0.162930804	0.942975558	-0.330998896	0.307689855	0.98841313	0.718205962	1.36027904
ebi-a-GCST006908	ZK7HIF	TET2 CHIP	Ischemic stroke | | id:ebi-a-GCST006908	Inverse variance weighted	8	0.067487752	0.127534183	0.596684869	-0.182479247	0.317454752	1.069817157	0.833201935	1.373627089
ebi-a-GCST006908	ZK7HIF	TET2 CHIP	Ischemic stroke | | id:ebi-a-GCST006908	Simple mode	8	-0.031384027	0.222929721	0.892009351	-0.46832628	0.405558227	0.96910334	0.626049223	1.500139685
ebi-a-GCST006908	ZK7HIF	TET2 CHIP	Ischemic stroke | | id:ebi-a-GCST006908	Weighted mode	8	-0.003193072	0.219070635	0.988777541	-0.432571516	0.426185372	0.996812021	0.648838449	1.531404628
ebi-a-GCST006910	ZK7HIF	TET2 CHIP	Ischemic stroke (cardioembolic) | | id:ebi-a-GCST006910	MR Egger	4	0.114966507	0.13514849	0.484551131	-0.149924534	0.379857548	1.121835864	0.860772933	1.4620763
ebi-a-GCST006910	ZK7HIF	TET2 CHIP	Ischemic stroke (cardioembolic) | | id:ebi-a-GCST006910	Weighted median	4	0.017532344	0.077303803	0.820580861	-0.133983111	0.169047798	1.017686937	0.874604836	1.184176739
ebi-a-GCST006910	ZK7HIF	TET2 CHIP	Ischemic stroke (cardioembolic) | | id:ebi-a-GCST006910	Inverse variance weighted	4	-0.01961091	0.06825902	0.773881573	-0.15339859	0.11417677	0.980580133	0.857787748	1.120950257
ebi-a-GCST006910	ZK7HIF	TET2 CHIP	Ischemic stroke (cardioembolic) | | id:ebi-a-GCST006910	Simple mode	4	0.011756188	0.107724251	0.919987897	-0.199383344	0.222895719	1.011825563	0.819235784	1.249690248
ebi-a-GCST006910	ZK7HIF	TET2 CHIP	Ischemic stroke (cardioembolic) | | id:ebi-a-GCST006910	Weighted mode	4	0.01724803	0.089638299	0.859703478	-0.158443036	0.192939097	1.017397636	0.853471579	1.212808927
ebi-a-GCST009541	ZK7HIF	TET2 CHIP	Heart failure | | id:ebi-a-GCST009541	MR Egger	9	0.012922384	0.451037347	0.977943158	-0.871110816	0.896955584	1.013006239	0.41848643	2.452126444
ebi-a-GCST009541	ZK7HIF	TET2 CHIP	Heart failure | | id:ebi-a-GCST009541	Weighted median	9	-0.249080696	0.199748282	0.212407432	-0.64058733	0.142425937	0.779517067	0.52698282	1.153067678
ebi-a-GCST009541	ZK7HIF	TET2 CHIP	Heart failure | | id:ebi-a-GCST009541	Inverse variance weighted	9	-0.338845316	0.153229168	0.027010709	-0.639174485	-0.038516146	0.712592667	0.527727891	0.962216169
ebi-a-GCST009541	ZK7HIF	TET2 CHIP	Heart failure | | id:ebi-a-GCST009541	Simple mode	9	-0.273513	0.293792057	0.379103962	-0.849345433	0.302319433	0.760702447	0.427694795	1.352993348
ebi-a-GCST009541	ZK7HIF	TET2 CHIP	Heart failure | | id:ebi-a-GCST009541	Weighted mode	9	-0.269075869	0.278138582	0.361668175	-0.814227491	0.276075753	0.764085283	0.442981403	1.317947698
ieu-a-798	ZK7HIF	TET2 CHIP	Myocardial infarction | | id:ieu-a-798	MR Egger	24	0.014210258	0.154943937	0.92775664	-0.289479858	0.317900375	1.014311704	0.748652872	1.374239345
ieu-a-798	ZK7HIF	TET2 CHIP	Myocardial infarction | | id:ieu-a-798	Weighted median	24	0.021615624	0.087427839	0.804722796	-0.149742939	0.192974188	1.021850934	0.860929259	1.212851487
ieu-a-798	ZK7HIF	TET2 CHIP	Myocardial infarction | | id:ieu-a-798	Inverse variance weighted	24	0.012838574	0.063742177	0.840374785	-0.112096093	0.13777324	1.012921342	0.89395835	1.147715265
ieu-a-798	ZK7HIF	TET2 CHIP	Myocardial infarction | | id:ieu-a-798	Simple mode	24	-0.114005302	0.176987143	0.525854007	-0.460900102	0.232889497	0.892253225	0.630715682	1.262241991
ieu-a-798	ZK7HIF	TET2 CHIP	Myocardial infarction | | id:ieu-a-798	Weighted mode	24	0.148824666	0.138876061	0.294999873	-0.123372413	0.421021744	1.160469501	0.883934413	1.523517406

Abbreviations: NSNP, number of single nucleotide polymorphisms; SE, standard error; p, p-value; LCI, lower confidence interval (CI); UCI, upper CI; OR, odds ratio.

Supplemental table 4: Characteristics of instrumental variables for circulating cytokines and their associations with CHIP

Cytokine info		SNP info						SNP-cytokine					
Cytokine	Gene	SNP	IV Source	IV_type	CHR	Pos: grch37	Pos: grch38	EA	OA	EAF	BETA	SE	Unit
C-reactive protein	CRP	rs149327578	Yarmolinsky(PMID:37205426)	cis-pQTL	1	159576889	159607099	G	A	0.825	0.094	0.016	sd
C-reactive protein	CRP	rs4656241	Yarmolinsky(PMID:37205426)	cis-pQTL	1	159613986	159644196	T	C	0.796	-0.112	0.015	sd
C-reactive protein	CRP	rs77013776	Yarmolinsky(PMID:37205426)	cis-pQTL	1	159652068	159682278	A	C	0.964	0.245	0.033	sd
C-reactive protein	CRP	rs3093059	Yarmolinsky(PMID:37205426)	cis-pQTL	1	159685136	159715346	A	G	0.938	-0.246	0.024	sd
C-reactive protein	CRP	rs4546916	Yarmolinsky(PMID:37205426)	cis-pQTL	1	159699249	159729459	G	T	0.673	0.179	0.013	sd
Interleukin 1 receptor accessary protein	IL1Racp	rs6444430	Sun(PMID:29875488)	cis-pQTL	3	190223191	190505402	A	G	0.790	0.154	0.033	sd of log
Interleukin 1 receptor accessary protein	IL1Racp	rs2885370	Sun(PMID:29875488)	cis-pQTL	3	190276995	190559206	T	G	0.743	-0.209	0.031	sd of log
Interleukin 1 receptor accessary protein	IL1Racp	rs6801017	Sun(PMID:29875488)	cis-pQTL	3	190295730	190577941	G	A	0.214	-0.202	0.031	sd of log
Interleukin 1 receptor accessary protein	IL1Racp	rs67249092	Sun(PMID:29875488)	cis-pQTL	3	190357571	190639782	G	A	0.152	-0.379	0.035	sd of log
Interleukin 1 receptor accessary protein	IL1Racp	rs3935774	Sun(PMID:29875488)	cis-pQTL	3	190360571	190642782	T	C	0.017	0.741	0.112	sd of log
Interleukin 1 receptor accessary protein	IL1Racp	rs113553332	Sun(PMID:29875488)	cis-pQTL	3	190521288	190803499	T	C	0.048	0.685	0.056	sd of log
Interleukin 1 receptor antagonist	IL1RA	rs9308676	Karhunen(PMID:36936266)	cis-pQTL	2	113428167	112670590	G	A	0.555	-0.043	0.011	sd
Interleukin 1 receptor antagonist	IL1RA	rs7594852	Karhunen(PMID:36936266)	cis-pQTL	2	113521754	112764177	C	T	0.522	-0.045	0.010	sd
Interleukin 1 receptor antagonist	IL1RA	rs3783548	Karhunen(PMID:36936266)	cis-pQTL	2	113533333	112775756	G	A	0.096	-0.086	0.021	sd
Interleukin 1 receptor antagonist	IL1RA	rs1143636	Karhunen(PMID:36936266)	cis-pQTL	2	113589374	112831797	A	G	0.975	0.242	0.038	sd
Interleukin 1 receptor antagonist	IL1RA	rs148433212	Karhunen(PMID:36936266)	cis-pQTL	2	113616075	112858498	G	T	0.965	-0.199	0.039	sd
Interleukin 1 receptor antagonist	IL1RA	rs4591347	Karhunen(PMID:36936266)	cis-pQTL	2	113658705	112901128	C	T	0.865	0.101	0.015	sd
Interleukin 1 receptor antagonist	IL1RA	rs7584409	Karhunen(PMID:36936266)	cis-pQTL	2	113742734	112985157	G	A	0.288	0.086	0.012	sd
Interleukin 1 receptor antagonist	IL1RA	rs115652897	Karhunen(PMID:36936266)	cis-pQTL	2	113790332	113032755	C	T	0.981	0.251	0.043	sd
Interleukin 1 receptor antagonist	IL1RA	rs114189538	Karhunen(PMID:36936266)	cis-pQTL	2	113821082	113063505	G	A	0.934	0.167	0.022	sd
Interleukin 1 receptor antagonist	IL1RA	rs115853107	Karhunen(PMID:36936266)	cis-pQTL	2	113832153	113074576	A	C	0.988	-0.312	0.057	sd
Interleukin 1 receptor antagonist	IL1RA	rs6743376	Karhunen(PMID:36936266)	cis-pQTL	2	113832333	113074756	C	A	0.353	0.182	0.011	sd
Interleukin 1 receptor antagonist	IL1RA	rs139076984	Karhunen(PMID:36936266)	cis-pQTL	2	113839374	113081797	T	G	0.979	0.230	0.040	sd
Interleukin 1 receptor antagonist	IL1RA	rs6734238	Karhunen(PMID:36936266)	cis-pQTL	2	113841030	113083453	A	G	0.612	0.204	0.010	sd
Interleukin 1 receptor antagonist	IL1RA	rs146220584	Karhunen(PMID:36936266)	cis-pQTL	2	113843626	113086049	T	G	0.989	0.281	0.058	sd
Interleukin 1 receptor antagonist	IL1RA	rs72827900	Karhunen(PMID:36936266)	cis-pQTL	2	113858601	113101024	C	T	0.955	-0.112	0.028	sd
Interleukin 1 receptor antagonist	IL1RA	rs6723639	Karhunen(PMID:36936266)	cis-pQTL	2	113872238	113114661	C	T	0.793	0.066	0.013	sd
Interleukin 1 receptor antagonist	IL1RA	rs74667587	Karhunen(PMID:36936266)	cis-pQTL	2	113895079	113137502	G	A	0.819	0.149	0.014	sd
Interleukin 1 receptor antagonist	IL1RA	rs72829856	Karhunen(PMID:36936266)	cis-pQTL	2	113913287	113155710	G	A	0.905	-0.213	0.020	sd
Interleukin 1 receptor antagonist	IL1RA	rs45592842	Karhunen(PMID:36936266)	cis-pQTL	2	113943063	113185486	T	G	0.914	0.095	0.023	sd
Interleukin 1 receptor antagonist	IL1RA	rs72831808	Karhunen(PMID:36936266)	cis-pQTL	2	113979355	113221778	G	C	0.940	-0.124	0.025	sd
Interleukin 1 receptor antagonist	IL1RA	rs1491585	Karhunen(PMID:36936266)	cis-pQTL	2	114003621	113246044	A	G	0.525	-0.075	0.011	sd
Interleukin 1 receptor antagonist	IL1RA	rs1191686	Karhunen(PMID:36936266)	cis-pQTL	2	114094703	113337126	T	C	0.187	-0.072	0.013	sd
Interleukin 1 receptor antagonist	IL1RA	rs1867868	Karhunen(PMID:36936266)	cis-pQTL	2	114162983	113405406	T	C	0.491	0.063	0.011	sd
Interleukin 1 receptor type 1	IL1R1	rs719249	Yarmolinsky(PMID:37205426)	cis-pQTL	2	102623885	102007423	A	G	0.891	-0.104	0.018	sd
Interleukin 1 receptor type 1	IL1R1	rs11888059	Yarmolinsky(PMID:37205426)	cis-pQTL	2	102665086	102048625	A	G	0.497	-0.099	0.011	sd
Interleukin 1 receptor type 1	IL1R1	rs1030022	Yarmolinsky(PMID:37205426)	cis-pQTL	2	102732369	102115909	T	G	0.690	0.130	0.012	sd
Interleukin 1 receptor type 1	IL1R1	rs3755295	Yarmolinsky(PMID:37205426)	cis-pQTL	2	102768294	102151834	G	A	0.363	-0.073	0.012	sd
Interleukin 1 receptor type 1	IL1R1	rs3732131	Yarmolinsky(PMID:37205426)	cis-pQTL	2	102794603	102178143	A	G	0.925	0.128	0.021	sd
Interleukin 18	IL18	rs71478720	Karhunen(PMID:36936266)	cis-pQTL	11	112009605	112138882	C	T	0.756	0.260	0.027	sd
Interleukin 18	IL18	rs113534305	Karhunen(PMID:36936266)	cis-pQTL	11	112096915	112226192	A	G	0.971	0.304	0.065	sd
Interleukin 18	IL18	rs141091241	Karhunen(PMID:36936266)	cis-pQTL	11	112111460	112240737	C	T	0.964	0.392	0.072	sd
Interleukin 18	IL18	rs1534115	Karhunen(PMID:36936266)	cis-pQTL	11	112210999	112340276	A	G	0.675	-0.149	0.025	sd
Interleukin 18	IL18	rs55772679	Karhunen(PMID:36936266)	cis-pQTL	11	112235908	112365185	C	T	0.562	0.134	0.024	sd
Interleukin 18	IL18	rs76785871	Karhunen(PMID:36936266)	cis-pQTL	11	112387246	112516523	G	A	0.892	0.149	0.037	sd
Interleukin 18	IL18	rs10789893	Karhunen(PMID:36936266)	cis-pQTL	11	112391048	112520325	C	T	0.674	-0.113	0.025	sd
Interleukin 6	IL6R	rs2228145	Sarwar(PMID:22421339)	cis-pQTL	1	154426970	154454494	C	A	0.390	0.146	0.020	% increase
Macrophage colony-stimulating factor 1	MCSF	rs6695237	Karhunen(PMID:36936266)	cis-pQTL	1	110415626	109873004	G	A	0.547	0.060	0.012	sd
Macrophage colony-stimulating factor 1	MCSF	rs6699144	Karhunen(PMID:36936266)	cis-pQTL	1	110438926	109896304	C	T	0.688	-0.060	0.013	sd
Macrophage colony-stimulating factor 1	MCSF	rs333968	Karhunen(PMID:36936266)	cis-pQTL	1	110460520	109917898	C	T	0.120	0.141	0.018	sd
Macrophage colony-stimulating factor 1	MCSF	rs1058885	Karhunen(PMID:36936266)	cis-pQTL	1	110466466	109923844	T	C	0.644	-0.126	0.011	sd
Macrophage colony-stimulating factor 1	MCSF	rs72687271	Karhunen(PMID:36936266)	cis-pQTL	1	110491364	109948742	G	A	0.912	0.109	0.025	sd
Macrophage colony-stimulating factor 1	MCSF	rs11579145	Karhunen(PMID:36936266)	cis-pQTL	1	110501597	109958975	G	A	0.481	0.175	0.011	sd
Macrophage colony-stimulating factor 1	MCSF	rs17668350	Karhunen(PMID:36936266)	cis-pQTL	1	110543223	110000601	T	C	0.929	-0.146	0.022	sd
Macrophage colony-stimulating factor 1	MCSF	rs140223635	Karhunen(PMID:36936266)	cis-pQTL	1	110543879	110001257	G	A	0.916	0.110	0.026	sd
Macrophage colony-stimulating factor 1	MCSF	rs74117325	Karhunen(PMID:36936266)	cis-pQTL	1	110597597	110054975	T	G	0.989	-0.307	0.072	sd
Macrophage colony-stimulating factor 1	MCSF	rs3768471	Karhunen(PMID:36936266)	cis-pQTL	1	110612436	110069814	G	A	0.923	-0.170	0.023	sd
Macrophage colony-stimulating factor 1	MCSF	rs116675658	Karhunen(PMID:36936266)	cis-pQTL	1	110613543	110070921	G	T	0.944	0.126	0.027	sd
Macrophage colony-stimulating factor 1	MCSF	rs672992	Karhunen(PMID:36936266)	cis-pQTL	1	110639873	110097251	C	T	0.447	-0.080	0.012	sd
Tumor necrosis factor-alpha	TNFa	rs115497794	Karhunen(PMID:36936266)	cis-pQTL	6	31328974	31361197	G	A	0.904	-0.126	0.029	sd
Tumor necrosis factor-alpha	TNFa	rs2429642	Karhunen(PMID:36936266)	cis-pQTL	6	31486962	31519185	G	A	0.833	0.077	0.019	sd

SNP	CHIP Overall												
SNP	ncases	ncontrols	proxy	r2_1000eur	grch37_proxy	grch38_proxy	EA_proxy	OA_proxy	EA	OA	EAF	BETA	SE
rs149327578	25657	342869							G	A	0.831	-0.007	0.013
rs4656241	25657	342869							T	C	0.800	-0.018	0.012
rs77013776	25657	342869							A	C	0.961	0.030	0.024
rs3093059	25634	342667							A	G	0.943	-0.011	0.020
rs4546916	25657	342869							G	T	0.672	-0.005	0.010
rs6444430	25657	342869							A	G	0.794	0.017	0.012
rs2885370	25657	342869							T	G	0.744	0.022	0.011
rs6801017	25657	342869							G	A	0.210	0.000	0.012
rs67249092	25657	342869							G	A	0.154	-0.011	0.013
rs3935774	25657	342869							T	C	0.013	0.064	0.042
rs113553332	25657	342869							T	C	0.048	0.031	0.022
rs9308676	25657	342869							G	A	0.570	-0.008	0.010
rs7594852	25612	342263							C	T	0.521	-0.010	0.009
rs3783548	25657	342869							G	A	0.091	0.001	0.017
rs1143636	25636	342434							A	G	0.983	0.000	0.036
rs148433212	25657	342869							G	T	0.967	-0.013	0.027
rs4591347	25657	342869							C	T	0.851	-0.004	0.013
rs7584409	25657	342869							G	A	0.307	0.006	0.010
rs115652897	25657	342869							C	T	0.979	-0.031	0.033
rs114189538	25657	342869							G	A	0.935	0.043	0.019
rs115853107	25657	342869							A	C	0.990	-0.058	0.047
rs6743376	25657	342869							C	A	0.350	0.017	0.010
rs139076984	25657	342869							T	G	0.982	0.013	0.036
rs6734238	25581	341893							A	G	0.597	0.006	0.010
rs146220584	25657	342869	rs74500885	1	113839505	113081928	C	T	T	G	0.987	0.025	0.041
rs72827900	25657	342869							C	T	0.951	0.003	0.022
rs6723639	25657	342869							C	T	0.807	-0.018	0.012
rs74667587	25604	342194							G	A	0.822	0.004	0.012
rs72829856	25657	342869							G	A	0.907	-0.002	0.016
rs45592842	25657	342869							T	G	0.916	-0.007	0.017
rs72831808	25470	340289							G	C	0.945	0.010	0.020
rs1491585	25657	342869							A	G	0.557	0.005	0.009
rs1191686	25657	342869							T	C	0.194	-0.003	0.012
rs1867868	25657	342869							T	C	0.505	0.003	0.009
rs719249	25657	342869							A	G	0.895	-0.016	0.015
rs11888059	25657	342869							A	G	0.499	0.000	0.009
rs1030022	25657	342869							T	G	0.701	-0.004	0.010
rs3755295	25657	342869							G	A	0.354	-0.007	0.010
rs3732131	25657	342869							A	G	0.927	-0.006	0.018
rs71478720	25657	342869							C	T	0.744	-0.018	0.011
rs113534305	25618	342092							A	G	0.975	0.010	0.030
rs141091241	25657	342869							C	T	0.965	-0.047	0.026
rs1534115	25657	342869							A	G	0.681	0.014	0.010
rs55772679	25657	342869							C	T	0.547	0.004	0.010
rs76785871	25657	342869							G	A	0.894	-0.002	0.015
rs10789893	25657	342869							C	T	0.700	0.002	0.010
rs2228145	25655	342807							C	A	0.408	-0.011	0.009
rs6695237	25657	342869							G	A	0.553	0.011	0.009
rs6699144	25657	342869							C	T	0.693	-0.017	0.010
rs333968	25657	342869							C	T	0.090	-0.034	0.017
rs1058885	25657	342869							T	C	0.628	-0.002	0.010
rs72687271	25657	342869							G	A	0.924	0.015	0.018
rs11579145	25657	342869							G	A	0.483	-0.006	0.009
rs17668350	25599	342030							T	C	0.921	0.015	0.017
rs140223635	25657	342869							G	A	0.921	0.024	0.018
rs74117325	25657	342869							T	G	0.992	-0.015	0.053
rs3768471	25657	342869							G	A	0.934	0.024	0.019
rs116675658	25657	342869							G	T	0.945	-0.007	0.021
rs672992	25657	342869							C	T	0.476	-0.002	0.010
rs115497794	25657	342869							G	A	0.922	0.016	0.017
rs2429642	25657	342869							G	A	0.837	-0.017	0.013

SNP	DNMT3A CHIP												
SNP	ncases	ncontrols	proxy	r2_1000eur	grch37_proxy	grch38_proxy	EA_proxy	OA_proxy	EA	OA	EAF	BETA	SE
rs149327578	16219	342869							G	A	0.831	-0.009	0.016
rs4656241	16219	342869							T	C	0.800	-0.019	0.015
rs77013776	16219	342869							A	C	0.961	0.038	0.030
rs3093059	16205	342667							A	G	0.943	-0.032	0.025
rs4546916	16219	342869							G	T	0.672	-0.003	0.012
rs6444430	16219	342869							A	G	0.794	0.017	0.015
rs2885370	16219	342869							T	G	0.744	0.024	0.014
rs6801017	16219	342869							G	A	0.210	-0.010	0.014
rs67249092	16219	342869							G	A	0.154	-0.019	0.016
rs3935774	16219	342869							T	C	0.013	0.106	0.050
rs113553332	16219	342869							T	C	0.048	0.053	0.027
rs9308676	16219	342869							G	A	0.570	-0.012	0.012
rs7594852	16192	342263							C	T	0.521	-0.012	0.012
rs3783548	16219	342869							G	A	0.091	0.004	0.021
rs1143636	16205	342434							A	G	0.983	-0.052	0.044
rs148433212	16219	342869							G	T	0.967	-0.012	0.033
rs4591347	16219	342869							C	T	0.851	0.009	0.016
rs7584409	16219	342869							G	A	0.308	0.018	0.013
rs115652897	16219	342869							C	T	0.979	-0.022	0.041
rs114189538	16219	342869							G	A	0.935	0.048	0.024
rs115853107	16219	342869							A	C	0.990	-0.034	0.058
rs6743376	16219	342869							C	A	0.350	0.016	0.012
rs139076984	16219	342869							T	G	0.982	-0.031	0.044
rs6734238	16179	341893							A	G	0.597	-0.006	0.012
rs146220584	16219	342869	rs74500885	1	113839505	113081928	C	T	T	G	0.987	0.001	0.051
rs72827900	16219	342869							C	T	0.951	-0.008	0.027
rs6723639	16219	342869							C	T	0.807	-0.016	0.015
rs74667587	16180	342194							G	A	0.822	0.007	0.015
rs72829856	16219	342869							G	A	0.907	-0.003	0.020
rs45592842	16219	342869							T	G	0.916	-0.029	0.021
rs72831808	16118	340289							G	C	0.945	0.027	0.025
rs1491585	16219	342869							A	G	0.557	0.017	0.012
rs1191686	16219	342869							T	C	0.194	-0.003	0.015
rs1867868	16219	342869							T	C	0.505	0.002	0.012
rs719249	16219	342869							A	G	0.895	-0.032	0.019
rs11888059	16219	342869							A	G	0.499	-0.008	0.012
rs1030022	16219	342869							T	G	0.701	-0.009	0.013
rs3755295	16219	342869							G	A	0.354	-0.012	0.012
rs3732131	16219	342869							A	G	0.927	0.001	0.022
rs71478720	16219	342869							C	T	0.744	-0.031	0.013
rs113534305	16197	342092							A	G	0.975	-0.012	0.037
rs141091241	16219	342869							C	T	0.965	-0.071	0.032
rs1534115	16219	342869							A	G	0.681	0.019	0.012
rs55772679	16219	342869							C	T	0.547	0.010	0.012
rs76785871	16219	342869							G	A	0.893	-0.008	0.019
rs10789893	16219	342869							C	T	0.700	0.000	0.013
rs2228145	16218	342807							C	A	0.408	-0.006	0.012
rs6695237	16219	342869							G	A	0.553	0.001	0.012
rs6699144	16219	342869							C	T	0.694	0.000	0.013
rs333968	16219	342869							C	T	0.090	-0.023	0.021
rs1058885	16219	342869							T	C	0.627	-0.011	0.012
rs72687271	16219	342869							G	A	0.924	0.019	0.023
rs11579145	16219	342869							G	A	0.483	-0.008	0.012
rs17668350	16179	342030							T	C	0.921	0.022	0.021
rs140223635	16219	342869							G	A	0.921	0.036	0.022
rs74117325	16219	342869							T	G	0.992	0.007	0.065
rs3768471	16219	342869							G	A	0.934	0.019	0.024
rs116675658	16219	342869							G	T	0.945	0.004	0.026
rs672992	16219	342869							C	T	0.476	0.003	0.012
rs115497794	16219	342869							G	A	0.922	0.022	0.021
rs2429642	16219	342869							G	A	0.837	-0.015	0.016

SNP	TET2 CHIP												
SNP	ncases	ncontrols	proxy	r2_1000eur	grch37_proxy	grch38_proxy	EA_proxy	OA_proxy	EA	OA	EAF	BETA	SE
rs149327578	3918	342869							G	A	0.831	-0.048	0.031
rs4656241	3918	342869							T	C	0.801	0.028	0.029
rs77013776	3918	342869							A	C	0.961	0.043	0.059
rs3093059	3913	342667							A	G	0.943	0.026	0.050
rs4546916	3918	342869							G	T	0.672	-0.013	0.025
rs6444430	3918	342869							A	G	0.794	0.009	0.030
rs2885370	3918	342869							T	G	0.744	0.002	0.028
rs6801017	3918	342869							G	A	0.210	0.000	0.029
rs67249092	3918	342869							G	A	0.154	-0.002	0.032
rs3935774	3918	342869							T	C	0.013	-0.042	0.104
rs113553332	3918	342869							T	C	0.048	0.004	0.054
rs9308676	3918	342869							G	A	0.570	-0.019	0.023
rs7594852	3909	342263							C	T	0.521	-0.005	0.023
rs3783548	3918	342869							G	A	0.091	-0.050	0.041
rs1143636	3915	342434							A	G	0.983	0.107	0.088
rs148433212	3918	342869							G	T	0.967	-0.041	0.065
rs4591347	3918	342869							C	T	0.851	-0.040	0.032
rs7584409	3918	342869							G	A	0.307	-0.027	0.025
rs115652897	3918	342869							C	T	0.979	0.026	0.081
rs114189538	3918	342869							G	A	0.935	0.052	0.047
rs115853107	3918	342869							A	C	0.990	-0.206	0.116
rs6743376	3918	342869							C	A	0.350	0.000	0.024
rs139076984	3918	342869							T	G	0.982	-0.004	0.087
rs6734238	3901	341893							A	G	0.597	0.011	0.023
rs146220584	3918	342869	rs74500885	1	113839505	113081928	C	T	T	G	0.987	0.041	0.101
rs72827900	3918	342869							C	T	0.951	0.088	0.054
rs6723639	3918	342869							C	T	0.807	-0.051	0.029
rs74667587	3913	342194							G	A	0.822	0.024	0.030
rs72829856	3918	342869							G	A	0.907	0.013	0.040
rs45592842	3918	342869							T	G	0.916	-0.021	0.041
rs72831808	3886	340289							G	C	0.945	-0.008	0.050
rs1491585	3918	342869							A	G	0.557	-0.003	0.023
rs1191686	3918	342869							T	C	0.194	0.014	0.029
rs1867868	3918	342869							T	C	0.505	0.016	0.023
rs719249	3918	342869							A	G	0.895	-0.008	0.037
rs11888059	3918	342869							A	G	0.499	0.010	0.023
rs1030022	3918	342869							T	G	0.701	0.002	0.025
rs3755295	3918	342869							G	A	0.354	-0.007	0.024
rs3732131	3918	342869							A	G	0.927	-0.011	0.044
rs71478720	3918	342869							C	T	0.744	-0.022	0.026
rs113534305	3909	342092							A	G	0.975	-0.063	0.075
rs141091241	3918	342869							C	T	0.965	-0.038	0.065
rs1534115	3918	342869							A	G	0.681	0.021	0.025
rs55772679	3918	342869							C	T	0.547	-0.029	0.023
rs76785871	3918	342869							G	A	0.893	-0.024	0.038
rs10789893	3918	342869							C	T	0.700	0.039	0.025
rs2228145	3917	342807							C	A	0.408	-0.024	0.023
rs6695237	3918	342869							G	A	0.553	0.016	0.023
rs6699144	3918	342869							C	T	0.694	-0.036	0.025
rs333968	3918	342869							C	T	0.090	-0.008	0.041
rs1058885	3918	342869							T	C	0.628	0.016	0.024
rs72687271	3918	342869							G	A	0.923	-0.045	0.045
rs11579145	3918	342869							G	A	0.483	-0.004	0.023
rs17668350	3909	342030							T	C	0.921	0.015	0.043
rs140223635	3918	342869							G	A	0.921	0.019	0.044
rs74117325	3918	342869							T	G	0.992	-0.112	0.129
rs3768471	3918	342869							G	A	0.934	0.067	0.048
rs116675658	3918	342869							G	T	0.945	-0.046	0.052
rs672992	3918	342869							C	T	0.476	0.010	0.023
rs115497794	3918	342869							G	A	0.922	0.028	0.043
rs2429642	3918	342869							G	A	0.837	-0.012	0.031

Abbreviations: SNP, Single-nucleotide polymorphism; CHR, Chromosome; Pos, Position; EA, Effect Allele; OA, Other Allele; EAF, effect allele frequency; SE, standard error; IV, Instrumental variant; ncases, number of cases; ncontrols, number of controls; grch, gene reference consortium and CHIP, clonal hematopoiesis of indeterminate potential

Supplemental table 5: Characteristics of instrumental variables for cardiovascular disease and their associations with CHIP

Instrumental Variable							CVD				CHIP Overall				DNMT3A CHIP				TET2 CHIP			
Exposure	Sample Size	SNP	CHR	Pos	EA	OA	EAF	BETA	SE	P	EAF	BETA	SE	P	EAF	BETA	SE	P	EAF	BETA	SE	P
Coronary artery disease || id:ebi-a-GCST003116	42457	rs10080815	6	160687412	G	T	0.028	0.247	0.031	1.33E-15	0.020	0.006	0.037	0.875783	0.020	0.035	0.042	0.401679	0.020	0.010	0.084	0.905725
Coronary artery disease || id:ebi-a-GCST003116	42457	rs10840293	11	9751196	A	G	0.550	0.055	0.010	1.28E-08	0.557	-0.006	0.010	0.550127	0.556	-0.017	0.012	0.144912	0.557	0.012	0.023	0.610856
Coronary artery disease || id:ebi-a-GCST003116	42457	rs11065979	12	112059557	T	C	0.365	0.069	0.011	1.93E-10	0.437	0.013	0.010	0.20863	0.437	0.001	0.012	0.897915	0.437	0.030	0.023	0.200951
Coronary artery disease || id:ebi-a-GCST003116	42457	rs11191416	10	104604916	G	T	0.127	-0.079	0.014	4.65E-09	0.084	0.024	0.019	0.20221	0.084	0.041	0.020	0.0475317	0.084	-0.018	0.041	0.66853
Coronary artery disease || id:ebi-a-GCST003116	42457	rs11556924	7	129663496	T	C	0.313	-0.073	0.011	5.34E-11	0.389	0.018	0.011	0.0923961	0.389	0.026	0.012	0.0255983	0.389	0.042	0.024	0.072667
Coronary artery disease || id:ebi-a-GCST003116	42457	rs115654617	2	203893999	A	C	0.107	0.138	0.016	3.12E-18	0.127	-0.006	0.015	0.679932	0.127	-0.027	0.017	0.118622	0.127	-0.034	0.034	0.318907
Coronary artery disease || id:ebi-a-GCST003116	42457	rs11838776	13	111040681	A	G	0.263	0.069	0.011	1.83E-10	0.278	-0.003	0.012	0.817148	0.278	-0.006	0.013	0.644703	0.278	0.009	0.026	0.734169
Coronary artery disease || id:ebi-a-GCST003116	42457	rs1199338	3	138087467	C	A	0.162	0.074	0.012	3.90E-09	0.161	0.014	0.014	0.307489	0.161	0.014	0.016	0.382102	0.161	0.031	0.031	0.321529
Coronary artery disease || id:ebi-a-GCST003116	42457	rs12202017	6	134173151	G	A	0.300	-0.067	0.010	1.98E-11	0.289	-0.002	0.011	0.827264	0.289	-0.008	0.013	0.547514	0.289	-0.050	0.026	0.0517254
Coronary artery disease || id:ebi-a-GCST003116	42457	rs1412444	10	91002927	T	C	0.369	0.067	0.010	5.15E-12	0.340	-0.011	0.011	0.317465	0.339	-0.030	0.012	0.0127708	0.340	-0.017	0.024	0.472045
Coronary artery disease || id:ebi-a-GCST003116	42457	rs16986953	2	19942473	A	G	0.105	0.085	0.015	1.45E-08	0.068	0.006	0.021	0.774689	0.068	0.000	0.023	0.988717	0.068	0.040	0.046	0.389987
Coronary artery disease || id:ebi-a-GCST003116	42457	rs17087335	4	57838583	T	G	0.215	0.061	0.011	4.59E-08	0.187	-0.008	0.013	0.524096	0.187	0.000	0.015	0.987024	0.187	0.000	0.030	0.98822
Coronary artery disease || id:ebi-a-GCST003116	42457	rs17678683	2	145286559	G	T	0.088	0.099	0.017	3.00E-09	0.086	0.004	0.018	0.849672	0.086	-0.023	0.021	0.263643	0.086	-0.037	0.041	0.364133
Coronary artery disease || id:ebi-a-GCST003116	42457	rs1870634	10	44480811	G	T	0.637	0.076	0.010	5.55E-15	0.665	-0.007	0.011	0.548147	0.665	-0.006	0.012	0.648866	0.665	-0.023	0.024	0.337416
Coronary artery disease || id:ebi-a-GCST003116	42457	rs2107595	7	19049388	A	G	0.200	0.073	0.011	8.05E-11	0.153	-0.004	0.014	0.795423	0.153	-0.005	0.016	0.758084	0.153	-0.001	0.032	0.969183
Coronary artery disease || id:ebi-a-GCST003116	42457	rs2128739	11	103673277	C	A	0.676	-0.066	0.010	7.05E-11	0.716	0.008	0.011	0.487083	0.716	0.000	0.013	0.97416	0.716	-0.009	0.026	0.735414
Coronary artery disease || id:ebi-a-GCST003116	42457	rs2487928	10	30323892	A	G	0.418	0.063	0.010	4.41E-11	0.444	0.014	0.010	0.171899	0.444	0.024	0.012	0.0372184	0.444	0.010	0.023	0.677782
Coronary artery disease || id:ebi-a-GCST003116	42457	rs2681472	12	90008959	G	A	0.201	0.074	0.011	6.17E-11	0.172	-0.012	0.014	0.36039	0.172	-0.014	0.015	0.369685	0.172	0.031	0.031	0.314914
Coronary artery disease || id:ebi-a-GCST003116	42457	rs28451064	21	35593827	A	G	0.121	0.128	0.016	1.33E-15	0.131	-0.019	0.016	0.225103	0.131	0.004	0.017	0.812537	0.131	-0.040	0.035	0.247547
Coronary artery disease || id:ebi-a-GCST003116	42457	rs2891168	9	22098619	G	A	0.489	0.193	0.009	2.29E-98	0.486	-0.011	0.010	0.269688	0.486	-0.009	0.012	0.415142	0.486	0.053	0.023	0.021303
Coronary artery disease || id:ebi-a-GCST003116	42457	rs3918226	7	150690176	T	C	0.065	0.133	0.022	1.69E-09	0.082	0.010	0.019	0.585248	0.082	0.013	0.021	0.526438	0.082	-0.016	0.042	0.710945
Coronary artery disease || id:ebi-a-GCST003116	42457	rs4420638	19	45422946	G	A	0.166	0.092	0.014	7.07E-11	0.189	-0.007	0.013	0.615252	0.189	-0.008	0.015	0.568342	0.189	0.001	0.029	0.965742
Coronary artery disease || id:ebi-a-GCST003116	42457	rs4468572	15	79124475	C	T	0.586	0.077	0.010	4.44E-16	0.570	-0.016	0.011	0.118319	0.570	-0.015	0.012	0.202644	0.570	-0.002	0.023	0.929755
Coronary artery disease || id:ebi-a-GCST003116	42457	rs4593108	4	148281001	G	C	0.205	-0.071	0.012	8.82E-10	0.172	0.005	0.014	0.725885	0.172	0.011	0.015	0.467788	0.172	-0.022	0.031	0.475519
Coronary artery disease || id:ebi-a-GCST003116	42457	rs515135	2	21286057	C	T	0.792	0.067	0.012	3.09E-08	0.819	-0.007	0.013	0.575735	0.819	0.016	0.015	0.275577	0.819	-0.035	0.030	0.246316
Coronary artery disease || id:ebi-a-GCST003116	42457	rs55730499	6	161005610	T	C	0.056	0.317	0.024	5.39E-39	0.080	-0.012	0.019	0.519896	0.080	-0.023	0.021	0.287672	0.080	-0.039	0.042	0.359387
Coronary artery disease || id:ebi-a-GCST003116	42457	rs56062135	15	67455630	T	C	0.206	-0.070	0.012	4.52E-09	0.235	0.003	0.012	0.777564	0.235	0.005	0.014	0.727806	0.235	0.001	0.027	0.969647
Coronary artery disease || id:ebi-a-GCST003116	42457	rs56225615	15	79139370	T	C	0.156	0.077	0.013	3.57E-09	0.148	-0.017	0.015	0.252962	0.148	-0.013	0.016	0.436363	0.148	-0.021	0.033	0.512799
Coronary artery disease || id:ebi-a-GCST003116	42457	rs56289821	19	11188247	A	G	0.100	-0.134	0.017	4.44E-15	0.118	-0.016	0.016	0.302481	0.118	-0.022	0.018	0.208858	0.118	-0.022	0.036	0.546923
Coronary artery disease || id:ebi-a-GCST003116	42457	rs56336142	6	39134099	C	T	0.193	-0.067	0.012	1.85E-08	0.211	-0.004	0.013	0.781824	0.211	-0.005	0.014	0.70591	0.211	0.007	0.028	0.802365
Coronary artery disease || id:ebi-a-GCST003116	42457	rs663129	18	57838401	A	G	0.257	0.058	0.011	3.20E-08	0.232	-0.010	0.012	0.405705	0.233	-0.007	0.014	0.587538	0.233	-0.005	0.027	0.849433
Coronary artery disease || id:ebi-a-GCST003116	42457	rs6689306	1	154395946	G	A	0.552	-0.056	0.009	2.60E-09	0.577	-0.007	0.011	0.493223	0.577	-0.003	0.012	0.816809	0.577	-0.050	0.024	0.0360342
Coronary artery disease || id:ebi-a-GCST003116	42457	rs67180937	1	222823743	G	T	0.663	0.079	0.011	1.01E-12	0.713	-0.005	0.011	0.683155	0.713	-0.002	0.013	0.890896	0.713	-0.021	0.026	0.40367
Coronary artery disease || id:ebi-a-GCST003116	42457	rs7212798	17	59013488	C	T	0.147	0.080	0.014	1.88E-08	0.148	-0.018	0.015	0.229298	0.147	-0.042	0.016	0.00997497	0.148	0.031	0.033	0.347459
Coronary artery disease || id:ebi-a-GCST003116	42457	rs7528419	1	109817192	G	A	0.214	-0.115	0.011	1.97E-23	0.222	-0.004	0.012	0.775364	0.222	-0.018	0.014	0.206967	0.222	0.017	0.028	0.529929
Coronary artery disease || id:ebi-a-GCST003116	42457	rs8042271	15	89574218	A	G	0.098	-0.097	0.018	3.68E-08	0.036	0.019	0.028	0.504143	0.036	0.025	0.031	0.418998	0.036	0.095	0.062	0.128049
Coronary artery disease || id:ebi-a-GCST003116	42457	rs9349379	6	12903957	G	A	0.432	0.132	0.010	1.81E-42	0.406	0.014	0.010	0.178861	0.406	0.004	0.012	0.759284	0.406	0.016	0.023	0.492663
Coronary artery disease || id:ebi-a-GCST003116	42457	rs9970807	1	56965664	T	C	0.085	-0.126	0.017	5.00E-14	0.092	-0.029	0.018	0.107799	0.092	-0.044	0.020	0.0303651	0.092	-0.069	0.040	0.083165
Atrial fibrillation || id:ebi-a-GCST006414	1030836	rs10141892	14	35184323	C	T	0.583	-0.045	0.007	2.95E-11	0.586	0.004	0.010	0.725585	0.586	-0.002	0.012	0.835243	0.586	0.009	0.023	0.715025
Atrial fibrillation || id:ebi-a-GCST006414	1030836	rs10213171	4	148937537	G	C	0.061	0.091	0.013	1.32E-11	0.056	0.018	0.023	0.419477	0.056	0.027	0.025	0.281062	0.056	0.027	0.050	0.597242
Atrial fibrillation || id:ebi-a-GCST006414	1030836	rs10458662	10	77936670	G	T	0.172	0.054	0.009	6.93E-10	0.166	-0.006	0.014	0.650943	0.166	-0.008	0.016	0.60152	0.166	-0.035	0.031	0.260987
Atrial fibrillation || id:ebi-a-GCST006414	1030836	rs10520002	5	127819132	A	G	0.099	0.063	0.011	2.85E-08	0.100	0.006	0.017	0.749286	0.100	0.007	0.019	0.720451	0.100	0.009	0.038	0.817072
Atrial fibrillation || id:ebi-a-GCST006414	1030836	rs10520260	4	174447349	G	A	0.321	-0.046	0.007	3.36E-10	0.319	0.010	0.011	0.354993	0.319	0.008	0.013	0.529688	0.319	0.002	0.025	0.946952
Atrial fibrillation || id:ebi-a-GCST006414	1030836	rs10753933	1	203026214	G	T	0.552	-0.061	0.007	9.84E-20	0.548	-0.001	0.010	0.944099	0.548	0.000	0.012	0.974409	0.548	0.022	0.023	0.348042
Atrial fibrillation || id:ebi-a-GCST006414	1030836	rs10773657	12	123327900	A	C	0.862	-0.058	0.010	2.54E-08	0.894	-0.006	0.017	0.736518	0.894	-0.010	0.019	0.607675	0.894	0.029	0.037	0.434856
Atrial fibrillation || id:ebi-a-GCST006414	1030836	rs10804493	3	111554426	A	G	0.651	0.056	0.007	1.63E-15	0.668	0.002	0.011	0.869049	0.668	0.005	0.012	0.679996	0.667	-0.030	0.024	0.214584
Atrial fibrillation || id:ebi-a-GCST006414	1030836	rs10821415	9	97713459	A	C	0.413	0.082	0.007	2.92E-34	0.419	0.001	0.010	0.943282	0.419	0.010	0.012	0.390141	0.419	-0.047	0.023	0.0449053
Atrial fibrillation || id:ebi-a-GCST006414	1030836	rs10842383	12	24771967	T	C	0.148	-0.099	0.010	2.88E-25	0.150	-0.008	0.014	0.571088	0.150	-0.010	0.016	0.539768	0.150	-0.015	0.032	0.629133
Atrial fibrillation || id:ebi-a-GCST006414	1030836	rs11191116	10	103555611	T	C	0.348	-0.041	0.007	4.42E-09	0.350	-0.007	0.011	0.50961	0.350	-0.006	0.012	0.623798	0.350	0.017	0.024	0.48749
Atrial fibrillation || id:ebi-a-GCST006414	1030836	rs11264280	1	154862952	T	C	0.333	0.135	0.007	3.07E-79	0.324	0.010	0.011	0.351991	0.324	0.018	0.012	0.154539	0.323	0.005	0.025	0.842122
Atrial fibrillation || id:ebi-a-GCST006414	1030836	rs11598047	10	105342672	G	A	0.162	0.154	0.009	8.95E-66	0.150	0.006	0.014	0.671678	0.150	0.013	0.016	0.410901	0.150	-0.043	0.032	0.186293
Atrial fibrillation || id:ebi-a-GCST006414	1030836	rs11773845	7	116191301	A	C	0.586	0.105	0.007	2.39E-55	0.591	-0.007	0.010	0.480714	0.591	-0.012	0.012	0.3056	0.591	-0.021	0.023	0.372874
Atrial fibrillation || id:ebi-a-GCST006414	1030836	rs117984853	6	149399100	T	G	0.101	0.123	0.012	1.34E-24	0.090	-0.001	0.018	0.9402	0.090	-0.003	0.020	0.867463	0.090	0.060	0.040	0.133386
Atrial fibrillation || id:ebi-a-GCST006414	1030836	rs12245149	10	65321147	A	C	0.474	-0.047	0.007	1.66E-12	0.487	-0.016	0.010	0.126943	0.487	-0.025	0.012	0.0301874	0.487	-0.022	0.023	0.350256
Atrial fibrillation || id:ebi-a-GCST006414	1030836	rs12426679	12	76237987	T	C	0.528	-0.039	0.007	4.95E-09	0.509	0.004	0.010	0.691342	0.509	-0.003	0.012	0.781161	0.509	0.021	0.023	0.355699
Atrial fibrillation || id:ebi-a-GCST006414	1030836	rs1278493	3	135814009	A	G	0.565	-0.039	0.007	8.77E-09	0.566	0.010	0.010	0.351727	0.566	0.006	0.012	0.631724	0.566	0.056	0.023	0.0162249
Atrial fibrillation || id:ebi-a-GCST006414	1030836	rs133885	22	26159289	A	G	0.438	0.041	0.007	2.22E-09	0.446	-0.016	0.010	0.123211	0.446	-0.014	0.012	0.228326	0.446	0.018	0.023	0.424619
Atrial fibrillation || id:ebi-a-GCST006414	1030836	rs140185678	16	2003016	A	G	0.035	0.166	0.022	2.43E-14	0.035	0.051	0.028	0.0686367	0.035	0.022	0.031	0.474426	0.035	0.004	0.063	0.944843
Atrial fibrillation || id:ebi-a-GCST006414	1030836	rs1458038	4	81164723	T	C	0.309	0.043	0.007	1.74E-09	0.289	-0.001	0.011	0.962854	0.289	-0.011	0.013	0.389013	0.289	0.025	0.025	0.327293
Atrial fibrillation || id:ebi-a-GCST006414	1030836	rs146518726	1	51535039	A	G	0.033	0.161	0.021	8.27E-15	0.021	0.028	0.036	0.43755	0.021	0.041	0.040	0.313681	0.020	-0.027	0.081	0.733898
Atrial fibrillation || id:ebi-a-GCST006414	1030836	rs1563304	17	44874453	T	C	0.178	0.064	0.009	2.56E-12	0.178	0.006	0.014	0.673099	0.178	0.001	0.015	0.938312	0.178	0.006	0.030	0.832855
Atrial fibrillation || id:ebi-a-GCST006414	1030836	rs17171711	5	137364795	T	C	0.178	0.109	0.009	1.95E-35	0.178	-0.008	0.013	0.543327	0.178	0.000	0.015	0.974786	0.178	-0.027	0.030	0.373806
Atrial fibrillation || id:ebi-a-GCST006414	1030836	rs17380837	12	26345526	T	C	0.307	-0.050	0.007	4.80E-12	0.296	-0.016	0.011	0.14474	0.296	-0.013	0.013	0.287248	0.297	0.007	0.025	0.79048
Atrial fibrillation || id:ebi-a-GCST006414	1030836	rs1838747	5	114426668	G	A	0.495	0.039	0.007	4.13E-09	0.491	-0.007	0.010	0.526089	0.491	-0.001	0.012	0.955297	0.491	-0.031	0.023	0.172838
Atrial fibrillation || id:ebi-a-GCST006414	1030836	rs1906615	4	111701798	T	G	0.199	0.366	0.008	1.00E-200	0.196	-0.009	0.013	0.506337	0.196	-0.007	0.015	0.623361	0.196	0.012	0.029	0.684412
Atrial fibrillation || id:ebi-a-GCST006414	1030836	rs2031522	6	87821501	G	A	0.376	-0.044	0.007	1.47E-10	0.386	-0.013	0.011	0.224057	0.387	-0.013	0.012	0.257607	0.387	-0.020	0.024	0.394653
Atrial fibrillation || id:ebi-a-GCST006414	1030836	rs2274115	9	139094773	G	A	0.700	0.049	0.008	1.69E-10	0.714	-0.001	0.011	0.945055	0.714	0.001	0.013	0.935922	0.714	-0.043	0.025	0.089204
Atrial fibrillation || id:ebi-a-GCST006414	1030836	rs2288327	2	179411665	G	A	0.156	0.092	0.009	7.26E-25	0.158	0.005	0.014	0.707841	0.158	0.000	0.016	0.996361	0.158	0.055	0.032	0.0817124
Atrial fibrillation || id:ebi-a-GCST006414	1030836	rs2359171	16	73053022	A	T	0.176	0.175	0.009	4.65E-91	0.166	0.003	0.014	0.831777	0.166	0.009	0.015	0.577299	0.166	-0.016	0.031	0.602212
Atrial fibrillation || id:ebi-a-GCST006414	1030836	rs2540949	2	65284231	T	A	0.385	-0.066	0.007	2.95E-22	0.375	-0.011	0.011	0.310128	0.375	0.008	0.012	0.514327	0.375	-0.023	0.024	0.326513
Atrial fibrillation || id:ebi-a-GCST006414	1030836	rs2738413	14	64679960	G	A	0.505	-0.078	0.007	2.55E-31	0.509	-0.007	0.010	0.525541	0.509	-0.003	0.012	0.79491	0.509	-0.011	0.023	0.620812
Atrial fibrillation || id:ebi-a-GCST006414	1030836	rs2759301	15	80994288	A	G	0.454	0.039	0.007	5.04E-09	0.445	-0.010	0.010	0.314351	0.445	-0.015	0.012	0.187093	0.446	0.001	0.023	0.95336
Atrial fibrillation || id:ebi-a-GCST006414	1030836	rs2834618	21	36119111	G	T	0.106	-0.094	0.011	3.41E-17	0.099	-0.023	0.017	0.187878	0.099	0.002	0.019	0.912469	0.099	-0.064	0.039	0.0956527
Atrial fibrillation || id:ebi-a-GCST006414	1030836	rs28387148	2	127433465	T	C	0.105	0.074	0.011	6.25E-11	0.099	-0.023	0.017	0.182354	0.099	-0.015	0.019	0.431785	0.099	-0.051	0.038	0.185917
Atrial fibrillation || id:ebi-a-GCST006414	1030836	rs284277	1	10790797	A	C	0.617	-0.042	0.007	1.25E-09	0.635	-0.014	0.011	0.189874	0.635	-0.015	0.012	0.232495	0.635	-0.025	0.024	0.309825
Atrial fibrillation || id:ebi-a-GCST006414	1030836	rs2860482	12	57105938	C	A	0.726	-0.054	0.008	1.21E-12	0.717	-0.009	0.012	0.43424	0.717	-0.019	0.013	0.143689	0.717	-0.010	0.026	0.693363
Atrial fibrillation || id:ebi-a-GCST006414	1030836	rs28631169	14	23888183	T	C	0.198	0.052	0.008	5.35E-10	0.204	-0.014	0.013	0.264553	0.204	-0.008	0.014	0.563452	0.204	0.000	0.029	0.992309
Atrial fibrillation || id:ebi-a-GCST006414	1030836	rs2885697	1	41544279	T	G	0.648	-0.044	0.007	2.88E-10	0.663	-0.001	0.011	0.916928	0.663	-0.004	0.012	0.753122	0.663	0.005	0.024	0.851178
Atrial fibrillation || id:ebi-a-GCST006414	1030836	rs3176326	6	36647289	A	G	0.198	-0.063	0.009	1.42E-13	0.198	0.003	0.013	0.809288	0.198	0.004	0.014	0.795033	0.198	-0.011	0.029	0.714573
Atrial fibrillation || id:ebi-a-GCST006414	1030836	rs337705	5	113737062	G	T	0.375	0.056	0.007	1.63E-16	0.389	0.018	0.011	0.0876569	0.389	0.000	0.012	0.969427	0.389	0.032	0.024	0.178506
Atrial fibrillation || id:ebi-a-GCST006414	1030836	rs34080181	3	66454191	A	G	0.379	-0.045	0.007	1.28E-10	0.390	-0.007	0.011	0.536358	0.390	-0.007	0.012	0.535446	0.390	-0.055	0.024	0.0207024
Atrial fibrillation || id:ebi-a-GCST006414	1030836	rs34936990	10	105523416	A	G	0.121	0.129	0.010	2.95E-37	0.116	0.053	0.016	9.16E-04	0.116	0.054	0.018	0.00263831	0.116	0.068	0.036	0.0580885
Atrial fibrillation || id:ebi-a-GCST006414	1030836	rs34969716	6	18210109	A	G	0.305	0.070	0.008	1.60E-19	0.319	-0.002	0.011	0.826895	0.319	-0.010	0.013	0.453888	0.319	0.041	0.025	0.103235
Atrial fibrillation || id:ebi-a-GCST006414	1030836	rs35544454	2	213266003	T	A	0.192	-0.059	0.009	1.10E-11	0.181	0.001	0.013	0.926251	0.181	-0.001	0.015	0.927138	0.181	-0.014	0.030	0.634304
Atrial fibrillation || id:ebi-a-GCST006414	1030836	rs35569628	13	113872712	C	T	0.223	-0.045	0.008	1.38E-08	0.225	-0.011	0.012	0.36828	0.225	-0.006	0.014	0.661883	0.225	-0.018	0.028	0.506607
Atrial fibrillation || id:ebi-a-GCST006414	1030836	rs35963991	8	11495702	T	G	0.149	0.053	0.010	2.80E-08	0.154	-0.016	0.014	0.255959	0.154	-0.023	0.016	0.156853	0.154	-0.007	0.032	0.827829
Atrial fibrillation || id:ebi-a-GCST006414	1030836	rs3943207	8	21845619	T	G	0.117	-0.064	0.010	6.92E-10	0.113	0.026	0.016	0.116507	0.113	-0.005	0.018	0.78725	0.113	0.069	0.036	0.0573207
Atrial fibrillation || id:ebi-a-GCST006414	1030836	rs4073778	1	116297758	A	C	0.564	0.049	0.007	4.96E-13	0.568	-0.007	0.010	0.531131	0.568	-0.001	0.012	0.936815	0.568	-0.003	0.023	0.890747
Atrial fibrillation || id:ebi-a-GCST006414	1030836	rs4252627	17	37868715	T	C	0.668	-0.042	0.007	5.63E-09	0.676	-0.003	0.011	0.816255	0.676	-0.004	0.012	0.750033	0.676	-0.009	0.025	0.701987
Atrial fibrillation || id:ebi-a-GCST006414	1030836	rs4587869	14	32992334	C	G	0.285	0.072	0.008	1.19E-20	0.268	0.007	0.012	0.543009	0.269	0.026	0.013	0.0531921	0.268	-0.030	0.027	0.265132
Atrial fibrillation || id:ebi-a-GCST006414	1030836	rs4642101	3	12842223	G	T	0.640	0.071	0.007	2.95E-24	0.647	0.014	0.011	0.207617	0.647	0.014	0.012	0.234901	0.647	0.019	0.024	0.420193
Atrial fibrillation || id:ebi-a-GCST006414	1030836	rs464901	22	18597502	C	T	0.335	-0.051	0.007	1.53E-12	0.338	-0.032	0.011	0.00330241	0.338	-0.031	0.012	0.0118346	0.338	-0.057	0.025	0.0195687
Atrial fibrillation || id:ebi-a-GCST006414	1030836	rs4757877	11	20010291	G	A	0.755	-0.072	0.008	2.93E-20	0.767	0.010	0.012	0.411014	0.767	0.008	0.014	0.554598	0.766	0.002	0.028	0.930034
Atrial fibrillation || id:ebi-a-GCST006414	1030836	rs4935786	11	121661507	A	T	0.733	-0.046	0.008	4.85E-09	0.722	0.015	0.012	0.187169	0.722	0.030	0.013	0.0216877	0.722	-0.022	0.026	0.391283
Atrial fibrillation || id:ebi-a-GCST006414	1030836	rs4946333	6	118565665	G	A	0.490	0.064	0.007	5.47E-22	0.498	0.006	0.010	0.553576	0.498	0.005	0.012	0.642943	0.498	-0.018	0.023	0.443934
Atrial fibrillation || id:ebi-a-GCST006414	1030836	rs4965430	15	99268850	G	C	0.614	-0.044	0.007	1.26E-10	0.622	0.008	0.011	0.445166	0.622	-0.005	0.012	0.655904	0.622	0.040	0.024	0.095712
Atrial fibrillation || id:ebi-a-GCST006414	1030836	rs55734480	7	14372009	A	G	0.249	0.055	0.008	2.20E-12	0.264	0.007	0.012	0.523068	0.264	0.009	0.013	0.484042	0.264	0.009	0.026	0.734867
Atrial fibrillation || id:ebi-a-GCST006414	1030836	rs55985730	7	128417044	G	T	0.060	0.087	0.015	5.24E-09	0.056	0.049	0.022	0.0295496	0.056	0.012	0.025	0.629952	0.056	0.065	0.050	0.193281
Atrial fibrillation || id:ebi-a-GCST006414	1030836	rs56201652	7	92278116	A	G	0.267	-0.053	0.008	1.74E-12	0.269	-0.036	0.012	0.00204527	0.269	-0.047	0.013	3.83E-04	0.269	0.023	0.026	0.384656
Atrial fibrillation || id:ebi-a-GCST006414	1030836	rs56326533	2	201168758	C	T	0.392	0.069	0.007	6.28E-24	0.390	0.001	0.011	0.940662	0.390	0.006	0.012	0.60913	0.390	0.053	0.023	0.0236424
Atrial fibrillation || id:ebi-a-GCST006414	1030836	rs577676	1	170587340	T	C	0.438	-0.092	0.007	1.62E-43	0.437	-0.006	0.010	0.569268	0.437	-0.007	0.012	0.563952	0.437	-0.042	0.023	0.0735533
Atrial fibrillation || id:ebi-a-GCST006414	1030836	rs60902112	3	194800853	T	C	0.226	0.045	0.008	1.72E-08	0.220	-0.017	0.012	0.173398	0.220	-0.018	0.014	0.185241	0.220	-0.011	0.028	0.682534
Atrial fibrillation || id:ebi-a-GCST006414	1030836	rs62254082	3	69417585	C	T	0.387	0.040	0.007	6.34E-09	0.374	0.014	0.011	0.186341	0.374	0.011	0.012	0.37762	0.374	0.001	0.024	0.964103
Atrial fibrillation || id:ebi-a-GCST006414	1030836	rs62377206	5	168383543	A	G	0.055	0.085	0.015	8.21E-09	0.050	0.021	0.024	0.369737	0.050	0.040	0.026	0.129944	0.050	0.051	0.053	0.331844
Atrial fibrillation || id:ebi-a-GCST006414	1030836	rs62521286	8	124551975	G	A	0.066	0.120	0.014	4.50E-19	0.066	0.014	0.021	0.48926	0.066	0.022	0.023	0.351533	0.066	0.037	0.046	0.425659
Atrial fibrillation || id:ebi-a-GCST006414	1030836	rs6462079	7	28415827	A	G	0.721	0.047	0.008	8.79E-10	0.731	-0.018	0.012	0.124969	0.732	-0.003	0.013	0.835211	0.732	-0.018	0.026	0.485963
Atrial fibrillation || id:ebi-a-GCST006414	1030836	rs6546620	2	26159940	C	T	0.800	0.060	0.009	3.19E-12	0.818	-0.011	0.014	0.429685	0.818	-0.018	0.015	0.237484	0.818	-0.028	0.030	0.357364
Atrial fibrillation || id:ebi-a-GCST006414	1030836	rs6560886	12	133150210	C	T	0.788	0.051	0.009	1.49E-08	0.806	0.021	0.013	0.110537	0.806	0.012	0.015	0.425344	0.806	0.091	0.030	0.00264148
Atrial fibrillation || id:ebi-a-GCST006414	1030836	rs6580277	5	142818123	G	A	0.237	0.067	0.008	1.64E-17	0.241	0.001	0.012	0.957661	0.241	-0.003	0.014	0.824389	0.241	0.005	0.027	0.85701
Atrial fibrillation || id:ebi-a-GCST006414	1030836	rs6596717	5	106427609	A	C	0.605	-0.040	0.007	3.00E-09	0.614	0.023	0.011	0.0306945	0.614	0.022	0.012	0.0629787	0.614	0.042	0.024	0.0780333
Atrial fibrillation || id:ebi-a-GCST006414	1030836	rs6665642	1	154802139	T	C	0.118	-0.062	0.011	3.06E-08	0.118	0.002	0.017	0.886285	0.118	0.007	0.019	0.687707	0.118	0.032	0.037	0.382546
Atrial fibrillation || id:ebi-a-GCST006414	1030836	rs6689306	1	154395946	G	A	0.587	-0.046	0.007	1.36E-11	0.577	-0.007	0.011	0.493223	0.577	-0.003	0.012	0.816809	0.577	-0.050	0.024	0.0360342
Atrial fibrillation || id:ebi-a-GCST006414	1030836	rs6747542	2	70106832	C	T	0.464	-0.055	0.007	1.10E-16	0.457	0.016	0.010	0.135046	0.457	0.022	0.012	0.0628151	0.457	0.027	0.023	0.242199
Atrial fibrillation || id:ebi-a-GCST006414	1030836	rs6771054	3	89489529	C	T	0.404	-0.046	0.007	2.42E-11	0.403	0.013	0.011	0.220315	0.403	0.011	0.012	0.355068	0.403	0.003	0.024	0.887343
Atrial fibrillation || id:ebi-a-GCST006414	1030836	rs6790396	3	38771925	G	C	0.596	0.063	0.007	2.40E-20	0.585	-0.005	0.010	0.660274	0.585	-0.001	0.012	0.918171	0.585	0.019	0.023	0.412868
Atrial fibrillation || id:ebi-a-GCST006414	1030836	rs67969609	2	145760353	G	C	0.071	0.071	0.013	1.71E-08	0.069	0.028	0.020	0.173412	0.069	0.012	0.023	0.593549	0.069	0.016	0.045	0.716757
Atrial fibrillation || id:ebi-a-GCST006414	1030836	rs6838973	4	111765495	T	C	0.441	-0.151	0.007	1.03E-111	0.430	0.013	0.010	0.213755	0.430	0.000	0.012	0.980772	0.430	0.027	0.023	0.243181
Atrial fibrillation || id:ebi-a-GCST006414	1030836	rs6882776	5	172664163	A	G	0.284	-0.071	0.007	9.64E-22	0.275	-0.006	0.012	0.579649	0.275	-0.025	0.013	0.0584431	0.275	0.023	0.026	0.379379
Atrial fibrillation || id:ebi-a-GCST006414	1030836	rs6994744	8	141740868	C	A	0.495	0.041	0.007	1.09E-09	0.485	0.002	0.010	0.827066	0.485	-0.018	0.012	0.12226	0.485	0.027	0.023	0.241859
Atrial fibrillation || id:ebi-a-GCST006414	1030836	rs71454237	12	70013415	A	G	0.209	-0.062	0.008	1.78E-13	0.206	-0.020	0.013	0.12823	0.206	-0.015	0.014	0.295176	0.206	-0.040	0.029	0.162051
Atrial fibrillation || id:ebi-a-GCST006414	1030836	rs7172038	15	73667255	G	T	0.160	0.112	0.009	4.78E-36	0.160	-0.011	0.014	0.435165	0.160	0.005	0.016	0.730677	0.160	-0.010	0.031	0.747074
Atrial fibrillation || id:ebi-a-GCST006414	1030836	rs7224711	17	76772288	T	C	0.522	-0.037	0.007	3.72E-08	0.522	-0.022	0.010	0.0322577	0.522	-0.018	0.012	0.11227	0.522	-0.015	0.023	0.506324
Atrial fibrillation || id:ebi-a-GCST006414	1030836	rs7225165	17	1309850	A	G	0.113	-0.066	0.011	3.20E-09	0.115	-0.009	0.016	0.563344	0.115	-0.013	0.018	0.474856	0.115	-0.019	0.036	0.605165
Atrial fibrillation || id:ebi-a-GCST006414	1030836	rs72694603	1	112458893	T	C	0.315	-0.055	0.007	2.26E-14	0.318	0.008	0.011	0.484251	0.318	0.004	0.012	0.755988	0.318	0.021	0.025	0.401584
Atrial fibrillation || id:ebi-a-GCST006414	1030836	rs72700114	1	170193825	C	G	0.076	0.202	0.013	3.29E-54	0.075	-0.006	0.020	0.758297	0.075	0.013	0.022	0.563281	0.075	-0.021	0.044	0.639969
Atrial fibrillation || id:ebi-a-GCST006414	1030836	rs72811294	17	12618680	C	G	0.113	-0.072	0.011	9.67E-12	0.112	0.002	0.016	0.886473	0.112	-0.001	0.018	0.977461	0.112	0.028	0.037	0.446102
Atrial fibrillation || id:ebi-a-GCST006414	1030836	rs72926475	2	86594487	A	G	0.123	-0.068	0.010	2.37E-11	0.124	0.027	0.016	0.0796058	0.124	0.016	0.018	0.367463	0.124	0.008	0.035	0.818342
Atrial fibrillation || id:ebi-a-GCST006414	1030836	rs72966339	6	122398241	T	C	0.368	-0.062	0.007	7.42E-19	0.355	0.008	0.011	0.459616	0.355	0.008	0.012	0.524297	0.355	0.011	0.024	0.633387
Atrial fibrillation || id:ebi-a-GCST006414	1030836	rs73041705	3	24463235	C	T	0.299	-0.044	0.007	1.55E-09	0.300	-0.003	0.011	0.798515	0.300	-0.011	0.013	0.362147	0.300	0.027	0.025	0.285428
Atrial fibrillation || id:ebi-a-GCST006414	1030836	rs73366713	6	16415751	A	G	0.140	-0.104	0.010	1.53E-25	0.143	-0.002	0.015	0.895216	0.143	-0.018	0.016	0.263642	0.143	0.029	0.033	0.377957
Atrial fibrillation || id:ebi-a-GCST006414	1030836	rs74500426	4	174642789	T	G	0.076	-0.092	0.013	4.29E-13	0.075	0.015	0.020	0.438404	0.075	0.000	0.022	0.988425	0.075	-0.060	0.044	0.168263
Atrial fibrillation || id:ebi-a-GCST006414	1030836	rs74832855	1	170171598	G	A	0.037	0.122	0.018	1.43E-11	0.034	-0.004	0.028	0.899427	0.034	0.002	0.032	0.952097	0.034	0.061	0.063	0.338523
Atrial fibrillation || id:ebi-a-GCST006414	1030836	rs74884082	14	73249419	T	C	0.250	-0.049	0.008	3.48E-10	0.250	0.000	0.012	0.983298	0.250	0.001	0.013	0.969499	0.250	0.009	0.027	0.725769
Atrial fibrillation || id:ebi-a-GCST006414	1030836	rs74910854	7	74110705	G	A	0.069	0.090	0.016	4.31E-08	0.068	0.007	0.021	0.71955	0.068	-0.011	0.023	0.636176	0.068	0.028	0.046	0.540637
Atrial fibrillation || id:ebi-a-GCST006414	1030836	rs7508	8	17913970	A	G	0.711	0.071	0.008	1.69E-21	0.718	-0.004	0.012	0.75383	0.718	0.010	0.013	0.426025	0.718	-0.010	0.026	0.700068
Atrial fibrillation || id:ebi-a-GCST006414	1030836	rs7529220	1	22282619	C	T	0.847	0.062	0.010	1.98E-10	0.858	-0.045	0.015	0.00273754	0.859	-0.040	0.017	0.0165171	0.859	-0.039	0.034	0.253634
Atrial fibrillation || id:ebi-a-GCST006414	1030836	rs7574892	2	175512820	A	G	0.485	0.055	0.007	1.98E-16	0.482	-0.008	0.010	0.438987	0.482	-0.006	0.012	0.633725	0.482	-0.017	0.023	0.452232
Atrial fibrillation || id:ebi-a-GCST006414	1030836	rs76097649	11	128764570	A	G	0.093	0.115	0.012	1.26E-20	0.089	0.037	0.018	0.0399419	0.089	0.016	0.020	0.4197	0.089	0.082	0.039	0.0383402
Atrial fibrillation || id:ebi-a-GCST006414	1030836	rs7612445	3	179172979	T	G	0.188	0.049	0.008	4.81E-09	0.188	0.007	0.013	0.613029	0.188	0.009	0.015	0.566882	0.188	-0.032	0.030	0.286197
Atrial fibrillation || id:ebi-a-GCST006414	1030836	rs77316573	16	2265271	T	C	0.199	0.053	0.009	3.26E-09	0.182	0.007	0.013	0.60469	0.182	0.003	0.015	0.856544	0.182	-0.002	0.030	0.940134
Atrial fibrillation || id:ebi-a-GCST006414	1030836	rs775498	12	70071513	G	A	0.280	0.042	0.007	1.05E-08	0.286	-0.001	0.011	0.913432	0.286	-0.015	0.013	0.240121	0.286	0.018	0.025	0.489729
Atrial fibrillation || id:ebi-a-GCST006414	1030836	rs7789146	7	150661409	A	G	0.179	-0.058	0.009	2.12E-11	0.174	0.007	0.014	0.618854	0.174	0.002	0.015	0.875914	0.174	0.007	0.030	0.815697
Atrial fibrillation || id:ebi-a-GCST006414	1030836	rs7915134	10	75420180	T	C	0.144	-0.117	0.010	1.42E-34	0.142	-0.003	0.015	0.843394	0.142	-0.008	0.017	0.635872	0.142	0.041	0.033	0.218275
Atrial fibrillation || id:ebi-a-GCST006414	1030836	rs79187193	1	147255831	A	G	0.057	-0.116	0.015	3.15E-14	0.052	-0.015	0.023	0.532235	0.052	-0.029	0.026	0.260744	0.052	0.007	0.052	0.897939
Atrial fibrillation || id:ebi-a-GCST006414	1030836	rs8088085	18	48708548	C	A	0.465	-0.037	0.007	4.79E-08	0.453	-0.021	0.010	0.0460435	0.453	-0.023	0.012	0.0503132	0.453	0.007	0.023	0.752974
Atrial fibrillation || id:ebi-a-GCST006414	1030836	rs883079	12	114793240	T	C	0.707	0.098	0.007	2.84E-40	0.733	0.006	0.012	0.61211	0.733	0.004	0.013	0.786533	0.733	-0.004	0.026	0.887094
Atrial fibrillation || id:ebi-a-GCST006414	1030836	rs9506925	13	23368943	T	C	0.267	0.045	0.008	2.72E-09	0.279	-0.005	0.012	0.672964	0.279	-0.020	0.013	0.118957	0.279	-0.002	0.026	0.944359
Atrial fibrillation || id:ebi-a-GCST006414	1030836	rs9953366	18	46474192	C	T	0.663	0.049	0.007	1.82E-11	0.677	-0.003	0.011	0.765792	0.677	-0.025	0.012	0.043438	0.677	0.025	0.025	0.317995
Myocardial infarction || id:ieu-a-798	171875	rs10455872	6	161010118	G	A	0.054	0.285	0.027	9.23E-27	0.079	-0.011	0.019	0.581557	0.079	-0.021	0.021	0.324018	0.079	-0.038	0.043	0.376569
Myocardial infarction || id:ieu-a-798	171875	rs10947786	6	39156410	A	G	0.208	-0.072	0.013	1.77E-08	0.214	0.001	0.013	0.941661	0.214	0.001	0.014	0.970625	0.214	-0.006	0.028	0.817776
Myocardial infarction || id:ieu-a-798	171875	rs113113862	19	11183577	A	G	0.218	-0.075	0.013	2.78E-09	0.258	-0.023	0.012	0.0510885	0.258	-0.029	0.013	0.0290736	0.259	0.004	0.026	0.878839
Myocardial infarction || id:ieu-a-798	171875	rs11556924	7	129663496	T	C	0.298	-0.069	0.013	4.00E-08	0.389	0.018	0.011	0.0923961	0.389	0.026	0.012	0.0255983	0.389	0.042	0.024	0.072667
Myocardial infarction || id:ieu-a-798	171875	rs1332329	10	91003419	C	A	0.362	0.079	0.011	2.68E-13	0.347	-0.009	0.011	0.385707	0.346	-0.028	0.012	0.0213706	0.347	-0.023	0.024	0.345609
Myocardial infarction || id:ieu-a-798	171875	rs1510226	6	160816409	C	T	0.027	0.226	0.035	6.16E-11	0.019	0.000	0.038	0.99222	0.019	0.031	0.042	0.45765	0.019	0.001	0.085	0.99107
Myocardial infarction || id:ieu-a-798	171875	rs1870634	10	44480811	G	T	0.618	0.070	0.011	8.35E-11	0.665	-0.007	0.011	0.548147	0.665	-0.006	0.012	0.648866	0.665	-0.023	0.024	0.337416
Myocardial infarction || id:ieu-a-798	171875	rs2019090	11	103668962	T	A	0.642	-0.065	0.011	3.65E-09	0.706	0.009	0.011	0.450604	0.706	-0.001	0.013	0.921174	0.706	-0.003	0.025	0.907491
Myocardial infarction || id:ieu-a-798	171875	rs2327426	6	134202690	C	T	0.298	-0.063	0.011	1.22E-08	0.300	-0.006	0.011	0.611927	0.300	-0.008	0.013	0.512449	0.300	-0.054	0.025	0.0335915
Myocardial infarction || id:ieu-a-798	171875	rs2505083	10	30335122	C	T	0.395	0.061	0.011	6.93E-09	0.431	0.016	0.010	0.125965	0.431	0.027	0.012	0.0194613	0.431	0.009	0.023	0.69878
Myocardial infarction || id:ieu-a-798	171875	rs2681472	12	90008959	G	A	0.194	0.073	0.013	6.11E-09	0.172	-0.012	0.014	0.36039	0.172	-0.014	0.015	0.369685	0.172	0.031	0.031	0.314914
Myocardial infarction || id:ieu-a-798	171875	rs28451064	21	35593827	A	G	0.119	0.122	0.018	6.10E-12	0.131	-0.019	0.016	0.225103	0.131	0.004	0.017	0.812537	0.131	-0.040	0.035	0.247547
Myocardial infarction || id:ieu-a-798	171875	rs35700460	1	222811407	G	A	0.647	0.082	0.012	1.91E-11	0.713	-0.004	0.011	0.728675	0.713	-0.001	0.013	0.925148	0.713	-0.016	0.026	0.51895
Myocardial infarction || id:ieu-a-798	171875	rs41290120	19	45382675	A	G	0.032	-0.186	0.031	2.62E-09	0.048	0.001	0.024	0.963657	0.048	-0.007	0.027	0.800693	0.048	0.028	0.054	0.602777
Myocardial infarction || id:ieu-a-798	171875	rs4773141	13	110954353	G	C	0.345	0.080	0.013	4.69E-10	0.359	0.009	0.011	0.416194	0.359	0.001	0.012	0.943235	0.359	0.039	0.024	0.108009
Myocardial infarction || id:ieu-a-798	171875	rs4977574	9	22098574	G	A	0.482	0.189	0.010	4.58E-75	0.489	-0.011	0.010	0.268323	0.489	-0.010	0.012	0.380276	0.490	0.052	0.023	0.0221946
Myocardial infarction || id:ieu-a-798	171875	rs532436	9	136149830	A	G	0.188	0.111	0.013	2.36E-17	0.188	-0.027	0.013	0.0457737	0.188	-0.018	0.015	0.231473	0.188	-0.029	0.030	0.33483
Myocardial infarction || id:ieu-a-798	171875	rs653178	12	112007756	T	C	0.558	-0.077	0.012	2.84E-11	0.516	-0.010	0.010	0.319745	0.516	0.003	0.012	0.771963	0.516	-0.013	0.023	0.575303
Myocardial infarction || id:ieu-a-798	171875	rs7173743	15	79141784	C	T	0.432	-0.064	0.010	7.75E-10	0.457	0.015	0.010	0.144498	0.457	0.016	0.012	0.160291	0.457	-0.011	0.023	0.625457
Myocardial infarction || id:ieu-a-798	171875	rs72689147	4	156639888	T	G	0.180	-0.074	0.013	1.65E-08	0.183	0.001	0.013	0.938324	0.183	-0.005	0.015	0.74335	0.183	0.034	0.030	0.251525
Myocardial infarction || id:ieu-a-798	171875	rs72934535	2	203968973	C	T	0.093	0.141	0.019	3.36E-14	0.108	-0.012	0.017	0.459905	0.108	-0.026	0.019	0.165298	0.108	-0.070	0.037	0.0599545
Myocardial infarction || id:ieu-a-798	171875	rs7528419	1	109817192	G	A	0.202	-0.101	0.013	9.92E-16	0.222	-0.004	0.012	0.775364	0.222	-0.018	0.014	0.206967	0.222	0.017	0.028	0.529929
Myocardial infarction || id:ieu-a-798	171875	rs9349379	6	12903957	G	A	0.411	0.131	0.011	9.37E-35	0.406	0.014	0.010	0.178861	0.406	0.004	0.012	0.759284	0.406	0.016	0.023	0.492663
Myocardial infarction || id:ieu-a-798	171875	rs9970807	1	56965664	T	C	0.084	-0.111	0.018	1.81E-09	0.092	-0.029	0.018	0.107799	0.092	-0.044	0.020	0.0303651	0.092	-0.069	0.040	0.083165
Stroke || id:ebi-a-GCST006906	446696	rs10774624	12	111833788	A	G	0.529	-0.065	0.009	4.04E-12	0.510	-0.006	0.010	0.536811	0.511	0.007	0.012	0.560758	0.510	-0.016	0.023	0.482513
Stroke || id:ebi-a-GCST006906	446696	rs11242678	6	1337180	T	C	0.255	0.064	0.011	8.71E-10	0.248	0.012	0.012	0.319561	0.248	0.011	0.013	0.406254	0.248	-0.006	0.027	0.829811
Stroke || id:ebi-a-GCST006906	446696	rs1537375	9	22116071	C	T	0.502	0.052	0.009	1.24E-08	0.502	-0.009	0.010	0.366302	0.502	-0.003	0.012	0.770725	0.502	0.045	0.023	0.0543492
Stroke || id:ebi-a-GCST006906	446696	rs2107595	7	19049388	A	G	0.167	0.080	0.012	3.59E-11	0.153	-0.004	0.014	0.795423	0.153	-0.005	0.016	0.758084	0.153	-0.001	0.032	0.969183
Stroke || id:ebi-a-GCST006906	446696	rs2634074	4	111677041	A	T	0.789	-0.084	0.011	6.56E-14	0.798	0.012	0.013	0.350405	0.798	0.009	0.015	0.556812	0.798	-0.005	0.029	0.875208
Stroke || id:ebi-a-GCST006906	446696	rs475937	11	102687700	C	A	0.868	-0.076	0.014	2.92E-08	0.867	0.008	0.016	0.601353	0.867	0.007	0.018	0.692253	0.867	0.026	0.035	0.453563
Stroke || id:ebi-a-GCST006906	446696	rs4942561	13	47209347	T	G	0.758	0.064	0.011	2.05E-09	0.752	0.007	0.012	0.578602	0.752	0.009	0.013	0.492621	0.752	-0.002	0.027	0.935977
Ischemic stroke (large artery atherosclerosis) || id:ebi-a-GCST006907	410484	rs10820405	9	106010237	A	G	0.185	-0.181	0.033	4.51E-08	0.186	-0.014	0.013	0.282694	0.186	-0.011	0.015	0.478905	0.186	-0.033	0.030	0.261162
Ischemic stroke (large artery atherosclerosis) || id:ebi-a-GCST006907	410484	rs2107595	7	19049388	A	G	0.168	0.236	0.032	1.44E-13	0.153	-0.004	0.014	0.795423	0.153	-0.005	0.016	0.758084	0.153	-0.001	0.032	0.969183
Ischemic stroke (large artery atherosclerosis) || id:ebi-a-GCST006907	410484	rs476762	11	102710707	A	T	0.133	0.201	0.035	1.22E-08	0.126	-0.006	0.016	0.680375	0.126	-0.004	0.017	0.798834	0.126	-0.027	0.035	0.436921
Ischemic stroke || id:ebi-a-GCST006908	440328	rs11242678	6	1337180	T	C	0.255	0.072	0.011	2.70E-10	0.248	0.012	0.012	0.319561	0.248	0.011	0.013	0.406254	0.248	-0.006	0.027	0.829811
Ischemic stroke || id:ebi-a-GCST006908	440328	rs2066864	4	155525695	A	G	0.245	0.063	0.012	3.51E-08	0.238	0.017	0.012	0.150097	0.237	0.014	0.014	0.316312	0.238	0.064	0.027	0.0163938
Ischemic stroke || id:ebi-a-GCST006908	440328	rs2107595	7	19049388	A	G	0.167	0.088	0.013	2.33E-11	0.153	-0.004	0.014	0.795423	0.153	-0.005	0.016	0.758084	0.153	-0.001	0.032	0.969183
Ischemic stroke || id:ebi-a-GCST006908	440328	rs2634074	4	111677041	A	T	0.788	-0.094	0.012	5.90E-15	0.798	0.012	0.013	0.350405	0.798	0.009	0.015	0.556812	0.798	-0.005	0.029	0.875208
Ischemic stroke || id:ebi-a-GCST006908	440328	rs2758612	1	156205301	C	T	0.355	-0.065	0.011	3.68E-09	0.355	0.002	0.011	0.841205	0.355	0.014	0.012	0.248058	0.355	0.015	0.024	0.53727
Ischemic stroke || id:ebi-a-GCST006908	440328	rs3184504	12	111884608	C	T	0.528	-0.078	0.010	1.23E-14	0.516	-0.012	0.010	0.25318	0.517	0.001	0.012	0.904727	0.517	-0.015	0.023	0.502906
Ischemic stroke || id:ebi-a-GCST006908	440328	rs473238	11	102700360	C	T	0.867	-0.083	0.015	1.65E-08	0.867	0.006	0.016	0.676741	0.867	0.005	0.017	0.787414	0.867	0.026	0.035	0.44913
Ischemic stroke || id:ebi-a-GCST006908	440328	rs4942561	13	47209347	T	G	0.759	0.066	0.012	1.77E-08	0.752	0.007	0.012	0.578602	0.752	0.009	0.013	0.492621	0.752	-0.002	0.027	0.935977
Heart failure || id:ebi-a-GCST009541	977323	rs11745324	5	137012171	A	G	NA	-0.053	0.010	2.34E-08	0.232	0.021	0.012	0.0922312	0.232	0.003	0.014	0.825409	0.232	0.066	0.027	0.0145684
Heart failure || id:ebi-a-GCST009541	977323	rs1510226	6	160816409	C	T	NA	0.162	0.029	1.27E-08	0.019	0.000	0.038	0.99222	0.019	0.031	0.042	0.45765	0.019	0.001	0.085	0.99107
Heart failure || id:ebi-a-GCST009541	977323	rs17042102	4	111668626	A	G	NA	0.110	0.012	5.71E-20	0.103	-0.013	0.017	0.433048	0.103	-0.004	0.019	0.845491	0.103	-0.023	0.038	0.54668
Heart failure || id:ebi-a-GCST009541	977323	rs17617337	10	121426884	T	C	NA	-0.056	0.010	3.65E-09	0.214	0.017	0.013	0.183589	0.214	0.012	0.014	0.385358	0.214	0.014	0.028	0.609427
Heart failure || id:ebi-a-GCST009541	977323	rs4135240	6	36647680	C	T	NA	-0.049	0.008	6.84E-09	0.332	-0.008	0.011	0.438751	0.332	-0.005	0.012	0.70656	0.332	-0.021	0.024	0.38268
Heart failure || id:ebi-a-GCST009541	977323	rs55730499	6	161005610	T	C	NA	0.106	0.016	1.83E-11	0.080	-0.012	0.019	0.519896	0.080	-0.023	0.021	0.287672	0.080	-0.039	0.042	0.359387
Heart failure || id:ebi-a-GCST009541	977323	rs56094641	16	53806453	G	A	NA	0.045	0.008	1.21E-08	0.402	-0.010	0.011	0.33023	0.402	-0.017	0.012	0.140568	0.402	-0.046	0.024	0.0495842
Heart failure || id:ebi-a-GCST009541	977323	rs600038	9	136151806	C	T	NA	0.057	0.010	3.68E-09	0.210	-0.020	0.013	0.123633	0.210	-0.013	0.014	0.354253	0.210	-0.013	0.028	0.636705
Heart failure || id:ebi-a-GCST009541	977323	rs660240	1	109817838	C	T	NA	0.061	0.010	3.25E-10	0.784	0.008	0.013	0.534142	0.784	0.024	0.014	0.0916898	0.784	-0.021	0.028	0.461532
Ischemic stroke (cardioembolic) || id:ebi-a-GCST006910	413304	rs12932445	16	73069888	C	T	0.181	0.176	0.025	6.88E-13	0.168	0.002	0.014	0.858714	0.168	0.009	0.015	0.550625	0.168	-0.012	0.031	0.707094
Ischemic stroke (cardioembolic) || id:ebi-a-GCST006910	413304	rs146390073	1	241306248	T	C	0.022	0.669	0.120	2.20E-08	0.019	0.014	0.040	0.729221	0.019	0.022	0.045	0.623967	0.019	0.018	0.090	0.843213
Ischemic stroke (cardioembolic) || id:ebi-a-GCST006910	413304	rs2466455	4	111685615	T	C	0.783	-0.299	0.022	2.75E-41	0.799	0.012	0.013	0.342779	0.799	0.009	0.015	0.551387	0.799	-0.005	0.029	0.857735
Ischemic stroke (cardioembolic) || id:ebi-a-GCST006910	413304	rs6838973	4	111765495	T	C	0.434	-0.108	0.020	3.58E-08	0.430	0.013	0.010	0.213755	0.430	0.000	0.012	0.980772	0.430	0.027	0.023	0.243181

Abbreviations: SNP, Single-nucleotide polymorphism; CHR, Chromosome; Pos, Position; EA, Effect Allele; OA, Other Allele; EAF, effect allele frequency; SE, standard error; P, P-value; CVD, Cardiovascular disease and CHIP, clonal hematopoiesis of indeterminate potential
